# TRPC channels blockade abolishes endotoxemic cardiac dysfunction by hampering intracellular inflammation and Ca^2+^ leakage

**DOI:** 10.1038/s41467-022-35242-0

**Published:** 2022-12-02

**Authors:** Na Tang, Wen Tian, Guang-Yuan Ma, Xiong Xiao, Lei Zhou, Ze-Zhi Li, Xiao-Xiao Liu, Chong-Yao Li, Ke-Han Wu, Wenjuan Liu, Xue-Ying Wang, Yuan-Yuan Gao, Xin Yang, Jianzhao Qi, Ding Li, Yang Liu, Wen-Sheng Chen, Jinming Gao, Xiao-Qiang Li, Wei Cao

**Affiliations:** 1 Department of Pharmacy, School of Chemistry & Pharmacy, Northwest A&F University, Yangling, Shaanxi China; 2 Shaanxi Key Laboratory of Natural Products & Chemical Biology, Northwest A&F University, Yangling, Shaanxi China; 3 Department of Pharmacology, School of Pharmacy, Fourth Military Medical University, Xi’an, Shaanxi China; 4 Key Laboratory of Gastrointestinal Pharmacology of Chinese Materia Medica of the State Administration of Traditional Chinese Medicine, Fourth Military Medical University, Xi’an, Shaanxi China; 5 Department of Pharmacy, Xi’an No.3 Hospital, the Affiliated Hospital of Northwest University, Xi’an, Shaanxi China; 6 Department of Cardiovascular Surgery, Xijing Hospital, Fourth Military Medical University, Xi’an, Shaanxi China; 7 Department of Cardiovascular Surgery, Xi’an Gaoxin Hospital, Xi’an, Shaanxi China

**Keywords:** Drug development, Cardiomyopathies

## Abstract

Intracellular Ca^2+^ dysregulation is a key marker in septic cardiac dysfunction; however, regulation of the classic Ca^2+^ regulatory modules cannot successfully abolish this symptom. Here we show that the knockout of transient receptor potential canonical (TRPC) channel isoforms TRPC1 and TRPC6 can ameliorate LPS-challenged heart failure and prolong survival in mice. The LPS-triggered Ca^2+^ release from the endoplasmic reticulum both in cardiomyocytes and macrophages is significantly inhibited by *Trpc1* or *Trpc6* knockout. Meanwhile, TRPC’s molecular partner — calmodulin — is uncoupled during *Trpc1* or *Trpc6* deficiency and binds to TLR4’s Pococurante site and atypical isoleucine-glutamine-like motif to block the inflammation cascade. Blocking the C-terminal CaM/IP3R binding domain in TRPC with chemical inhibitor could obstruct the Ca^2+^ leak and TLR4-mediated inflammation burst, demonstrating a cardioprotective effect in endotoxemia and polymicrobial sepsis. Our findings provide insight into the pathogenesis of endotoxemic cardiac dysfunction and suggest a novel approach for its treatment.

## Abstract (web summary)

TRPCs, nonselective cation channels, are involved in cardiac contraction and conduction. Here, the authors show that *Trpc1*/*6* deficiency or pharmacological inhibition improves endotoxemic cardiac dysfunction and prolongs survival by prominently suppressing cardiac inflammation and ER Ca^2+^ release.

## Introduction

Endotoxemia (ETM) or sepsis is a dire issue of modern critical care medicine that can progress to multiple organ failure and death^1^. Cardiac dysfunction (also called ETM-induced cardiomyopathy) is recognized as one of the most critical syndromes of ETM that occurs in almost 40–50% of patients^2^. The mortality rate of ETM with cardiac dysfunction can even reach 70–90%^3^. Bacterial endotoxin or lipopolysaccharide (LPS), a pathogen released by Gram-negative bacteria, as an extremely strong stimulator of inflammatory reactions plays a major role in the development of ETM^4^. Although antibiotics and volume replacement are the cornerstones of current therapy in ETM, an overwhelming inflammatory response limits their effectiveness, and no specific therapies available to treat cardiac dysfunction to this day^5^.

Cardiac dysfunction associated with ETM is generally characterized by contractile defects and impaired myocardial compliance, along with excessive cardiac inflammation and damaged mitochondria^6^. The hallmark of contractile defects in ETM is myocyte mishandling of Ca^2+^, leading to the disruption of intracellular free Ca^2+^ concentration ([Ca^2+^]_i_)^7^. Increased [Ca^2+^]_i_ has been observed in cardiomyocytes and animal hearts that were directly exposed to LPS^8,9^. Since the Ca^2+^ binding to the myofilament protein troponin C switches on the contractile machinery^10^, Ca^2+^ regulating agents seem to be a promising approach to treat ETM^11^. However, targeting the common cellular Ca^2+^ regulatory apparatuses, including L-type Ca^2+^ channel, ryanodine receptors (RyRs), sarco/endoplasmic reticulum Ca^2+^-ATPase, and Na^+^/Ca^2+^-exchanger could not successfully protect against septic cardiac dysfunction in mice^12,13^. A comprehensive understanding of the regulatory mechanisms that handle abnormal Ca^2+^ is still missing. Therefore, the real identity and mechanism of the Ca^2+^ regulatory apparatuses responsible for the LPS-triggered aberrant intracellular Ca^2+^ homeostasis are worth investigating in the areas of biology and medicine.

Unlike voltage-dependent calcium channels, transient receptor potential (TRP) proteins constitute a vast non-voltage-gated cation channel superfamily that can integrate multiple stimuli and transduce their activity to downstream cellular signal pathways via Ca^2+^ entry and/or membrane depolarization^14^. The canonical or classical transient receptor potential (TRPC) channels, as the prominent nonselective Ca^2+^-permeable cation channels in this channel superfamily, play a key role in regulating cardiac contraction and conduction under pathological conditions^15^. TRPC channels can form functional homo- and hetero-tetramers within two defined subgroups (TRPC1/4/5 and TRPC3/6/7), excluding TRPC2, which is a pseudogene in humans^16^. In general, TRPC1/4/5 can be activated by depletion of intracellular Ca^2+^ stores (store-operated Ca^2+^ entry, SOCE), and TRPC3/6/7 are activated by diacylglycerol generated by G protein-coupled receptors/Gαq/phospholipase C signaling. Once activated, TRPC channels induce signal transduction through [Ca^2+^]_i_ elevations or refilling of sarcoplasmic reticulum (SR) or endoplasmic reticulum (ER) Ca^2+^ stores, which is required for essential hypertension, cardiac hypertrophy, and heart failure^17^. Enhanced Ca^2+^ leak exposed to LPS is mainly regulated by the SR/ER^18,19^, so TRPC channels, at least TRPC1/4/5, might be involved in the pathogenesis of septic cardiac dysfunction. To date, whether and how different TRPC channels regulate LPS-triggered Ca^2+^ influx is unclear. Moreover, TRPC channels participate in regulating calcineurin activity and nuclear factor of activated T-cells (NFAT) translocation to promote inflammatory gene expression^17^. In this context, TRPC channels differ from the other mentioned Ca^2+^ regulatory apparatuses in rendering potential inflammation regulatory effects in septic cardiac dysfunction therapy; thus, a systematic assessment and mechanism study are necessary.

In the present study, we demonstrate that *Trpc1* or *Trpc6* knockout significantly protects LPS-induced cardiac dysfunction and prolong the survival of mice through inhibiting Ca^2+^ leakage from SR/ER and inflammation cascade in endotoxemic hearts. *Trpc1* or *Trpc6* knockout markedly inhibited IP3R-mediated Ca^2+^ release from the SR/ER in response to the LPS challenge both in cardiomyocytes and macrophages. We also uncovered a mechanism that involves TRPC’s molecular partner, calmodulin (CaM) disrupted myeloid differentiation primary response protein 88 (MyD88)- and Toll/interleukin-1 receptor (TIR) domain-containing adaptor inducing IFN-β (TRIF)-mediated inflammation cascade during *Trpc1* or *Trpc6* deficiency; owing to binding with Pococurante (Poc) site and atypical isoleucine-glutamine (IQ)-like the motif of the TIR domain in Toll-like receptor 4 (TLR4) protein. Specifically, pharmacological inhibition of TRPCs exhibited significant cardioprotective effects amid the development of ETM and polymicrobial sepsis. These data provide proof-of-principle that targeting TRPC channels has potential as an ETM therapy.

## Results

### TRPC1 and TRPC6 are important TRPC isoforms highly expressed in LPS-challenged hearts and boost endotoxemic cardiac dysfunction

**Fig. 1:**
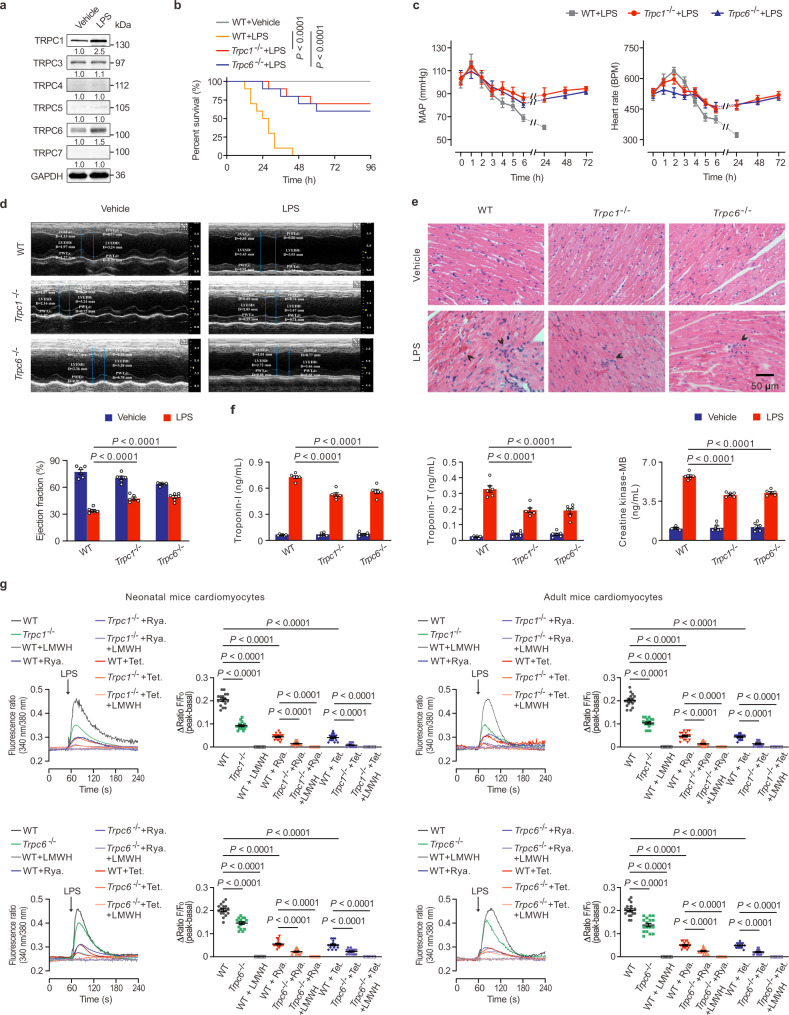
The *Trpc1* or *Trpc6* knockout protects endotoxemic hearts. **a** The protein expressions of TRPCs in the ventricles of LPS-challenged mice (pooled tissues from 3 male mice per sample, *n* = 3 biological independent experiments). **b** Kaplan-Meier survival curves of the WT, *Trpc1*^−/−^, and *Trpc6*^−/−^ mice (*n* = 10 male mice per group). Statistical significance was determined using the log-rank test. Exact *P* value = 6.5 × 10^−5^ (WT + LPS vs *Trpc1*^−/−^ + LPS), 9.1 × 10^−5^ (WT + LPS vs *Trpc6*^−/−^ + LPS). **c** The mean arterial blood pressure (MAP) and heart rate of the LPS-challenged WT, *Trpc1*^−/−^, and *Trpc6*^−/−^ mice during 6 h (mean ± SEM, *n* = 8 male mice per group). **d** Representative M-mode echocardiography still and the statistical analysis of ejection fraction in WT, *Trpc1*^−/−^, and *Trpc6*^−/−^ mice 6 h after LPS challenge (mean ± SEM, *n* = 6 male mice per group). Statistical significance was determined using the one-way ANOVA with Tukey’s multiple comparisons test. Exact *P* value = 8.4 × 10^−5^ (WT + LPS vs *Trpc1*^−/−^ + LPS), 1.3 × 10^−5^ (WT + LPS vs *Trpc6*^−/−^ + LPS). **e** Representative photomicrographs of ventricular tissues stained with hematoxylin and eosin. Black arrows indicate the myocardial interstitial edema associated with the mononuclear inflammatory cells infiltration (*n* = 6 images from 3 male mice per group). **f** The levels of serum cardiac troponin-T, troponin-I, and creatine kinase-MB in the mice challenged with LPS (mean ± SEM, *n* = 6 male mice samples per group). Statistical significance was determined using the one-way ANOVA with Tukey’s multiple comparisons test. Troponin-I, exact *P* value = 7.8 × 10^−9^ (WT + LPS vs *Trpc1*^−/−^ + LPS), 4.5 × 10^−7^ (WT + LPS vs *Trpc6*^−/−^ + LPS). Troponin-T, exact *P* value = 9.3 × 10^−7^ (WT + LPS vs *Trpc1*^−/−^ + LPS), 3.8 × 10^−7^ (WT + LPS vs *Trpc6*^−/−^ + LPS). Creatine kinase-MB, exact *P* value = 1.9 × 10^−10^ (WT + LPS vs *Trpc1*^−/−^ + LPS), 2.7 × 10^−9^ (WT + LPS vs *Trpc6*^−/−^ + LPS). **g** The effects of TRPC1 and TRPC6 on IP3Rs and RyRs-regulated Ca^2+^ release in LPS-induced mice cardiomyocytes. Neonatal and adult mice cardiomyocytes in Ca^2+^-free extracellular solution were pre-incubated with ryanodine (Rya.), tetracaine (Tet.), and/or low molecular weight heparin (LMWH), and then treated with LPS to measure [Ca^2+^]_i_ levels using a digital wide-field fluorescence imaging system. Typical trace recordings (left panel) and the statistical analysis (right panel) are shown (mean ± SEM, *n* = 15–20 cells from 3 mice per group). Statistical significance was determined using the one-way ANOVA with Games Howell’s multiple comparisons test. In neonatal mice cardiomyocytes, exact *P* value = 8.6 × 10^−12^ (WT vs *Trpc1*^−/−^), 1.5 × 10^−12^ (WT vs WT + LMWH), 9.5 × 10^−13^ (WT vs WT + Rya.), 9.0 × 10^−13^ (WT vs WT + Tet.), 3.0 × 10^−6^ (WT + Rya. vs *Trpc1*^−/−^ + Rya.), 4.1 × 10^−8^ (WT + Rya. vs *Trpc1*^−/−^ + Rya. + LMWH), 6.1 × 10^−8^ (WT + Tet. vs *Trpc1*^−/−^ + Tet.), 9.8 × 10^−9^ (WT + Tet. vs *Trpc1*^−/−^ + Tet. + LMWH), 8.0 × 10^−6^ (WT vs *Trpc6*^−/−^), 1.3 × 10^−12^ (WT vs WT + LMWH), 9.0 × 10^−13^ (WT vs WT + Rya.), 8.7 × 10^−13^ (WT vs WT + Tet.), 3.0 × 10^−6^ (WT + Rya. vs *Trpc6*^−/−^ + Rya.), 4.3 × 10^−9^ (WT + Rya. vs *Trpc6*^−/−^+Rya.+LMWH), 8.6 × 10^−5^ (WT + Tet. vs *Trpc6*^−/−^+Tet.), and 3.2 × 10^−8^ (WT + Tet. vs *Trpc6*^−/−^+Tet.+LMWH). In adult mice cardiomyocytes, exact *P* value = 4.1 × 10^−11^ (WT vs *Trpc1*^−/−^), 1.1 × 10^−12^ (WT vs WT + LMWH), 8.5 × 10^−13^ (WT vs WT + Rya.), 9.4 × 10^−13^ (WT vs WT + Tet.), 2.7 × 10^−5^ (WT + Rya. vs *Trpc1*^−/−^+Rya.), 5.2 × 10^−7^ (WT + Rya. vs *Trpc1*^−/−^+Rya.+LMWH), 1.2 × 10^−7^ (WT + Tet. vs *Trpc1*^−/−^+Tet.), 7.8 × 10^−10^ (WT + Tet. vs *Trpc1*^−/−^+Tet.+LMWH), 1.3 × 10^−5^ (WT vs *Trpc6*^−/−^), 1.2 × 10^−12^ (WT vs WT + LMWH), 8.9 × 10^−13^ (WT vs WT + Rya.), 1.1 × 10^−12^ (WT vs WT + Tet.), 8.3 × 10^−5^ (WT + Rya. vs *Trpc6*^−/−^+Rya.), 8.5 × 10^−9^ (WT + Rya. vs *Trpc6*^−/−^+Rya.+LMWH), 2.7 × 10^−7^ (WT + Tet. vs *Trpc6*^−/−^+Tet.), and 1.5 × 10^−10^ (WT + Tet. vs *Trpc6*^−/−^+Tet.+LMWH). Source data are provided as a Source Data file.

Previous reports have shown that cardiac dysfunction is present as early as 2 h after the LPS challenge^20^. To assess which TRPC isoforms are involved in the pathological process resulting in ETM, mice were stimulated with LPS for 4 h and the expressions of TRPC channels protein was measured in the hearts of septic animals. As shown in Fig. 1a, TRPC1 and TRPC6 were the most prominently affected isoforms among the seven TRPC members. A limited time-course study (0–12 h) further demonstrated that the expressions of TRPC1 and TRPC6 protein level peaked at 4 h, in the early phase of ETM onset, before gradually attenuating (Supplementary Fig. 1a), suggesting that TRPC1 and TRPC6 might be involved in the development of LPS-induced cardiac dysfunction. Due to their represented alteration in two subgroups, TRPC1 and TRPC6 were the main focus in a subsequent study.

We further used the *Trpc1* knockout (*Trpc1*^−/−^) and *Trpc6* knockout (*Trpc6*^−/−^) mice to determine their role on the deterioration of the cardiac function in LPS-challenged mice. As shown in Fig. 1b, c, *Trpc* deletion markedly improved the survival of LPS-challenged mice from 0 to 70% (*Trpc1*) or 60% (*Trpc6*) and inhibited the sharp fluctuation of mean arterial blood pressure and heart rate, which was maintained at normal levels in the ETM condition. Echocardiography showed that the severe decreases of ejection fraction in the ETM mice were markedly inhibited by the *Trpc1* and *Trpc6* deletion, indicating that the *Trpc* knockout had significantly improved systolic contractility (Fig. 1d, Supplementary Table 1). Additionally, the myocardial interstitial edema associated with the mononuclear cell infiltration induced by LPS was alleviated in *Trpc1*^−/−^ or *Trpc6*^−/−^ mice (Fig. 1e). The markers of myocardial damage, cardiac troponin-T, troponin-I, and creatine kinase-MB, were significantly decreased in the *Trpc1*^−/−^ or *Trpc6*^−/−^ mice compared to WT mice (Fig. 1f). These data provide evidence that the deletion of *Trpc1* or *Trpc6* markedly protects LPS-induced heart injury in vivo.

### *Trpc1* or *Trpc6* deletion attenuates LPS-triggered intracellular Ca^2+^ release from SR/ER both in isolated mice cardiomyocytes and macrophages

Cardiomyocytes, fibrocytes, and macrophages are significantly involved in endotoxemic cardiac dysfunction^21,22^. Immunolocalization showed that α-sarcomeric actin (cardiomyocyte marker) and CD68 (macrophage marker) co-localized with TRPC1 or TRPC6 positive areas, but DDR2 (fibrocyte marker) didn’t (Supplementary Fig. 1b), indicating that TRPC was expressed in both cardiomyocytes and macrophages. Ca^2+^ release after LPS challenge can trigger vital cardiac signal transduction in the early (hyperdynamic) phase^23^. In both isolated adult mice cardiomyocytes and bone marrow-derived macrophages (BMMs), Ca^2+^ imaging analysis showed that the *Trpc1* or *Trpc6* deletion suppressed the LPS-stimulated [Ca^2+^]_i_ elevation in Ca^2+^-containing extracellular solution (Supplementary Fig. 2a and Supplementary Fig. 3a). Actually, in Ca^2+^-free solution, LPS could elicit a similar transient rise of [Ca^2+^]_i_ (Supplementary Fig. 2a and Supplementary Fig. 3a), suggesting that [Ca^2+^]_i_ increase was mainly attributed to the Ca^2+^ release from SR/ER, instead of extracellular Ca^2+^. Importantly, *Trpc1* or *Trpc6* knockout markedly decreased [Ca^2+^]_i_ to 56.08% or 71.43% (cardiomyocytes) and 53.81% or 62.44% (BMMs) of the WT levels in two types of cells when extracellular Ca^2+^ was removed. Moreover, we also measured [Ca^2+^]_i_ in neonatal mice cardiomyocytes isolated from the WT and *Trpc1* or *Trpc6* knockout mice. There was no significant difference in LPS-triggered intracellular Ca^2+^ release between neonatal and adult mice cardiomyocytes (Supplementary Fig. 2b). Despite evidence supporting that the cardiac-resident macrophages (cMacs) are of myeloid origin^24–26^, to exclude the influence of the different tissue types on cell function, we further prepared cMacs as CD45^+^ CD11b^+^ F4/80^+^ using flow cytometry and cell sorting (Supplementary Fig. 3b). No significant difference was found between cMacs and BMMs in LPS-stimulated [Ca^2+^]_i_ elevation and the effects of *Trpc1* or *Trpc6* deletion on Ca^2+^ leak (Supplementary Fig. 3a, c). Therefore, it is intriguing whether TRPC1 or TRPC6 regulating Ca^2+^ leak from the SR/ER is responsible for the cardiac collapse in the ETM condition.

It has been reported that two IP3R subtypes, IP3R1 (ITPR1) and IP3R2 (ITPR2), and one ryanodine receptor (RyR) subtype, RyR2, are abundant in cardiomyocytes and play important roles in Ca^2+^ leak from the SR^27–29^. To further clarify the essential receptors which regulate Ca^2+^ release in LPS-challenged cardiomyocytes, *Itpr1*, *Itpr2*, and *Ryr2* were knocked down by siRNA in the neonatal mice cardiomyocytes (Supplementary Fig. 2c and e). Calcium imaging experiments showed that *Itpr1* and *Itpr2* siRNA co-transfection decreased LPS-induced [Ca^2+^]_i_ by 80.10%, and *Itpr1* knockdown decreased LPS-triggered [Ca^2+^]_i_ by 56.12% in Ca^2+^-free solution, demonstrating that IP3R1 was the primary receptor in IP3R subtypes involved in the LPS-stimulated intracellular calcium release from SR in cardiomyocytes (Supplementary Fig. 2d). However, although *Ryr2* siRNA slightly inhibited LPS-triggered Ca^2+^ release, there was no significant difference between *Ryr2* siRNA- and control siRNA-transfected neonatal cardiomyocytes (Supplementary Fig. 2f). RyR2 is the primary SR Ca^2+^ release channel in cardiomyocytes, its expression level is typically 50-fold more abundant than IP3Rs in ventricular myocytes^29,30^. Considering the genes’ abundance and gene knockdown efficiency using RNA interference, the *Ryr2* siRNA-transfected cardiomyocytes were further pre-incubated with the RyRs inhibitor, ryanodine, to completely block RyR2. The LPS-induced Ca^2+^ release could be markedly decreased by ryanodine (Supplementary Fig. 2g), indicating that RyRs are also involved in LPS-stimulated intracellular Ca^2+^ release in cardiomyocytes. To clarify the roles of TRPC1 and TRPC6 on RyRs and IP3Rs-regulated Ca^2+^ release trigged by LPS, isolated *Trpc1*^−/−^ and *Trpc6*^−/−^ cardiomyocytes were treated with RyR inhibitors, ryanodine and tetracaine, and/or IP3R inhibitor, low molecular weight heparin (LMWH), respectively. As shown in Fig. 1g, LPS-activated Ca^2+^ release was partially suppressed by ryanodine or tetracaine, and was completely abolished by LMWH both in neonatal and adult mice cardiomyocytes, confirming that RyRs can amplify the IP3R-gated Ca^2+^ releases in mice cardiomyocytes exposed to LPS^31^. Notably, LPS-induced Ca^2+^ releases suppressed by ryanodine or tetracaine were further significantly reduced in *Trpc1* or *Trpc6* knockout cardiomyocytes, indicating that TRPC1 and TRPC6 are essentially involved in the IP3R-gated Ca^2+^ release upon LPS challenge.

Moreover, it has been reported that IP3Rs is responsible for Ca^2+^ release from the ER in macrophages^24^. As predicted, non-specific IP3Rs inhibitor, LMWH could entirely abolish the LPS-triggered [Ca^2+^]_i_ increase in isolated BMMs (Supplementary Fig. 3e). The knockdown of *Itpr1* markedly reduced LPS-induced [Ca^2+^]_i_ increase by 89.29%, further verifying that IP3R1 played the main role in LPS-induced intracellular ER Ca^2+^ release (Supplementary Fig. 3d, e). Since Ca^2+^ release from the SR/ER post-LPS challenge is through IP3Rs, the effect of LMWH on endotoxemic cardiac dysfunction was measured in vivo. However, LMWH did not significantly improve the cardiac function and survival in the ETM mice (Supplementary Fig. 4, Supplementary Table 2), indicating that the blockage of Ca^2+^ release from SR/ER was not sufficient to cure endotoxemic cardiac dysfunction. Thus, the critical mechanism of *Trpc1* or *Trpc6* knockout on cardioprotection seems beyond the inhibition of Ca^2+^ release.

### *Trpc1* or *Trpc6* deletion dramatically attenuates LPS-induced cardiac inflammatory outburst via both the MyD88-dependent and TRIF-dependent pathways

**Fig. 2:**
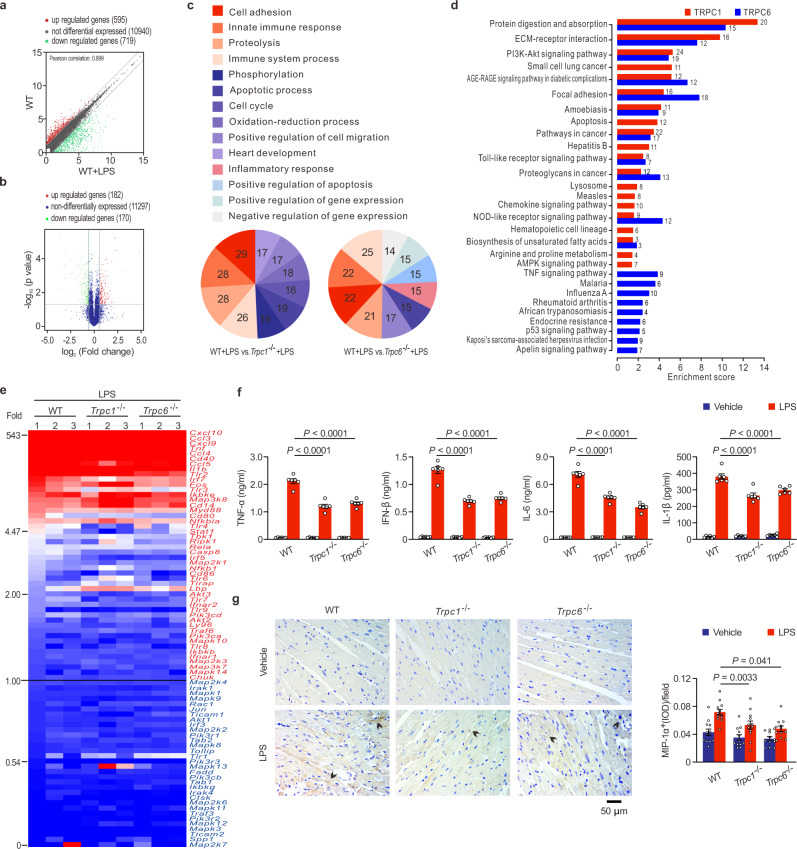
The *Trpc1* or *Trpc6* knockout inhibits TLR signaling pathway. **a** Pearson’s correlation test of all the detected genes of RNA-seq in the LPS-challenged mice compared to control. **b** Volcano plot of the changed genes in RNA-seq between *Trpc1*^−/−^ and *Trpc6*^−/−^ mice after LPS challenge. **c** Counting pie charts depicting the top-ranked biological process classification of the differentially expressed genes in RNA-seq using Gene Ontology terms. **d** The top 20 down-regulated pathways in the Kyoto Encyclopedia of Genes and Genomes (KEGG) database of LPS-challenged *Trpc1*^−/−^ or *Trpc6*^−/−^ mice compared to the WT mice. **e** The heatmap of the genes in the TLR signaling pathway from the KEGG database based on RNA-seq analysis. The values from RNA-seq in **a**–**e** are obtained from 3 male mice per group. **f** Serum levels of TNF-α, IFN-β, IL-6, and IL-1β in mice 6 h after LPS challenge (mean ± SEM, *n* = 6 male mice samples per group). Statistical significance was determined using the one-way ANOVA with Tukey’s multiple comparisons test. TNF-α, exact *P* value = 2.4 × 10^−12^ (WT + LPS vs *Trpc1*^−/−^+LPS), 3.7 × 10^−11^ (WT + LPS vs *Trpc6*^−/−^+LPS); IFN-β, exact *P* value = 9.9 × 10^−13^ (WT + LPS vs *Trpc1*^−/−^+LPS), 3.1 × 10^−12^ (WT + LPS vs *Trpc6*^−/−^+LPS); IL-6, exact *P* value = 5.4 × 10^−11^ (WT + LPS vs *Trpc1*^−/−^+LPS), 8.3 × 10^−13^ (WT + LPS vs *Trpc6*^−/−^+LPS); IL-1β, exact *P* value = 8.0 × 10^−8^ (WT + LPS vs *Trpc1*^−/−^+LPS), 3.4 × 10^−5^ (WT + LPS vs *Trpc6*^−/−^+LPS). **g** Representative photomicrographs (left panel) and quantitative data (right panel) of MIP-1α immunohistochemical staining on ventricular tissues. Arrows show MIP-1α positive cells (mean ± SEM, *n* = 12 images from 4 male mice per group). Statistical significance was determined using the one-way ANOVA with Tukey’s multiple comparisons test. Source data are provided as a Source Data file.

To uncover the key mechanism, quantified RNA-seq was used to systematically assess the changed genes in the endotoxemic hearts of *Trpc1*^−/−^ and *Trpc6*^−/−^ mice (Supplementary Fig. 5). Pearson’s correlation test found 595 strikingly up-regulated and 719 down-regulated genes among 12254 detected genes in ETM mice compared to control (Fig. 2a). In all detected genes, the majority (11297) were not significantly different between the LPS-challenged *Trpc1*^−/−^ and *Trpc6*^−/−^ mice (Fig. 2b). Gene ontology (GO) analyses indicated that the innate immune response was one of the leading changed biological processes in both *Trpc1*^−/−^ and *Trpc6*^−/−^ mice (Fig. 2c). Toll-like receptor (TLR) signaling pathway (ID, mmu04620), which plays an essential role in the innate immune response^32^, was also one of the major changed pathways in the Kyoto Encyclopedia of Genes and Genomes (KEGG) (Fig. 2d). A heatmap demonstrated expression changes about 80 genes of the TLR signaling pathway in the hearts of LPS-challenged WT, *Trpc1*^−/−^, and *Trpc6*^−/−^ mice (Fig. 2e). The markedly changed genes both in the hearts of *Trpc1*^−/−^ and *Trpc6*^−/−^ mice (>1.5-fold or <0.5-fold change) compared with LPS-challenged WT mice, including *Tnfa, Il1b, Il6, CD14, MAPK12, MAPK13, Irf5, Tlr1, Tlr3, Tlr4, Tlr6, Tlr9, Stat1, Cxcl9*, and *Lbp*, were further analyzed using real-time PCR. Most of LPS-stimulated upregulation genes mentioned above were significantly inhibited by the *Trpc1* or *Trpc6* deletion (Supplementary Fig. 6). Moreover, the markers of the TLR signaling pathway activation, key proinflammatory cytokines (TNF-α, IFN-β, IL-1β, and IL-6) in serum (Fig. 2f) and inflammatory protein (MIP-1α/CCL3) in the heart tissues (Fig. 2g) were significantly suppressed by the *Trpc1* or *Trpc6* knockout, demonstrating that the TRPC channels promoted inflammatory responses in the ETM mice.

**Fig. 3:**
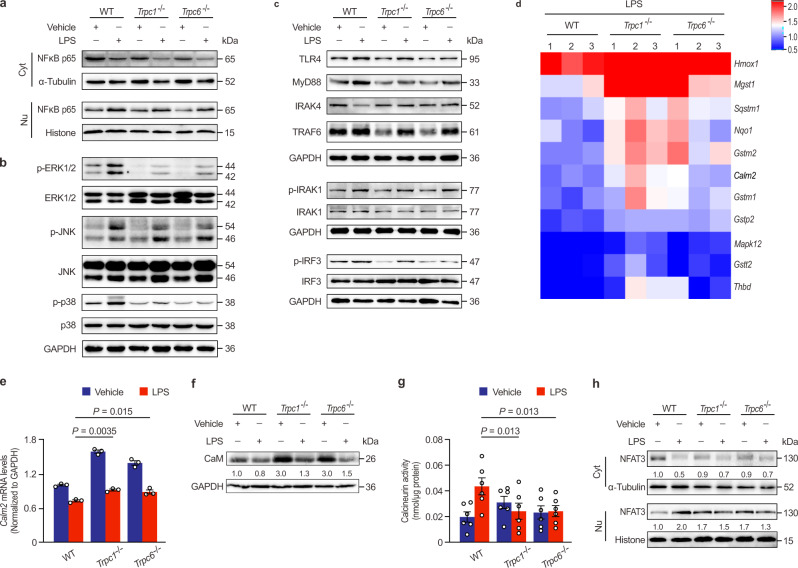
TRPC1 and TRPC6 associate with TLR4 and Ca^2+^ signaling pathways. **a-b** The effects of *Trpc1* or *Trpc6* knockout on nuclear factor-κB (NF-κB) and mitogen-activated protein kinases (MAPK) signaling pathways in the hearts of mice 4 h after LPS challenge (pooled tissues from 3 male mice per sample, *n* = 3 biological independent experiments). **c** The effects of *Trpc1* or *Trpc6* knockout on Toll like receptor 4 (TLR4)‐mediated myeloid differentiation primary response protein 88 (MyD88)- and TIR domain-containing adaptor inducing IFN-β (TRIF)-dependent signaling pathways in the hearts of mice 4 h after LPS challenge (pooled tissues from 3 male mice per sample, *n* = 3 biological independent experiments). **d** Heatmap depicting the genes involved in the Ca^2+^ signaling pathway from the KEGG pathway database based on RNA-seq analysis (*n* = 3 male mice per group). **e**-**f** The effects of *Trpc1* or *Trpc6* deletion on *Calm2* mRNA (n = 3 male mice with triplicate measurements taken, mean ± SEM) and calmodulin (CaM) protein expressions (pooled tissues from 3 male mice per sample, *n* = 3 biological independent experiments) in the hearts of mice 4 h after LPS challenge. **g**-**h**, The activity of calcineurin (mean ± SEM, *n* = 6 male mice samples per group) and NFAT3 nuclear translocation (pooled tissues from 3 male mice per sample, *n* = 3 biological independent experiments) in the hearts of mice 4 h after LPS challenge. Statistical significance was determined using the one-way ANOVA with Tukey’s multiple comparisons test. Source data are provided as a Source Data file.

The translocation of nuclear factor-κB (NF-κB) dimers from the cytoplasm into the nucleus and the activation transcription factor activator protein-1 (AP-1) by mitogen-activated protein kinases (MAPK) are critical pathways downstream of TLR signaling in generating inflammatory genes^33,34^. To confirm these signaling pathways regulated by TRPCs, the expressions of the above key proteins in the hearts of LPS-stimulated mice were measured. The western blotting analysis revealed that the *Trpc1* or *Trpc6* knockout significantly reduced LPS-induced NF-κB p65 nuclear translocation (Fig. 3a) and the phosphorylation of ERK1/2, p38, and JNK, the classical subfamilies of the MAPK (Fig. 3b), suggesting that MAPK and NF-κB were the downstream signaling molecules in the TRPC1- or TRPC6-regulated inflammatory cascades. Upon LPS binding, TLR4 forms a homodimer that recruits two pairs of adaptor proteins, TIR domain-containing adaptor protein (TIRAP) and MyD88, as well as TRIF-related adaptor molecule (TRAM) and TRIF, in the TIR domain involved in the above signaling pathways^35^. The recruitment of TIRAP and MyD88 promotes IL-1R-associated kinase 1/4 (IRAK1/4) and TNF-receptor-associated factor 6 (TRAF6) activation, and then activate MAPK and NF-κB^36^, whereas the recruitment of TRIF and TRAM can further activate IFN regulatory factor 3 (IRF3)^25^. As shown in Fig. 3c, *Trpc1* or *Trpc6* knockout did not cause marked changes in the basal levels of these important proteins in normal hearts, but significantly down-regulated their expressions or phosphorylations in the hearts of LPS-challenged mice, indicating that TRPCs regulated both the MyD88-dependent and TRIF-dependent pathways in endotoxemic hearts.

### Uncoupled CaM from TRPC interacts with TLR4 directly

**Fig. 4:**
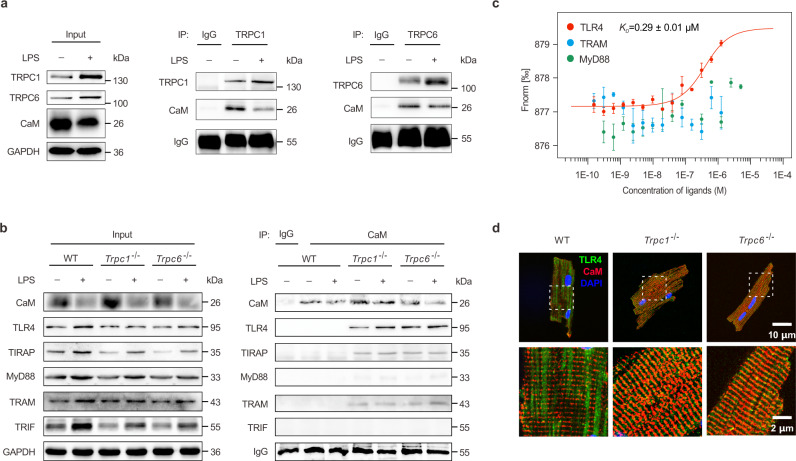
CaM interacts with TLR4 in *Trpc1*^−/−^ or *Trpc6*^−/−^ hearts. **a** Co-immunoprecipitated (Co-IP) analysis of TRPC1 or TRPC6 interaction with CaM in the hearts of mice 4 h after LPS challenge (pooled tissues from 3 male mice per sample, *n* = 2 biological independent experiments). **b** Co-IP analysis of CaM interaction with TLR4 and its adaptor proteins in the hearts of WT, *Trpc1*^−/−^, and *Trpc6*^−/−^ mice 4 h after LPS challenge (pooled tissues from 3 male mice per sample, *n* = 2 biological independent experiments). **c** The protein interactions between recombinant human CALM2 protein and TLR4, MyD88, or TRIF-related adaptor molecule (TRAM) using microscale thermophoresis (MST) assay (mean ± SEM, *n* = 3 biological independent experiments). **d** Immunofluorescence microscopy analysis of CaM and TLR4 in adult mice cardiomyocytes (*n* = 6 images from 3 biological independent experiments). Source data are provided as a Source Data file.

To clarify the mechanism of which TRPC1 or TRPC6 regulates the TLR4 signaling molecules, we further analyzed the RNA-seq genes involved in the Ca^2+^ signaling pathway that is mainly regulated by TRPC (Fig. 3d). *Calm2* as one of the most notable down-regulated genes in the hearts of LPS-challenged WT mice, its mRNA level and its encoding protein CaM expression were markedly enhanced in the endotoxemic *Trpc1*^−/−^ or *Trpc6*^−/−^ hearts (Fig. 3e, f). Consequently, LPS-induced activation of calcineurin, the major CaM-binding protein^37^, was suppressed by *Trpc1* or *Trpc6* knockout (Fig. 3g). It has reported that activated calcineurin can promote NFAT3 to translocate to the nucleus. NFAT3 served as an important transcriptional factor can activate a large number of early response genes, including cytokines, even *Trpc1* and *Trpc6*^38,39^. In the heart tissues, we found that LPS challenge could induce a prominently NFAT3 expression in the nuclei, whereas the endonuclear NFAT3 was remarkably inhibited by the *Trpc1* or *Trpc6* deletion (Fig. 3h). These data revealed that CaM-calcineurin-NFAT3 was mainly involved in the regulation of TRPC1 or TRPC6 in endotoxemic hearts. Furthermore, endogenous CaM was efficiently immunoprecipitated with both TRPC1 and TRPC6 in heart lysis (Fig. 4a). These results confirmed that TRPC1 and TRPC6 interacted with CaM and significantly regulated its essential downstream effectors calcineurin and NFAT3, indicating that CaM was the key protein regulated by TRPCs in endotoxemic hearts.

In particular, using endogenous CaM as bait protein, neither TLR4 nor its adaptor proteins was immunoprecipitated from the same WT heart tissue extracts. However, CaM distinctly co-immunoprecipitated with TLR4, TIRAP, MyD88, and TRAM in heart extracts from *Trpc1*^−/−^ or *Trpc6*^−/−^ mice (Fig. 4b). The Duolink® proximity ligation assay (PLA) was used to verify the co-immunoprecipitation (Co-IP) results at cellular level. CaM:TLR4 PLA gave no detectable punctum in isolated neonatal WT mice cardiomyocytes. However, CaM:TLR4 PLA-produced fluorescent puncta were obviously visible in the *Trpc1* and *Trpc6* knockout cardiomyocytes, further verifying the cooperation of CaM and TLR4 (Supplementary Fig. 7a). At molecular level, the microscale thermophoresis (MST) results further showed recombinant human CALM2 protein binding to TLR4 with dissociation constant (K_D_) value of 0.29 ± 0.01 μM, but not interact with MyD88 or TRAM (Fig. 4c), indicating that endogenous CaM bound to TLR4 in the TLR4:TIRAP:MyD88 and TLR4:TRAM:TRIF complexes. To identify their clear intracellular colocalization at different times post-LPS challenge, neonatal mice cardiomyocytes were examined with markers including PDI, EEA1, and RAB7 for ER, early endosome, and late endosome, respectively (Supplementary Fig. 7b). LPS-challenge increased the TLR4 expression in the perinuclear ER area of WT cells at 1 h and gradually trafficked to the cytomembrane after. Meanwhile, TLR4 was distinctly expressed in the late endosome after 2 h. It is worth noting that CaM was co-located with TLR4 in the late endosome and near cell membrane in the LPS-stimulated *Trpc1*^−/−^ cells. Immunofluorescence analysis for the adult mice cardiomyocytes further confirmed the interaction between CaM and TLR4 in vivo. TLR4, expressed in the cytoskeleton of WT cells, had no colocalization with CaM, whereas in the *Trpc1*^−/−^ or *Trpc6*^−/−^ cells, TLR4 redistributed and colocalized with CaM in the transversal rib-like myofibrils (Fig. 4d).

### CaM binds to TLR4’s Poc site and atypical IQ-like motif to block the inflammation cascades

**Fig. 5:**
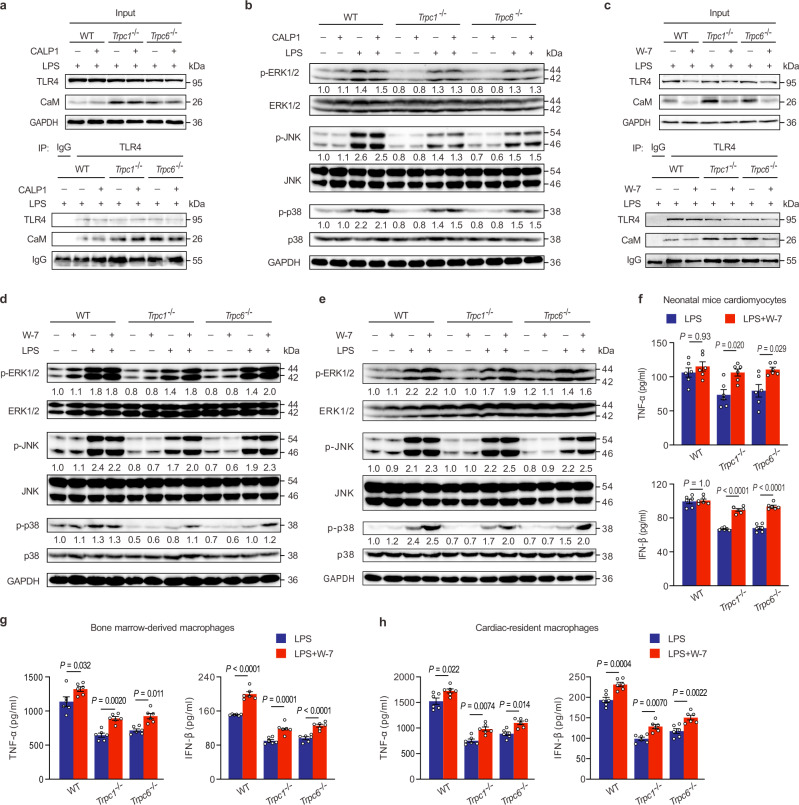
CaM interacts with TLR4 to inhibit the inflammation cascades. **a** The effects of CALP1 (20 μM) pretreatment on the interaction between CaM and TLR4 in the LPS-stimulated neonatal mice cardiomyocytes (*n* = 2 biological independent experiments). **b** The effects of CALP1 on phosphorylated ERK, JNK, and P38 MAPK expressions in the LPS-stimulated neonatal mice cardiomyocytes (*n* = 3 biological independent experiments). **c-d** The effects of CaM inhibitor W-7 (30 μM) pretreatment on the interaction of CaM and TLR4, and MAPK activation in neonatal mice cardiomyocytes stimulated with LPS for 4 h (*n* = 2 biological independent experiments for **c** and *n* = 3 biological independent experiments for **d**). **e** The effects of W-7 pretreatment on MAPK activation in mice bone marrow-derived macrophages stimulated with LPS for 4 h (*n* = 3 biological independent experiments). **f–h** The effects of W-7 pretreatment on TNF-α and IFN-β levels in neonatal mice cardiomyocytes and macrophages stimulated with LPS for 6 h (mean ± SEM, *n* = 6 samples per group). Statistical significance was determined using one-way ANOVA with Tukey’s multiple comparisons test. IFN-β in **f**, exact *P* value = 8.6 × 10^−9^ (*Trpc1*^−/−^+LPS vs *Trpc1*^−/−^+LPS + W-7) and 5.3 × 10^−10^ (*Trpc6*^−/−^+LPS vs *Trpc6*^−/−^+LPS + W-7); IFN-β in **g**, exact *P* value = 6.7 × 10^−9^ (WT + LPS vs WT + LPS + W-7) and 7.5 × 10^-5^ (*Trpc6*^−/−^+LPS vs *Trpc6*^−/−^+LPS + W-7). Source data are provided as a Source Data file.

The Ca^2+^-sensor CaM, composed of N- and C-terminal domains connected with a flexible linker, has two Ca^2+^-binding motifs (termed EF-hands) in each domain^40^. Pretreatment of peptide CALP1, an EF-hands blocker^41^, did not disrupt the interaction between CaM and TLR4 (Fig. 5a); it also couldn’t regulate the phosphorylated MAPK in the LPS-stimulated *Trpc1*^−/−^ or *Trpc6*^−/−^ cardiomyocytes (Fig. 5b). However, pretreatment with the CaM antagonist W-7, which interacts with the deep hydrophobic pocket in CaM and induces a ‘hinge’ region change^40^, uncoupled the CaM binding with TLR4 (Fig. 5c) and markedly increased the phosphorylated MAPK levels in the *Trpc1*^−/−^ or *Trpc6*^−/−^ cardiomyocytes (Fig. 5d) and BMMs (Fig. 5e). Additionally, W-7 treatment reversed the inhibitory effects of the *Trpc1* or *Trpc6* knockout on TNF-α and IFN-β productions in the LPS-stimulated cardiomyocytes (Fig. 5f) and BMMs (Fig. 5g). Since the inhibition of TLR4-mediated inflammation response is a key protection mechanism of *Trpc1* or *Trpc6* knockout against LPS-induced cardiac dysfunction, we further verified the effects of W-7 treatment on LPS-stimulated TNF-α and IFN-β productions in the *Trpc1*^−/−^ or *Trpc6*^−/−^ cMacs. Similarly, *Trpc1* or *Trpc6* knockout significantly suppressed the TNF-α and IFN-β productions, while W-7 treatment reversed these inhibitory effects of the *Trpc1* or *Trpc6* gene deletion in LPS-stimulated cMacs (Fig. 5h). Thus, we speculated that CaM could interact with the vital TLR4 domain in regulating inflammation cascades in endotoxemic hearts, and these interactions might be independent of Ca^2+^-binding.

**Fig. 6:**
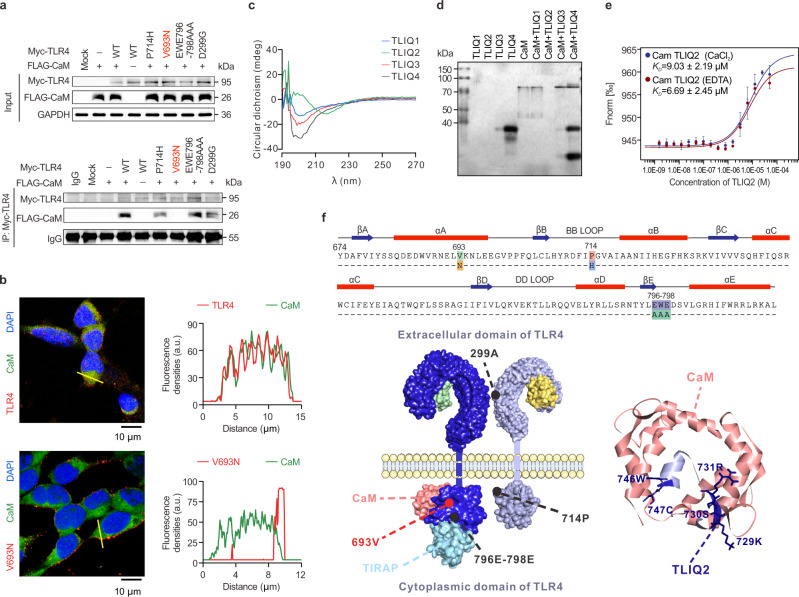
CaM binds to both the Poc site and the atypical IQ motif of TLR4. **a** The interacted domain of TLR4 (Myc-tagged) with CaM (FLAG-tagged) in co-transfected HEK293T cells measured using Co-IP (*n* = 2 biological independent experiments). **b** Representative immunofluorescent photomicrographs (left panel) and traces of fluorescence intensity spatial profiles (right panel) of TLR4 (Myc-tagged) with CaM (FLAG-tagged) localization in co-transfected HEK293T cells (*n* = 6 images from 3 biological independent experiments). **c** The CD spectroscopies of the synthesized peptides. **d** The interaction of CaM with the peptides encompassing nonclassical IQ motifs detected by a non-denaturing gel (*n* = 2 biological independent experiments). **e** The dissociation constants of CaM and peptide TLIQ2 measured using MST (mean ± SEM, *n* = 3 biological independent experiments). **f** Structural model of signaling complex formed by TLR4 and CaM. Secondary structure elements and position of mutational sites in the human TLR4 sequence (upper panel) and a homologous modeling of TLR4 (TLR2, PDB 1Fyx) and TLIQ2 contacting with CaM (PDB 1QX5) (lower panel) are shown. Source data are provided as a Source Data file.

Based on the different binding sites of adaptor proteins, the human TLR4 was mutated in some key residues of the TIR domain, including EWE796-798 (TIRAP interface residues), P714 (TRAM and TIRAP interface residue)^35,42^, and V693 (Poc site, vital for adaptor sensing and dimerization)^35,42,43^, whereas the A299 (interfere TLR4 dimerization) mutant in the extracellular domain was set as the positive control^44^. The Co-IP study showed that CaM failed to bind only the V693N TLR4 among the four mutant loci (Fig. 6a). Confocal microscopy analysis showed that V693N TLR4 was highly expressed at the cell surface and had no distinct colocalization with CaM (Fig. 6b, Supplementary Fig. 8), further confirming that the CaM binding domain in TLR4 contains V693. Moreover, CaM generally interacts with its binding partners that contain an α-helical IQ-like motif, typically 11-amino-acid sequence ([I/L/V]QXXXRGXXX[R/K]), either calcium-dependently or -independently^45^. Through structural prediction using the CaM target database (http://calcium.uhnres.utoronto.ca/ctdb/ctdb/), several nonclassical IQ motifs within the TIR domain of TLR4 demonstrated high affinity for CaM (Supplementary Fig. 9a). We further synthesized four peptides, named TLIQ1, TLIQ2, and TLIQ3 encompassing top-score motifs, and TLIQ4, an V693 overlapping atypical sequence (Supplementary Fig. 9b). Circular dichroism spectroscopies confirmed that these peptides adopt α-helical topology (Fig. 6c). A non-denaturing gel that detects interactions between TLIQs and CaM showed that recombinant human CALM2 protein bound to peptide TLIQ2, resulting in a CALM2-TLIQ2 complex that did not enter the gel (Fig. 6d). Using MST, TLIQ2 also exhibited an affinity for CALM2 with a K_D_ of 6.69 ± 2.45 μM, and the interaction was Ca^2+^-independent, as proven by similar K_D_ values in the absence and presence of Ca^2+^ mediums (Fig. 6e). These results, complemented by data from docking studies, indicated that the hydrophobic pocket (residues 2, 8, 123, 126, 130) of CaM could interact with the atypical IQ motif (residues 729, 730, 731, 746, 747) in αB-αC helix and Poc site of the TLR4’s TIR domain, resulting in blockage of TLR4 signaling pathway (a structural model showed in Fig. 6f).

### The pleiotropic roles of TRPC in regulating CaM and IP3R1

**Fig. 7:**
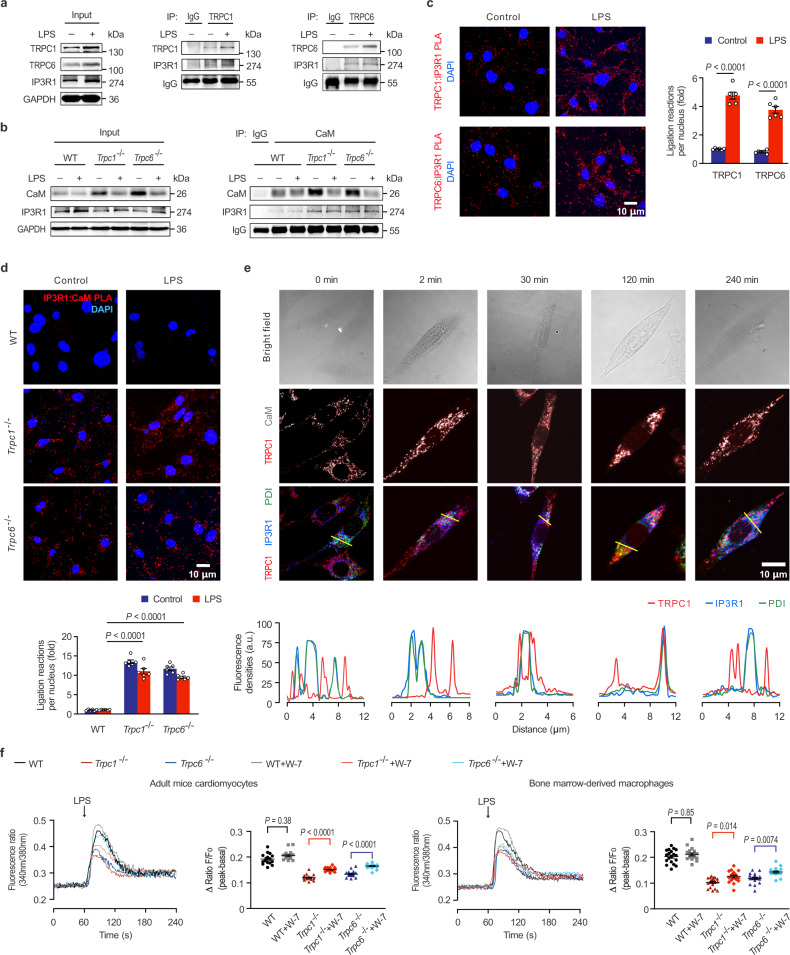
The pleiotropic roles of TRPCs in regulating CaM and IP3R1. **a** The Co-IP analysis of TRPC1 or TRPC6 binding with IP3R1 in mice heart tissues (pooled tissues from 3 male mice per sample, *n* = 2 biological independent experiments). **b** The interaction between CaM and IP3R1 in the hearts of WT, *Trpc1*^−/−^, and *Trpc6*^−/−^ mice 4 h after LPS challenge (pooled tissues from 3 male mice per sample, *n* = 2 biological independent experiments). **c** TRPC1-IP3R1 and TRPC6-IP3R1 interactions in LPS-challenged neonatal WT mice cardiomyocytes. Representative PLA photomicrographs (left panel) and the statistical analysis (right panel) are shown (mean ± SEM, *n* = 6 images from 3 biological independent experiments). Statistical significance was determined using the two-tailed Student’s t-test. Exact *P* value = 2.0 × 10^−5^ (*Trpc1*^−/−^ vs *Trpc1*^−/−^+LPS) and 3.5 × 10^−5^ (*Trpc6*^−/−^ vs *Trpc6*^−/−^+LPS). **d** IP3R1-CaM interactions in LPS-challenged neonatal WT, *Trpc1*^−/−^, and *Trpc6*^−/−^ mice cardiomyocytes. Representative PLA photomicrographs (upper panel) and the statistical analysis (lower panel) are shown (mean ± SEM, *n* = 6 images from 3 biological independent experiments). Statistical significance was determined using the one-way ANOVA with Tukey’s multiple comparisons test. Exact *P* value = 8.3 × 10^−13^ (WT + LPS vs *Trpc1*^−/−^+LPS) and 9.6 × 10^−13^ (WT + LPS vs *Trpc6*^−/−^+LPS). **e** Representative immunofluorescent photomicrographs of TRPC1, CaM, IP3R1, and PDI (upper panel) and traces of fluorescence intensity spatial profiles of IP3R1, TRPC1, and PDI (lower panel) in LPS-stimulated neonatal mice cardiomyocytes (*n* = 6 images from 3 biological independent experiments). **f** The effects of W-7 on the LPS-triggered intracellular Ca^2+^ influx in adult mice cardiomyocytes (left panel) and mice bone marrow-derived macrophages (right panel) are shown (mean ± SEM, *n* = 15–20 cells from 3 male mice per group). Statistical significance was determined using the one-way ANOVA with Game Howell’s multiple comparisons test. In cardiomyocytes, exact *P* value = 8.4 × 10^−10^ (*Trpc1*^−/−^ vs *Trpc1*^−/−^+W-7) and 1.3 × 10^−8^ (*Trpc6*^−/−^ vs *Trpc6*^−/−^+W-7. Source data are provided as a Source Data file.

TRPC channels have the inherent ability to form homomeric and heteromeric channels, playing pleiotropic roles in signal transduction^46,47^. However, Co-IP assay showed that there was no TRPC1 and TRPC6 protein complexes detected in the cardiomyocytes, whether LPS stimulates or not (Supplementary Fig. 10a), suggesting that TRPC1 and TRPC6 play an independent role in endotoxic cardiac dysfunction. The mechanism by which TRPC regulates CaM and calcium signaling after LPS stimulation was further investigated. The cytoplasmic C-terminal of TRPC includes a CaM/IP3R binding (CIRB) domain^48,49^. Among the three IP3R subtypes, IP3R1 was the main and most markedly changed in the mouse heart (Supplementary Fig. 10b). The Co-IP assay further showed that both TRPC1 and TRPC6 bound to IP3R1 (Fig. 7a), and there was no obvious interaction of CaM and IP3R1 in the WT mouse hearts (Fig. 7b). Surprisingly, CaM markedly coupled with IP3R1 in the *Trpc1*^−/−^ or *Trpc6*^−/−^ hearts, irrespective of LPS treatment. PLA further confirmed the interaction between endogenous IP3R1 and TRPC1/TRPC6 in cardiomyocytes (Fig. 7c). It substantially increased near the cell membrane after LPS challenge, suggesting that the LPS facilitates the recruitment of IP3R1 to TRPC1 and TRPC6. Furthermore, CaM: IP3R1 PLA gave no detectable signal in WT cells, whereas produced fluorescent puncta in the cytoplasms of *Trpc1* and *Trpc6* knockout cardiomyocytes (Fig. 7d), which further indicated that CaM could bind with IP3R1 in the *Trpc1*^−/−^ or *Trpc6*^−/−^ cardiomyocytes. Moreover, spatiotemporal changes of TRPC and its regulatory proteins in cardiomyocytes after LPS challenged were detected by confocal microscopy. Under physiological conditions, IP3R1 was expressed in ER detected by fluorescent-labeled PDI and had no distinct colocalization with TRPC1 and CaM (Fig. 7e). Following LPS treatment, TRPC1 co-localized with CaM was gradually increased nearby IP3R1 from 30 min up to 4 h, suggesting that TRPC and CaM were both involved in the Ca^2+^ release via IP3R1. These data indicate that IP3R1 has a higher affinity with TRPC than CaM; hence, CaM interacts with IP3R1 only during *Trpc* deficiency.

The effect of W-7 on the LPS-induced release of Ca^2+^ from ER was analyzed in isolated cardiomyocytes and macrophages to confirm the above findings. As expected, preincubation with W-7 could not change the LPS-stimulated [Ca^2+^]_i_ in the WT cells (Fig. 7f). However, W-7 notably overturned the *Trpc1* or *Trpc6* knockout-suppressed [Ca^2+^]_i_, indicating that uncoupled CaM can inhibit the LPS-stimulated Ca^2+^ release from ER. Collectively, these results provide evidence that TRPC is critical in contributing to Ca^2+^ release from ER by interacting with IP3R1; in addition, CaM can block IP3R1 to inhibit Ca^2+^ leakage when TRPC is suppressed.

### TRPC blockade cures cardiac dysfunction induced by LPS or cecal ligation and puncture (CLP)

**Fig. 8:**
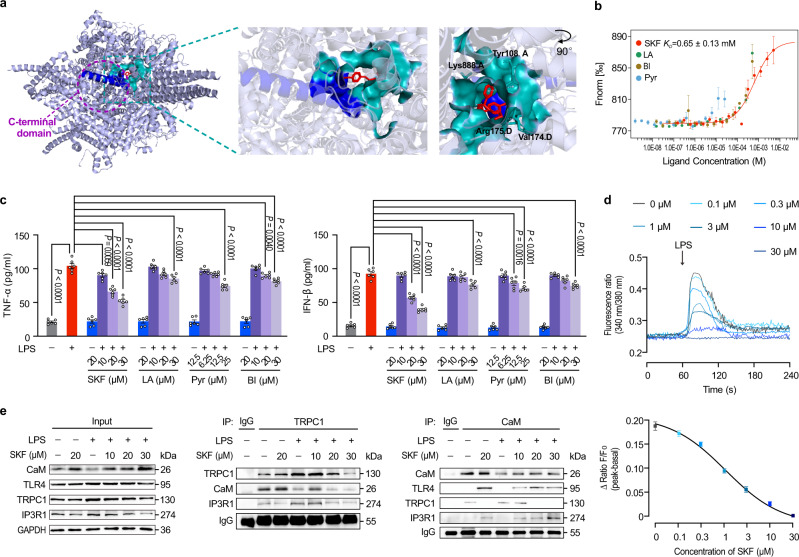
SKF96365 (SKF) blocks TRPC to obstruct the Ca^2+^ release and TLR4-mediated inflammation burst. **a** Structural insights into the SKF binding pocket in human TRPC6 (PDB code 5YX9) and its interaction with the CIRB domain of TRPC6. **b** The interaction between the C-terminal TRPC1 fusion protein and potential inhibitors, SKF, Larixyl acetate (LA), Pyr10 (Pyr), and BI-749327 (BI), measured by MST assay (mean ± SEM, *n* = 3 biological independent experiments). **c** The effects of TRPC inhibitors on TNF-α and IFN-β productions in LPS-stimulated neonatal mice cardiomyocytes (mean ± SEM, *n* = 6 samples per group). Statistical significance was determined using the one-way ANOVA with Tukey’s multiple comparisons test. TNF-α, exact *P* value = 4.1 × 10^−12^ (WT vs WT + LPS), 4.1 × 10^−12^ (WT + LPS vs LPS + SKF 20 μM), 4.1 × 10^−12^ (WT + LPS vs LPS + SKF 30 μM), 1.4 × 10^−5^ (WT + LPS vs LPS + LA 30 μM), 7.5 × 10^−12^ (WT + LPS vs LPS + Pyr 25 μM), and 8.0 × 10^−8^ (WT + LPS vs LPS + BI 30 μM); IFN-β, exact *P* value = 4.1 × 10^−12^ (WT vs WT + LPS), 4.1 × 10^−12^ (WT + LPS vs LPS + SKF 20 μM), 4.1 × 10^−12^ (WT + LPS vs LPS + SKF 30 μM), 6.7 × 10^−5^ (WT + LPS vs LPS + LA 30 μM), 3.1 × 10^−8^ (WT + LPS vs LPS + Pyr 25 μM), and 8.0 × 10^−6^ (WT + LPS vs LPS + BI 30 μM). **d** SKF inhibits the LPS-triggered intracellular Ca^2+^ release in adult mice cardiomyocytes in Ca^2+^-containing extracellular solution (mean ± SEM, *n* = 15–20 cells from 3 male mice per group). **e** The effects of SKF on the interactions between TRPC1 and CaM/IP3R1 and between CaM and TLR4/IP3R1 in LPS-challenged neonatal mice cardiomyocytes (*n* = 2 biological independent experiments). Source data are provided as a Source Data file.

Based on the above study, the effect of chemically inhibiting the critical CIRB domain in TRPC on septic cardiac dysfunction was further evaluated. Since the C-terminal residues are conserved in all TRPC members, it is possible to completely block TRPCs’ function in septic cardiac dysfunction by a chemical inhibitor of the CIRB domain. Therefore, 23 available TRPC antagonists known for interacting with human TRPC6 and TRPC3 proteins were analyzed using Sybyl molecular modeling software (Supplementary Table 5). The nonspecific blocker SKF96365 (SKF) showed a predicted high binding capacity and putative interaction sites in the CIRB domain (Fig. 8a). To identify these blockers interacting with the CIRB domain, we expressed the C-terminal TRPC1 fragment containing the CIRB domain as fusion protein and purified from *Escherichia coli*. SKF could bind to the TRPC1 fusion protein with the K_D_ value of 0.65 ± 0.13 mM using MST among four TRPC antagonists, SKF, Larixyl acetate, Pyr10, and BI-749327, which ranked top scores in binding energy of molecular modeling and were commercially available (Fig. 8b). Furthermore, these blockers all concentration-dependently decreased the production of TNF-α and IFN-β in LPS-treated cardiomyocytes, which was consistent with the results of virtual screening except for Pyr10 due to its poor solubility (Fig. 8c). SKF showed the highest inhibitory effects at 30 μM among these blockers and was used in the subsequent study. At the cellular level and in a concentration-dependent manner, SKF could inhibit high expressed TRPC subtypes (TRPC1, TRPC3, and TRPC6) (Supplementary Fig. 10c), decreased the LPS-induced Ca^2+^ release in Ca^2+^-containing extracellular solution in cardiomyocytes (Fig. 8d). Intracellular Ca^2+^ concentration in 30 μM SKF-treated cells was even almost to the normal level. Furthermore, SKF could inhibit the LPS-stimulated TLR4 and IP3R1 expressions in a concentration-dependent manner, while increased the CaM expression in cardiomyocytes (Fig. 8e). Co-IP assay demonstrated that the binding between TRPC1 and CaM was disrupted by SKF in a concentration-dependent manner, regardless of LPS; meanwhile, the LPS-stimulated interaction of TRPC1 and IP3R1 was distinctly inhibited by SKF. Correspondingly, SKF treatment markedly increased the binding between CaM and TLR4 or IP3R1 after LPS stimulation.

**Fig. 9:**
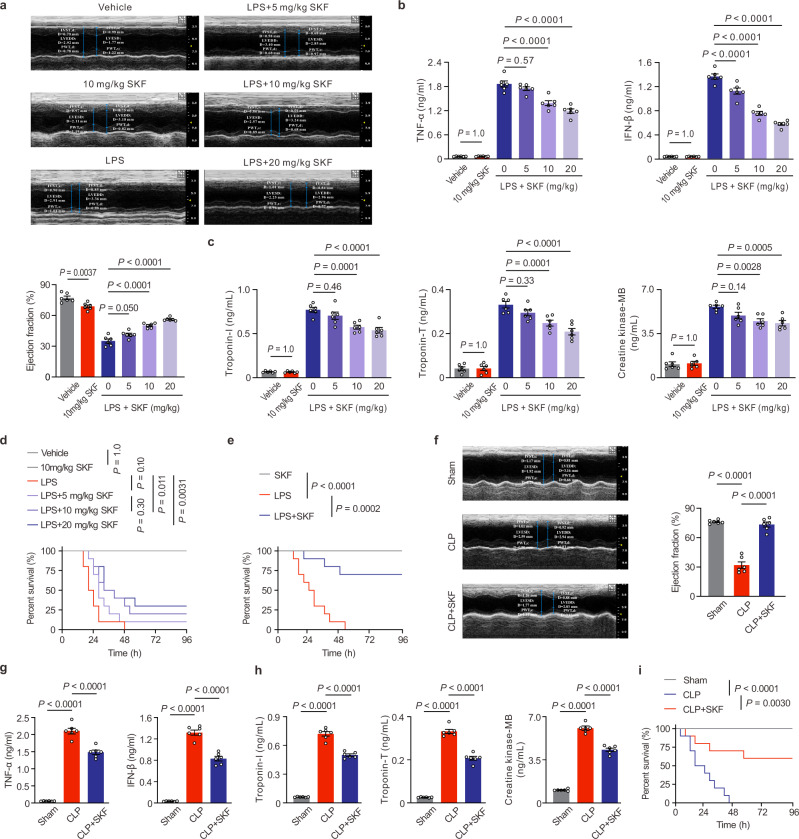
SKF cures septic cardiac dysfunction. **a** Representative M-mode echocardiography still and the statistical analysis of ejection fraction in vehicle- or SKF-treated mice at 6 h after LPS challenge (mean ± SEM, *n* = 6 male mice per group). Statistical significance was determined using the one-way ANOVA with Tukey’s multiple comparisons test. Exact *P* value = 4.5 × 10^−7^ (LPS vs LPS + SKF 10 mg/kg) and 2.9 × 10^−10^ (LPS vs LPS + SKF 20 mg/kg). **b** SKF treatment on the serum levels of TNF-α and IFN-β in LPS-challenged mice (mean ± SEM, *n* = 6 male mice samples per group). Statistical significance was determined using the one-way ANOVA with Tukey’s multiple comparisons test. TNF-α, exact *P* value = 2.0 × 10^−6^ (LPS vs LPS + SKF 10 mg/kg) and 1.2 × 10^−9^ (LPS vs LPS + SKF 20 mg/kg); IFN-β, exact *P* value = 8.7 × 10^−5^ (LPS vs LPS + SKF 5 mg/kg), 1.0 × 10^−12^ (LPS vs LPS + SKF 10 mg/kg), and 8.3 × 10^−13^ (LPS vs LPS + SKF 20 mg/kg). **c** SKF treatment on the serum markers of myocardial damage in LPS-challenged mice (mean ± SEM, *n* = 6 male mice samples per group). Statistical significance was determined using the one-way ANOVA with Tukey’s multiple comparisons test. Troponin-I, exact *P* value = 7.0 × 10^−6^ (LPS vs LPS + SKF 20 mg/kg); Troponin-T, exact *P* value = 1.0 × 10^−6^ (LPS vs LPS + SKF 20 mg/kg). **d** Kaplan-Meier survival curves of single-dose SKF treatment (*n* = 10 male mice per group). **e** Survival curves of 10 mg/kg SKF multiple-dosing (every 12 h, *n* = 10 male mice per group). Statistical significances in **d** and **e** were determined using the log-rank test. Exact *P* value = 3.0 × 10^−6^ (SKF vs LPS). **f** Echocardiographic assessment of left ventricular function of SKF-treated mice at 6 h after CLP surgery. Typical heart M-mode echocardiography still (left), ejection fraction (right panel) are shown (mean ± SEM, *n* = 6 male mice per group). Statistical significance was determined using the one-way ANOVA with Game Howell’s multiple comparisons test. Exact *P* value = 5.7 × 10^−5^ (WT vs CLP) and 7.0 × 10^−6^ (CLP vs CLP + SKF). **g** The serum levels of TNF-α and IFN-β in the SKF-treated CLP mice (mean ± SEM, *n* = 6 male mice samples per group). Statistical significance was determined using the one-way ANOVA with Tukey’s multiple comparisons test. TNF-α, exact *P* value = 5.8 × 10^−9^ (WT vs CLP) and 1.2 × 10^−5^ (CLP vs CLP + SKF); IFN-β, exact *P* value = 5.8 × 10^−9^ (WT vs CLP) and 2.3 × 10^−7^ (CLP vs CLP + SKF). **h** SKF treatment on the serum markers of myocardial damage in CLP mice (mean ± SEM, *n* = 6 male mice samples per group). Statistical significance was determined using the one-way ANOVA with Tukey’s multiple comparisons test. Troponin-I, exact *P* value = 5.8 × 10^−9^ (WT vs CLP) and 1.3 × 10^−7^ (CLP vs CLP + SKF); Troponin-T, exact *P* value = 5.8 × 10^−9^ (WT vs CLP) and 2.5 × 10^−8^ (CLP vs CLP + SKF); Creatine kinase-MB, exact *P* value = 5.8 × 10^−9^ (WT vs CLP) and 2.2 × 10^−7^ (CLP vs CLP + SKF). **i** Kaplan-Meier survival curves of multiple-dosing SKF treatment on CLP mice (*n* = 10 male mice per group). Statistical significance was determined using the log-rank test. Exact *P* value = 3.0 × 10^−6^ (WT vs CLP). Source data are provided as a Source Data file.

In vivo, echocardiography assay showed that cardiac function disrupted by LPS in mice was dose-dependently alleviated by single-dosing SKF treatment (Fig. 9a, Supplementary Table 3). In 5 mg/kg, 10 mg/kg, and 20 mg/kg SKF-treated LPS groups, ejection fraction increased by 17.70%, 42.92%, and 60.24%, compared with LPS group. Furthermore, at 6 h after LPS challenge, cardiac histopathological examination revealed the pathological changes, such as increased interstitial edema, cytoplasmic vacuolation, and leukocytic infiltration were obviously improved by SKF dose-dependently (Supplementary Fig. 11a). The serum inflammatory factors TNF-α and IFN-β, and myocardial damage markers including cardiac troponin-T, troponin-I, and creatine kinase-MB induced by LPS were significantly decreased by SKF compared to model mice (Fig. 9b, c). As for mortality, we found that SKF could also dose-dependently prolong the survival of LPS-challenged mice (Fig. 9d). The survival rates of 5, 10, and 20 mg/kg SKF-treated mice challenged with LPS were 10%, 20%, and 30%, with no marked difference in 5 and 10 mg/kg SKF-treated groups compared with LPS-challenged group. The clearance half-life of LPS could be up to 15 h in the circulation of mice^50–52^, which might exceed that of SKF and lead to single-dosing SKF treatment inadequate to antagonize TRPC in the whole LPS effective period. Taking into account the metabolism and elimination of the drug, the LPS-challenged mice were further multiple injected (every 12 h) with 10 mg/kg SKF. The survival rates of SKF-multiple-dosing treated ETM mice were 70%, so SKF dramatic prolong the survival time of ETM mice (*P* < 0.0001) (Fig. 9e).

To mimic the clinical scenario of polymicrobial sepsis, the mice that had CLP surgery were also treated with 10 mg/kg SKF; the similarity was striking. The echocardiography assay demonstrated that ejection fraction in the CLP group was significantly lower than that in the sham group, while markedly higher in SKF-treated CLP mice (Fig. 9f, Supplementary Table 4), confirming that TRPC chemical inhibitor protects the heart during severe sepsis in mice. Also, the cardiac pathological changes and the markers of serum inflammation and myocardial damage in SKF-treated group were markedly decreased in comparison with those in the model group at 6 h post-severe CLP surgery (Supplementary Fig. 11b, Fig. 9g, h), suggesting that the inhibitor could improve immune dysregulation during sepsis. In addition, we further assessed SKF on the survival of CLP mice. Results indicated that the CLP mice treated SKF following by CLP surgery had significantly improved 3-day survival compared to septic mice treated with vehicle control; survival rates were improved from 0 to 60% after the medium-dose SKF treatment (Fig. 9i). Taken together, these data clearly corroborates that the TRPCs inhibition can effectively protect against LPS- and CLP- induced cardiac dysfunction. Moreover, we identify TRPC channels as the key mediator of endotoxemic cardiac dysfunction via regulating the inflammation cascade and the Ca^2+^ influx from ER.

## Discussion

Apart from their classical ion channel function responsible for inward Ca^2+^ flow, TRPC channels synergize cytosolic calcium and TLR responses in endotoxemic cardiac dysfunction. We corroborated that TRPCs deletion or blockade could abolish cardiac collapse in ETM through hampering the LPS-induced intracellular Ca^2+^ leak via IP3R from ER and freeing CaM to bind with TLR4, which inhibits the MyD88- and TRIF-mediated signaling pathways (Supplementary Fig. 12). TRPC is the core in orchestrating [Ca^2+^]_i_ and inflammation cascades in endotoxemic hearts. Most importantly, targeting TRPC as a novel therapy for cardiac dysfunction and inflammation could be obtained by small molecule inhibitors in experimental sepsis.

Septic patients with myocardial dysfunction have a 3-fold increase in mortality compared with patients without cardiovascular injury^6^. Thus, the myocardial function seems to be a decisive factor in maintaining survival in ETM. In the literature, TRPC channels have been proven to be linked to the pathogenesis of cardiac diseases, including essential hypertension, cardiac hypertrophy, and heart failure^15,38^. Ca^2+^ as a vital second messenger orchestrates a variety of cellular functions such as muscle contraction and cytoskeleton dynamics^53–55^. Within the heart, calcium conductance via these non-voltage TRPC channels plays an important role in maintaining the balance of Ca^2+^ flux in excitation-contraction coupling and may contribute to aforementioned cardiovascular diseases^56^. However, the pathophysiology of ETM consists of early (hyperdynamic) and late (hypodynamic) phases; its myocardial dysfunction presents as contractile defects characterized by fluctuated mean arterial blood pressure and heart rate and diminished ejection fraction^57^. Increased Ca^2+^ leakage from the ER of endotoxemic animals contributes to the hyperdynamic phase^18^. Proinflammatory cytokines outburst mainly from the activation of the TLR4 signaling pathway decreases [Ca^2+^]_i_ and Ca^2+^ transient resulting in late depressed cardiomyocyte contractility and fatal cardiac arrhythmias^12^. Indeed, LPS stimulation sharply increased the mean arterial blood pressure and heart rate at 1–2 h and inhibited them since then. Meanwhile excessive activation of inflammation aggravates cardiac dysfunction and this “hyper-inflammatory” phase covers approximately the first 24 h before the upregulation of anti-inflammatory mechanisms^58^. In the present study, both *Trpc1*- and *Trpc6*-deficient mice showed down-regulated the mean arterial blood pressure and heart rate in early phase, and strikingly enhanced cardiac output in late phase after LPS stimulation, compared with WT mice (Fig. 1c, d). Moreover, our results indicated that the above pleiotropic signs of ETM, including increased Ca^2+^ leakage from the ER and proinflammatory cytokines outburst were markedly reverted in the *Trpc1*^−/−^ or *Trpc6*^−/−^ mice.

The major sources of intracellular Ca^2+^ are the extracellular space and the intracellular Ca^2+^ stores (e.g. vacuole and SR/ER). The L-type calcium channel is essential for most of the plateau current of the action potential and myocardial contractility under physiological conditions. Studies have shown that the primary mechanism for Ca^2+^ release in the early phase of LPS administration is through the IP3R localized on the ER^12,18^, although the generation of diacylglycerol induced by LPS can activate extracellular Ca^2+^ entry via TRPC6 in endothelial cells^21^. However, cardiomyocytes, as the excitable cells, express the RyRs in SR and possess all three IP3R isoforms^59–62^. RyRs triggered by the Ca^2+^ influx through the voltage-operated L-Type Ca^2+^ channels (LTCC), can elicit local Ca^2+^ release events, which are the basis for global Ca^2+^ transients called Ca^2+^-induced Ca^2+^ release (CICR)^62^. Actually, except that Ca^2+^ enters the cytosol from the extracellular space via LTCC, Ca^2+^ release from the SR, such as IP3R-gated Ca^2+^ release, can also elicit Ca^2+^ release through RyRs clusters in cardiomyocytes^31,63,64^. Previous studies have demonstrated that SR Ca^2+^ release induced by IP3 through IP3Rs can be amplified by CICR via RyR2 in atrial myocytes^63,65,66^. In this study, we verified that the important contribution of RyRs to the raising of IP3R-gated Ca^2+^ release upon LPS challenge in mice cardiomyocytes (Fig. 1g). As for nonexcitable macrophages, IP3Rs are responsible for releasing Ca^2+^ from the ER and forming functional Ca^2+^ selective ligand-gated channels to modulate [Ca^2+^]_i_^24,67^. It has reported that LPS could elevate Ca^2+^ levels in RAW 264.7 murine macrophage cell lines and BMMs, whereas, in the presence of 2-aminoethoxydiphenyl borate, an inhibitor of IP3Rs, [Ca^2+^]_i_ elevation induced by LPS was significantly reduced^25,26^. Our results demonstrated that *Itpr1* knockdown markedly reduced LPS-induced [Ca^2+^]_i_ increase by 89.29%, showing the vital role in LPS-induced Ca^2+^ release in BMMs (Supplementary Fig. 3d, e). Furthermore, IP3R inhibitor LMWH, which blocks the IP3-binding site^68^, can completely inhibit LPS-induced [Ca^2+^]_i_ elevation both in cardiomyocytes and macrophages (Fig. 1g and Supplementary Fig. 3e). Hence, abnormal depletion of SR/ER Ca^2+^ stores is a critical initiating step in endotoxemic cardiomyopathy. It has reported that *TRPC3* knockdown abolished the stimulatory effect of IP3 in airway smooth muscle cells^68^, suggesting that TRPC channels directly correlate with IP3R in the depletion of Ca^2+^ in the SR. Our results showed that TRPC1 or TRPC6 could interact with IP3R1 and their knockout significantly inhibited ER/SR Ca^2+^ release in LPS-challenged cardiomyocytes and macrophages (Fig. 7). Moreover, TRPCs inhibitor could completely inhibit LPS-triggered SR Ca^2+^ release in mice cardiomyocytes (Fig. 8d). These results indicated that TRPC channels, at least TRPC1 and TRPC6, play pivotal roles in the Ca^2+^ mobilization from the SR/ER in the early acute phase of endotoxemic cardiac dysfunction. Depletion of SR/ER Ca^2+^ stores normally triggers a conformational change in the stromal interaction molecule 1 and interacts with calcium release-activated calcium modulator 1 (CRACM1/Orai1) to refill the intracellular Ca^2+^ stores, known as SOCE, resulting in more sustained Ca^2+^ signals^69^. However, evidence from the literature shows that LPS can significantly inhibit Orai1 expression and decrease contractile responses to Ca^2+^ in resistant mesenteric arteries^70^. Meanwhile, the breakout of proinflammatory cytokines, such as TNF-α, limits the re-establishment of Ca^2+^ stores^71^.

As for the late phase, LPS can directly mediate TLR4 in cardiomyocyte and macrophage to activate the synthesis of TNF-α, IL-1β, etc, which subsequently causes fatal myocyte contractility impairment^9^. For example, TNF-α induces inducible nitric oxide synthase to generate high levels of NO, which impairs contractile function through the reduction in myofilament calcium responsiveness^72–74^. Moreover, TNF-α increases the formation of sphingosine, which depresses inotropic activity by inhibiting L-type calcium current, leading to contractile dysfunction^75^. Meanwhile, LPS could induce calcium-dependent activation of calcineurin to dephosphorylate NFAT. After nuclear translocation, transcription factor NFAT directly interacts with NF-κB, and then synergistically promotes their transcriptional activations to mediate maximal production of pro-inflammatory cytokines and chemokines in cardiomyocytes, resulting in aggravated inflammatory responses and cardiac dysfunction^76^. It has been reported that TRPC channels mediate immunological functions and inflammatory responses in T-helper cells, B cells, endothelial cells, etc, through regulating extracellular Ca^2+^ entry or SOCE^21,77,78^. However, the complete loss of SOCE by a deficiency in stromal interaction molecule 1 does not affect inflammatory response to LPS, even elevates systemic cytokine levels^79^. Our study demonstrated that the production of TNF-α, IL-1β, IL-6, and IFN-γ was markedly inhibited both in *Trpc1*^−/−^ and *Trpc6*^−/−^ mice at 6 h post-LPS challenge (Fig. 2f), indicating that, different from sole [Ca^2+^]_i_ regulation, deletion of *Trpc1* or *Trpc6* displayed a more advanced blockage of the LPS-induced inflammatory outburst. To this day, there is limited evidence that the tissue-protective effect of TRPC channels is mediated by the inflammatory signaling pathway. TLR4 is type I transmembrane receptor, which first encounters LPS in the extracellular space, and rapidly induces the assembly of intracellular adaptor proteins^80^. Upon LPS binding, TLR4 oligomerizes and recruits adaptor proteins through homophilic interactions between TIR domains in the cytoplasmic tail of TLR4 and those present on the adaptors^35^. In this study, we found that TRPCs deletion could uncouple CaM, serving as a scaffold, to block the TLR4 interaction with its intracellular adaptor proteins and then inhibit inflammation after LPS exposure (Figs. 4–5). Using Co-IP and immunofluorescence, we found that V693N TLR4 did not bind to CaM, whereas WT CaM interacted with TLR4 (Fig. 6a, b). CaM-binding proteins do not share a strong sequence homology. Nonetheless, many of them often possess a region that is characterized by an α helix consisting the consensus sequence IQXXXRGXXXXR, known as the IQ motif^81^. IQ motifs binds to CaM in Ca^2+^ free or holo state inducing its conformational change, and resulting in modulation of complex signaling, such as activation of the Ras/MAPK pathway^82^. Our current study found a novel nonclassical IQ motif with high affinity for CaM in Ca^2+^ free state by MST and non-denaturing gel assays, indicating that CaM also binds to αB-αC helix of TIR domain (Fig. 6d, e).

Although CaM represents a pivotal endogenous molecular regulated by TRPCs to inhibit inflammatory responses, a sole agonist or mimetic peptide treatment might damage the LPS-stimulated heart compared with *Trpc* knockout or inhibition. CaM as one of the most important intracellular Ca^2+^ receptors could interact with many intracellular proteins, including contractile apparatus, phosphatases, kinases, transcription factors, and histone deacetylase to participate in various intracellular signaling pathways^83^. In the heart, it’s well established that the Ca^2+^/CaM binding activates a catalytic A-subunit of calcineurin and then triggers the upregulation of *Trpc6* expression via the NFAT binding sites in the *Trpc6* promoter to further deteriorate cellular Ca^2+^ events^38,55^. The present study further verified that *Trpc1* or *Trpc6* knockout markedly inhibited calcineurin activity due to blocking the LPS-elicited [Ca^2+^]_i_ increase, and attenuated the endonuclear NFAT expression (Fig. 3g, h). In light of this, TRPCs rather than CaM could become a novel target for drug development against cardiac dysfunction, even multiple organ dysfunctions such as lung and kidney in ETM. Since the C-terminal CIRB domain in TRPC proteins is the critical binding site for IP3R and CaM to regulate [Ca^2+^]_i_ and inflammation cascades, respectively, the identification of novel inhibitors blocking the CIRB domain may provide the new avenue to control the progression of ETM-induced cardiac dysfunction. The chemical blocker SKF, identified by virtual screening and in vitro testing according to the CIRB domain structure (STable 5, Fig. 8), showed powerful cardioprotective effects and markedly reduced mortality from endotoxemia and polymicrobial sepsis (Fig. 9). However, the affinity of SKF for C-terminal TRPC1 fusion protein is not very high and it has reported that SKF exacerbated aortic injury in aortic medial degeneration by modulating the expression of contractile proteins^84^, suggesting that SKF could have side effects. Nevertheless, our results provided evidence that C-terminal TRPCs blockers can be used in treating endotoxemic cardiac dysfunction.

Besides TRPC1 and TRPC6, TRPC3 was relatively abundant in the murine myocardium (Fig. 1a). Evidence supports that cardiac contractility is associated with the TRPC3-mediated Ca^2+^ influx in cardiomyocytes; TRPC3 knockout mice showed protection from phenylephrine-induced pathologic cardiac hypertrophy traced to the modulated expression of CaV1.2 and the decreased Ca^2+^ influx^85^. TRPC3 overexpression increased the sensitivity of cardiomyocytes to apoptosis following ischemia/reperfusion due to the increase in calpain-mediated proteolysis and Ca^2+^ overload^86^. Our data showed that SKF treatment decreased the LPS-stimulated expression of TRPC3 in cardiomyocytes (Supplementary Fig. 10c), implying that TRPC3 contributes to the improvement of endotoxemic cardiac dysfunction by SKF. However, by contrast to TRPC6, there was little to no expression of TRPC3 in the native monocyte-macrophage lineage^87^. Hence, TRPC1 and TRPC6 are the prominent changed TRPC isoforms in the hearts of LPS-challenged mice. In addition, *Trpc1* and *Trpc6* knockout mice exhibit developmental differences compared with WT mice. *Trpc1*^−/−^ mice show impairment in spatial working memory and fear memory formation^88,89^, defects in exocrine glands with reduced salivary fluid secretion^90^, a modest decrease in osteoblastogenesis, and increased bone mass^91^. The loss of TRPC6 changes the structure of the placenta and reduces litter sizes^92^. Although there is no clear report on the presence of identified gene that is carried along, *Trpc6*^−/−^ mice exhibit an elevated blood pressure due to the upregulation of constitutively active TRPC3 channels^93^. Further in-depth investigation will be required to assess the target specificity, potency, gender and species differences, and toxicological safety of novel C-terminal TRPCs blockers.

Our study is a pioneer in providing evidence that TRPC1 and TRPC6 promote endotoxemic cardiac dysfunction. Both *Trpc1*^−/−^ and *Trpc6*^−/−^ mice exhibited higher survival and strikingly enhanced cardiac output compared with WT mice after the LPS challenge. The observed beneficial effects of *Trpc1* or *Trpc6* knockout were associated with the inhibition of Ca^2+^ influx from ER in both cardiomyocytes and macrophages and the blockage of the NF-κB, MAPK, and IRF3 pro-inflammatory pathways. TRPC’s molecular partner, calmodulin is uncoupled during *Trpc1* or *Trpc6* deficiency and binds to TLR4’s Poc site and atypical IQ-like motif to block these inflammation cascades. The C-terminal CIRB domain in TRPC proteins is the critical binding loci for IP3R and CaM to regulate Ca^2+^ influx and inflammation cascades, respectively. Even more, the chemical blocker SKF, binding to the CIRB domain of TRPCs, showed powerful cardioprotective effects and markedly reduced mortality from ETM and polymicrobial sepsis. Hence, targeting TRPC as a novel therapeutic strategy for cardiac dysfunction in experimental sepsis could be implemented by small molecule inhibitors.

## Methods

### Mice

129S-TRPC1-KO (Stock # 37347-JAX) and 129S-C57BL/6-TRPC6-KO (Stock # 37345-JAX) mouse strains were purchased from the Jackson Laboratory (Bar Harbor, ME, USA). C57BL/6 mice were purchased from the Laboratory Animal Center of Fourth Military Medical University (FMMU). The *Trpc1*^−/−^ and *Trpc6*^−/−^ mice were backcrossed with C57BL/6J mice for more than 10 generations. The WT mice derived from identical C57BL/6J genetic background with two KO mouse lines were used as the control group. All mice were maintained at FMMU. Animal care and handling were performed in accordance with the recommendations in the Guide for the Care and Use of Laboratory Animals of the National Institutes of Health. The experimental protocol was approved by the Committee on the Ethics of Animal Experiments of the FMMU (XJYYLL-2014484). The mice used in the study had the following housing conditions: humidity, 50–60%; temperature, 22–24 °C; dark/light cycle, 12 h dark/12 h light.

### LPS-induced ETM model

LPS (#L3024, *Escherichia coli*, O111:B4) was purchased from Sigma-Aldrich, which was purified using ion-exchange chromatography; The purity was >98% containing <1% protein and <1% RNA. ETM was induced as previously described^94^. Briefly, male mice (2 months old) were challenged with either LPS (50 mg/kg) or saline vehicle intraperitoneal (i.p.) injection. In vivo cardiac function was assessed by echocardiography at 6 h post-injection. Additionally, mice were anesthetized by i.p. injection with 70 mg/kg sodium pentobarbital at different time points post-LPS challenge as indicated in the figure legends. The blood samples drawn from the orbits of the mice were used for ELISA assay or other biochemical analysis. The hearts (ventricle) were removed and used for pathological analysis, western blotting, or other assays.

### Survival study

LPS (50 mg/kg) was injected (i.p.) into male mice (*n* = 10/group). SFK or LMWH was administered by tail vein injection. For SKF single-dosing study, C57BL/6 J mice were received a single injection of SFK (5, 10, or 20 mg/kg) or vehicle control solution (saline) in the same volume along with LPS injection. For LMWH study, C57BL/6 J mice were received LMWH (250 IU/kg) or vehicle control solution in the same volume at 30 min before LPS injection and the LMWH treatment was then repeated every 10 h within 96 h^95^. For SKF multiple-dosing study, C57BL/6 J mice were injected with 50 mg/kg LPS along with 10 mg/kg SKF or vehicle control solution and the SKF treatment was then repeated every 12 h until 96 h. Three blinded experimenters completed the survival assessment in turns every 4 h. They received the training and performed the pre-experiment together to avoid ignoring the potential information and introduction of bias. Animals meeting any one of the following criteria were considered moribund and then euthanized by CO_2_ asphyxiation, and included in the mortality count at the time of euthanasia^96^: (1) 25% baseline body weight loss; (2) clinical or behavioral signs unresponsive to appropriate intervention persisting for 24 h, including significant inactivity, labored breathing, sunken eyes, and hunched posture; (3) surgical complications unresponsive to immediate intervention (bleeding, infection, and wound dehiscence); (4) no longer right themselves after 30 s when placed on their side.

### CLP mouse model and SKF treatment

Male C57BL/6 mice weighing 20–25 g were randomly divided into three groups (16 mice per group), vehicle-treated sham, vehicle-treated CLP, and SKF-treated CLP. High-severity CLP was performed as previously described^97^. Briefly, the mice were anesthetized with 3% isoflurane. A 1–2 cm longitudinal incision along the abdomen was made to expose the cecum. The cecum was ligated at 1.0 cm from the tip with a 2–0 sterile silk suture. In the middle of the ligation and the tip of the cecum, twice through-and-through puncture was then made with a 19-gauge needle to induce a severe septic injury. After puncturing, the cecum was gently squeezed to extrude feces and returned to the peritoneal cavity. The abdominal wall was subsequently closed in two layers. Sham mice were exposed to the same surgery, but their cecum was not punctured. All mice were injected subcutaneously (s.c.) 1 ml of prewarmed 0.9% saline solution for fluid losses as well as 0.05 mg/kg buprenorphine (s.c.) for postoperative analgesia. After abdominal closure, the treated animals were i.p. injected with normal saline or SKF (10 mg/kg dissolved in saline) every 12 h. All mice were free access to food and water. At 6 h post-surgery, six mice per group were anesthetized as described above. After echocardiography was performed, the mice hearts (ventricle) and blood samples were gathered for other assays as described above. In survival experiment, the rest mice were monitored every 2 h until 96 h after CLP surgery.

### Echocardiography assessment of cardiac function

Cardiac function was assessed by echocardiography in vivo at 6 h after LPS administration or CLP surgery. Mice were anesthetized with 2% isoflurane initially and maintained at 1% for the duration of the procedure. M-mode echocardiography of left ventricular (LV) function was performed using a Vevo-2100 high-resolution imaging unit (Visual Sonics, Toronto, Ontario, Canada). An operator is a blinded expert in animal echocardiography who routinely performed the examinations following the American Society of Echocardiography Guidelines^98^. Left ventricular end-diastolic dimension (LVEDD), left ventricular end-systolic dimension (LVESD), inter-ventricular septal thickness (IVST), and posterior wall thickness (PWT) in short axis view were measured from the M-mode tracings and averaged three consecutive sinus beats. *LV*_mass_ was calculated according to uncorrected cube assumptions using the equation, *LV*_mass_ = 1.055 **·** [(*IVST* + *LVEDD* + *PWT*)^3^ - (*LVEDD*)^3^]. The derived *LV*_mass_ was normalized for body weight and expressed in milligrams per 10 g of body weight. Ejection fraction (EF) was calculated from LV dimension using the following formula: *EF* = (*LVEDD*^3^–*LVESD*^3^)/*LVEDD*^3^ **·** 100%.

### Cell lines

Human embryonic kidney 293 T (HEK293T) cell line was obtained from ATCC (CRL-3216). The cells were authenticated by short-tandem repeat analysis by the ATCC Standards Development Organization. The cells were maintained at 37 °C and 5% CO_2_ in Dulbecco’s Eagle Medium (Gibco, NY, USA) supplemented with 10% fetal bovine serum (Gibco). The cell line was routinely tested for mycoplasma and maintained mycoplasma-free.

### Immunofluorescence microscopy

The expressions of TRPC1, TRPC6, α-SCA, CD68, DDR2, IP3R, TLR4, FLAG-tagged CaM, Myc-tagged TLR4, PDI, Rab7, and EEA1 in the ventricular tissues, cardiomyocytes, macrophages, or HEK293T cells were assessed by immunohistochemistry. OCT-embedded tissues were cryosectioned into 10-μm-thick sections. The cells were cultured on microcover glasses precoated with poly-L-lysine in 24-well plates and fixed with 4% paraformaldehyde. After permeabilizing with 0.1% Triton X-100 for 15 min and blocking with 10% goat serum, the slides were incubated with primary antibodies (Abs): TRPC1 (Alomone, ACC-010, 1:100), TRPC6 (Alomone, ACC-017, 1:100), TLR4 (Santa cruz, sc-293072, clone 25, 1:300), CaM (Santa cruz, clone G-3, sc-137079, 1:300), FLAG tag (Proteintech, 20543-1-AP, 1:100), Myc tag (Proteintech, 60003-2-Ig, clone 1A5A2, 1:500), IP3R1 (Abcam, ab264281, 1:300), PDI (Cell signalling, #3501, clone C81H6, 1:100), Rab7 (Santa cruz, sc-376362, clone B-3, 1:300), EEA1 (Santa cruz, sc-365652, clone E-8, 1:300), α-SCA (Sigma, SAB4200689, clone 5C5, 1:500), CD68 (Abcam, ab955, clone KP1, 1:50), DDR2 (Santa cruz, sc-81707, clone 3B11E4, 1:200). The sections were washed with PBS and then incubated with fluoroscence labeled secondary Abs: FITC-labeled goat anti-rabbit IgG (Jackson Immunoresearch Laboratories, 111-095-144; 1:100); FITC-labeled goat anti-mouse IgG (Jackson Immunoresearch Laboratories, 115-095-062; 1:100); Cy3-labeled goat anti-rabbit IgG (Jackson Immunoresearch Laboratories, 111-165-144 1:400); Cy3-labeled goat anti-mouse IgG (Jackson Immunoresearch Laboratories, 115-165-003 1:400); Alexa fluor 350-labeled goat Anti-rabbit IgG (Invitrogen, 11046, 1:2000); Alexa fluor 647-labeled goat anti-mouse IgG (Jackson Immunoresearch Laboratories, 115-605-003, 1:400). After several washes in PBS, sections were coverslipped with Fluoromount-G (Southernbiotech, 0100-01) or DAPI mounting medium (Southernbiotech, 0100-20). The images were acquired using a confocal microscopy (FV3000, Olympus, Tokyo, Japan).

#### Adult mouse cardiomyocyte isolation

LV cardiomyocytes from male WT, *Trpc1*^−/−^, and *Trpc6*^−/−^ mice (2 months old), pre-stimulated with LPS or vehicle for 4 h, were isolated enzymatically, as described previously with minor modifications^99^. Briefly, mice were anesthetized with sodium pentobarbital (70 mg/kg, i.p.) and the chest was quickly incised to expose the hearts. The hearts were excised and mounted onto the Langendorff apparatus and perfused with a Tyrode’s solution for 5 min at a rate of 1 ml/min at 37 °C, and then digested with sequential perfusion of enzyme buffer (1.0 M CaCl_2_, 0.036 g collagenase type II, and 0.003 g protease XIV in 15 ml Tyrode′s solution). The ventricles were cut from the hearts into a dish full of transfer buffer (1.0 M CaCl_2_ 22.5 ml, FBS 0.75 ml, and Tyrode′s solution 14.25 ml). Cell suspensions were passed through a 100-μm filter, transferred to a 15 ml conical tube, and then allowed to settle by gravity for 15 min. The cell pellets were collected from the bottom of the tubes. The protocol reproducibly yielded about 8 × 10^5^ cells per ventricle counted using a cytometer. Cell viability assessed by trypan blue dye was about 85% (from normal mice) or 22% (from LPS-challenged mice), and cells with a rod-like shape, clearly defined edges, and sharp striations were selected as viable cardiomyocytes for follow-up studies.

#### Isolation of mouse bone marrow-derived macrophages

Bone marrow cells were obtained from the tibia and femur of male WT, *Trpc1*^−/−^, and *Trpc6*^−/−^ mice (2 months old) as described previously with minor modifications^100^. Briefly, the mice were CO_2_ euthanized and the hind legs at the hip joint were cut off. The bone marrow cells were flushed out of the bones using a 25-gauge needle with 5 ml DMEM and went through a 100 µm sterile cell strainer. The cells were centrifuged and resuspended in macrophage complete medium (DMEM containing 20% L-929 conditioned medium) at 5 × 10^5^/dish. After 7 days of cultivating, contaminating nonadherent cells were eliminated and adherent cells were harvested for in vitro assays.

#### Measurement of [Ca^2+^]_i_

[Ca^2+^]_i_ levels were measured at room temperature using a digital wide-field fluorescence imaging system (TILL Photonics GmbH, Gräfelfing, Germany)^101^. Briefly, cardiomyocytes or macrophages were loaded with 2 μM Fura 2-AM in DMEM for 30 min at 37 °C in the dark, and then transferred to the standard extracellular solution (contained in mM: NaCl 125, KCl 5.4, MgCl_2_ 1.0, HEPES 10, D-glucose 11.1, NaH_2_PO_4_ 0.33, CaCl_2_ 1.8, at pH 7.4) or Ca^2+^-free solution (contained in mM: NaCl 140, KCl 5.4, EGTA 0.04, MgCl_2_ 1.0, HEPES 10, D-glucose11.1, NaH_2_PO_4_ 0.33, at pH 7.4). After pretreated with LMWH (400 μg/L), W-7 (30 μM), or SKF (0.1–30 μM) for 30 min, and/or ryanodine (100 μM) and tetracaine (10 μM) for 10 min, the cells were stimulated with 100 ng/ml LPS. Images were acquired with dual excitations at 340 and 380 nm and emission at 500 nm for Fura 2-AM. F340/F380 ratio, representing the relative intracellular Ca^2+^ concentration, was captured at 0.8 s intervals. Measurements were performed with at least 15 cells from *n* ≥ 3 mice per group.

### RNA-seq and analysis

The gene expression differences in the ventricles of WT, *Trpc1*^−/−^, and *Trpc6*^−/−^ mice were determined by RNA-seq. The sequencing libraries were generated using 2 μg of total RNA according to the stranded RNA-Seq Library Prep Kit. Sequencing was performed on the Illumina HiSeq4000 platform as detailed in Supplementary methods. Image analysis and base calling was performed using Solexa pipeline V1.8 (Off-Line Base Caller software, version 1.8). The gene & transcript expression levels (FPKM value) and significant changes were calculated using Ballgown (version 2.8.4).

### Construction and transfection of human TLR4 mutant vectors

Full-length cDNAs of human TLR4 (GenBank® accession no. U93091) and CALM2 (GenBank® accession no. CR542021) were amplified using standard PCR techniques and inserted into the pcDNA3.1(+) expression vectors (Invitrogen) with the indicated Myc or FLAG tags, respectively. Mutants of TLR4 (P714H), TLR4 (V693N), TLR4 (EWE796-798AAA) and TLR4 (A299G) were constructed. All the recombinants were verified by sequencing. HEK293T cells were transiently transfected with the above vectors using Lipofectamine 2000 for 24 h.

### Co-Immunoprecipitation (Co-IP)

Co-IP assays were carried out using Pierce™ Co-Immunoprecipitation Kits following the manufacturer’s instructions. Briefly, 2–6 μg Abs were incubated with 20 μl Protein A/G sepharose at room temperature for 1 h. The ventricular tissues or HEK293T cells were lysed with IP lysis buffer supplemented with 1 mM phenyl methane sulfonyl fluoride. After centrifuged, the lysates (1000 μg of total protein for the tissues, 300 μg for the cells) were incubated with control mouse IgG or antibody-bound sepharose at 4 °C overnight. The gathered samples were detected by western blotting.

### MST

Recombinant human full-length CALM2 and the C-terminal domain (aa644-793) of TRPC1 fusion proteins were labeled with the Monolith NT™ Protein Labeling Kit RED (Cat L001, NanoTemper Technologies, München, Germany) according to manufacturer’s instructions. Labeled proteins were kept in the concentration of 500 nM. 1 mM EDTA was added to the Apo-CALM2 protein dilution buffer and kept consistent throughout the MST measurements. The ligands (TLR4, MyD88, TRAM, four peptides TLIQ1-4, and SKF) were dissolved in ddH_2_O and diluted to 16-point serial samples. After 10-min incubation at room temperature, the samples were loaded into Monolith^TM^ standard-treated capillaries and the thermophoresis was measured at 25 °C by a Monolith NT.115 instrument (NanoTemper Technologies). *K*_*D*_ values were fitted by using the NT analysis software (NanoTemper Technologies).

### Preparation and treatment of neonatal mouse cardiomyocytes

The neonatal mouse cardiomyocytes were obtained from neonatal WT, *Trpc1*^−/−^, or *Trpc6*^−/−^ mice (2- to 3-d-old) as described previously^102^. In brief, the hearts were excised from neonatal mice under isoflurane anesthesia, minced, and dispersed with PBS. The cells were digested with 0.05% trypsin and 0.05% collagenase type II in PBS for 10 min at 37 °C. The supernatants were transferred to DMEM containing 10% FBS and the digestion was repeated four times. The suspensions were centrifuged, resuspended in a culture medium, plated in the culture flasks for 1.5 h to remove nonmyocytes, and then seeded in 60-mm culture dishes (1.5 × 10^6^ cells) or 24-well plates (5 × 10^4^ cells/well). BrdU (0.1 mM) was added throughout the culture period. The yield of one pool of cells from 10 neonatal mice was 5 × 10^6^ cells, with a viability of 95%. BrdU (0.1 mM) was added throughout the culture period to prevent the growth of cardiac fibroblasts, and the purity of the resulting cardiomyocyte cultures was validated by immunocytochemistry for myosin heavy chain-α, which is specific for cardiomyocytes. All of the cultures used in experiments were >95% cardiomyocytes by applying these criteria. After the medium was replaced by serum-free medium for 6 h, the cells were received W-7 (30 μM) or CALP1 (20 μM) at 30 min before LPS injection. Total proteins were extracted from the cells at 4 h after LPS stimulation. The cell culture supernatants were collected at 6 h after LPS stimulation for ELISA analysis. The cells on microcover glasses in the 24-well plates were fixed with 4% paraformaldehyde for immunofluorescence microscopy.

### PLA

PLA assays were carried out using Duolink PLA kits (Sigma) following the manufacturer’s instructions. Briefly, WT or *Trpc1* knockout neonatal mice cardiomyocytes were cultured on microcover glasses in 24-well plates. After stimulation with LPS, cells were fixed with 4% paraformaldehyde for 20 min at room temperature and then washed in PBS. After permeabilizing with 0.1% Triton X-100 for 15 min, cells were blocked in Duolink Blocking buffer for 30 min. The cells were incubated with the primary antibodies diluted in Duolink antibody diluent at 4 °C overnight. After washed, the cells were incubated in the appropriate Duolink secondary antibodies for 1 h at 37 °C. Then, ligation and amplification steps of the PLA were performed using the Duolink in situ Detection Reagents FarRed. the cells were coverslipped with DAPI mounting medium and the images were acquired using a confocal microscopy (FV3000, Olympus). PLA spots and nuclei were counted from a single image by using the “analyze particle” function of IJM language macro in ImageJ software^103^.

### Homology modeling and protein-protein docking studies

The amino acid sequence of cytoplasmic domain of human TLR4 (Uniport code O00206) was used as a target for homology modeling in the SWISS-MODEL server (https://swissmodel.expasy.org/)^104^. TIR domain of human TLR2 (PDB code 1Fyx, GMQE 0.73, QMEAN −2.69) was selected as the best homology model for the cytoplasmic domain of human TLR4 with the highest sequence identity (42.55). The crystal structures of apo-CaM (PDB 1QX5) and TIRAP (PDB code 4FZ5) were taken from PDB database (https://www.rcsb.org/). The schematic diagram of homology model of cytoplasmic TLR4, CaM, and TIRAP was prepared with the software Discovery Studio (DS) 4.0 (Accelrys, San Diego, California, USA). The 3D model of TLIQ2 was built using the DS 4.0 software (Accelrys). Interaction of TLIQ2 and CaM was studied by protein-protein docking tools using the Z dock module in DS^105^. In the docking process, we set an angular step size for the rotational sampling of ligand orientations to 6 degrees, generating 54,000 poses. In filter poses, a distance cutoff of 12.0 Å was employed. No restriction was set in the receptor/ligand blocked residue or receptor/ligand binding site residue. In the clustering parameter, the RMSD cutoff was set to 10 Å, and the interface cutoff was set to 10 Å. After docking, 2000 top poses were generated and clustered with a maximum number of 100. The FFT algorithm ranked all possible binding modes between receptor and ligands based on shape, desolvation energy, and electrostatics. The screening result model showing the top1 ZDOCK score was used to analyze the binding sites, indicating that the hydrophobic pocket (residues Ala2, Glu8, Asp123, Ile126, and Asp130) of CaM could interact with the atypical IQ motif (residues Lys729, Ser730, Arg731, Trp746, and Cys747) in αB-αC helix of TLR4.

### Virtual screening

The compounds’ screening against TRPC3 (PDB: 5ZBG) and TRPC6 (PDB: 5YX9) were performed using the Surflex-Dock of SYBYL-X 2.0 software (Tripos, St. Louis, MO)^106^. Briefly, Tripos Force Field (distance-dependent dielectric) was used to minimize the energies of ligands. Then, the Gasteiger-Huckel method was applied to calculate the atom charges of ligands to reach a final energy convergence gradient value of 0.001 kcal/mol. TRPC3 and TRPC6 crystal structures removed all water molecules were analyzed using the Protein Structure Preparation Tool. After adding hydrogens, the side-chain and termini treatment were fixed. Stage minimization was also applied with the AMBER FF99 force field. The protomol was generated using the Automatic with a threshold of 0.5 and bloat setting to 0. The SFXC file was built using the prepared TRPC3 and TRPC6 structures. Ligands were prepared as described above and docked as mol2 files. Cscore calculations were enabled on all Surflex docking runs, while the other docking parameters were kept as default. The automatic screening results predicted that the nonspecific blocker, SKF, ranked highest (total score: TRPC3 7.63 and TRPC6 7.91), and putative interaction sites in the CIRB domain (residues Tyr108, Val174, Arg175, and Lys888). The docking was performed using the default settings and PyMol (http://www.pymol.org) was used for generating figures.

### Statistical analysis

Data are represented as mean ± S.E.M.. Statistical analysis was performed with the SPSS Statistics v.23.0 software (IBM Corp., Armonk, NY, USA). Pairwise comparisons were performed using a two-tailed *t*-test. For experiments with more than two groups, data were analyzed by one-way ANOVA followed by Tukey’s (equal varance) or Games Howell’s (not equal varance) post hoc multiple comparison test. For the Kaplan-Meier curves, *P* values were assessed by the log-rank test. The data difference was considered significant when *P* value was less than 0.05.

### Reporting summary

Further information on research design is available in the Nature Portfolio Reporting Summary linked to this article.

## Supplementary information

Supplementary information

Reporting Summary

## Data Availability

The data that support this study are available from the corresponding author upon request. The RNA-seq data generated in this study have been deposited in the GEO database (https://www.ncbi.nlm.nih.gov/geo/) under accession code GSE217156. Information of the structure of proteins TLR2 (PDB 1FYX), CaM (PDB 1QX5), TRPC6 (PDB 5YX9), TIRAP (PDB 4FZ5), and TRPC3 (PDB 5ZBG) were obtained from the PDB database (https://www.rcsb.org/). The amino acid sequence of cytoplasmic domain of human TLR4 (Uniport code O00206) was obtained from the Uniport database (https://www.uniprot.org/). Source data are provided with this paper.

## Source data

Source Data
