# Supplementary material for: TRPC channels blockade abolishes endotoxemic cardiac dysfunction by hampering intracellular inflammation and Ca2+ leakage
Source: Nat Commun. 2022 Dec 2;13:7455. doi: 10.1038/s41467-022-35242-0 (PMC9718841; doi:10.1038/s41467-022-35242-0)
Supplement: Supplementary file 1 — Supplementary information [file 41467_2022_35242_MOESM1_ESM.pdf]

Supplementary information

TRPC channels blockade abolishes endotoxemic cardiac dysfunction by hampering intracellular inflammation and Ca^{2+} leakage

Na Tang[1,2,8], Wen Tian[3,4,8], Guang-Yuan Ma[1,2,8], Xiong Xiao[3,4,8], Lei Zhou[1,2], Ze-Zhi Li[1,2], Xiao-Xiao Liu[3,4], Chong-Yao Li[5], Ke-Han Wu [1,2], Wenjuan Liu[1], Xue-Ying Wang[3,4], Yuan-Yuan Gao[3,4], Xin Yang[1,2], Jianzhao Qi[2], Ding Li[2], Yang Liu[6], Wen-Sheng Chen[6,7], Jinming Gao[1,2], Xiao-Qiang Li[3,4]* & Wei Cao[1,2]*

[1] Department of Pharmacy, School of Chemistry & Pharmacy, Northwest A&F University, Yangling, Shaanxi, China

[2] Shaanxi Key Laboratory of Natural Products & Chemical Biology, Northwest A&F University, Yangling, Shaanxi, China

[3] Department of Pharmacology, School of Pharmacy, Fourth Military Medical University, Xi'an, Shaanxi, China

[4] Key Laboratory of Gastrointestinal Pharmacology of Chinese Materia Medica of the State Administration of Traditional Chinese Medicine, Fourth Military Medical University, Xi'an, Shaanxi, China

[5] Department of Pharmacy, Xi'an No.3 Hospital, the Affiliated Hospital of Northwest University, Xi'an, Shaanxi, China

[6] Department of Cardiovascular Surgery, Xijing Hospital, Fourth Military Medical University, Xi'an, Shaanxi, China.

[7] Department of Cardiovascular Surgery, Xi'an Gaoxin Hospital, Xi'an, Shaanxi, China

[8] These authors contributed equally: Na Tang, Wen Tian, Guang-Yuan Ma, Xiong Xiao

* Correspondence should be addressed to W Cao (caowei@nwafu.edu.cn, Orcid 0000-0003-3863-137X), X.Q. Li (xxqqli@fmmu.edu.cn, Orcid 0000-0003-2463-8298).

Inventory of supplementary information

Supplementary methods

Supplementary figures

Supplementary Fig. 1 TRPC1 and TRPC6 are up-regulated by LPS challenge in hearts and boost endotoxemic cardiac dysfunction.

Supplementary Fig. 2 The effects of *Trpc1* or *Trpc6* knockout and IP3R inhibition on the intracellular Ca^{2+} release in mice cardiomyocytes.

Supplementary Fig. 3 The effects of *Trpc1* or *Trpc6* knockout and IP3R inhibition on intracellular Ca^{2+} release in LPS-stimulated macrophages.

Supplementary Fig. 4 The effects of LMWH treatment on the cardiac function and survivability of LPS-challenged mice.

Supplementary Fig. 5 Cluster heatmap of differential gene expression in the heart tissues using RNA-seq.

Supplementary Fig. 6 The mRNA expressions of partial TLR signaling pathway-related genes in the heart tissues.

Supplementary Fig. 7 The intracellular interaction and location of CaM and TLR4 in cardiomyocytes.

Supplementary Fig. 8 The CaM interaction with the TIR domain of TLR4.

Supplementary Fig. 9 The synthesized peptides containing nonclassical IQ-like motifs within TIR domain of TLR4.

Supplementary Fig. 10 The pleiotropic roles of TRPC in regulating IP3R1 and the effects of SKF96365 (SKF) on the protein expressions of TRPCs.

Supplementary Fig. 11 TRPC blocker SKF cures septic cardiac dysfunction.

Supplementary Fig. 12 Schematic representation of the proposed mechanisms.

Supplementary tables

Supplementary table 1 Echocardiographic assessment about left ventricular (LV) function of LPS-challenged *Trpc1*$^{-/-}$ or *Trpc6*$^{-/-}$ mice.

Supplementary table 2 The effects of LWMH on the LV function of LPS-challenged mice.

Supplementary table 3 The effects of SKF96365 on the LV function of LPS-challenged mice.

Supplementary table 4 The effects of SKF96365 on the LV function of CLP mice.

Supplementary table 5 Virtual screen for small-molecule antagonist of TRPC3 and TRPC6

Supplementary table 6 Primer sequences for qPCR.

Supplementary table 7 siRNA oligonucleotides sequences.

Supplementary references

Supplementary methods

Measurement of mean arterial blood pressure (MAP). Mice were anesthetized with sodium pentobarbital (50 mg/kg, i.p.). Chronic indwelling catheters were placed in the femoral artery for MAP and heart rate measurement. Heparin sodium (100 IU/ml) solution was infused for the maintenance of catheter patency. The catheter was connected to a pressure transducer and data were collected by a precalibrated PowerLab/4SP recording system (AD Instruments Pty Ltd., Bella Vista, New South Walles, Australia). Following the completion of the surgical procedure, cardiovascular parameters were allowed to stabilize for 30 min. After LPS (50 mg/kg) was i.p. administered, MAP and heart rate were monitored over a 6-h period.

Histology and immunohistochemistry. The ventricular tissues were fixed in buffered formalin, dehydrated in graded ethanol, embedded in paraffin, and serially sectioned at 4-μm thickness. Standard hematoxylin and eosin staining and immunohistochemical staining with CCL3/MIP-1α (R&D systems, 1:50) antibody (Ab) were performed on these sections. The samples were examined and photographed with a Nikon Eclipse 80i microscope. All digital photographs were taken and measured in the same parameter setting. Image analysis was performed with ImageJ 1.49v (National Institutes of Health, Bethesda, MD, USA). The mean integrated optical density was assessed for manually cropped areas of uniform intensity in the field. The individual who analyzed the histologic samples was blinded to the treatment. Each data point is represented by 12 images from 4 animals.

Quantitative Real-time PCR (qRCR). Total RNA was extracted from ventricular tissues using RNeasy Mini Kits (Qiagen) according to the manufacturer's protocol. cDNA was generated from 1 μg of RNA via SuperScript^{TM} III Reverse Transcriptase (Invitrogen). Primers were obtained from Sangon Biotechnology (Shanghai, China) and their sequences were given in Supplementary table 6. Real-time PCR was performed using SYBR Green PCR Master Mix (Bio-Rad) on ViiA 7 Real-time PCR System (Applied Biosystems, Foster city, CA, USA). Threshold cycle (Ct) was obtained from the PCR reaction curve. Relative mRNA expression was normalized to the internal reference gene *GAPDH* and calculated using the $X = 2^{-\Delta\Delta CT}$ equation, $\Delta\Delta C_T = C_{T(samples)}\text{-}C_{T(GAPDH)}$.

Western blotting. Total proteins were extracted from cardiac tissues or cultured cells by RIPA buffer supplemented with protease inhibitor cocktail. Nuclear and cytoplasmic extracts were prepared using the nuclear and cytoplasmic protein extraction kit. Extracted proteins were quantified using the BCA protein assay kits. Western blotting was performed according to the standard protocol. In brief, equal amounts of proteins were denatured and separated by sodium dodecyl sulfate (SDS)-PAGE on 8% to 15% separating gels. The proteins were transferred to nitrocellulose membranes and the membranes were blocked in 5% BSA diluted in PBS-0.05% Tween-20 (PBS-T) for 2 h. Blots were incubated with primary Abs as follows: TRPC1 (Alomone, ACC-010, 1:200), TRPC3 (Alomone, ACC-016, 1:200), TRPC4 (Alomone, ACC-018, 1:200), TRPC5 (Alomone, ACC-020, 1:200), TRPC6 (Alomone, ACC-017, 1:200), TRPC7 (Alomone, ACC-066, 1:200), NF-κB p65 (Proteintech, 10745-1-AP, 1:1000), p-ERK1/2 (Cell signalling, #4370, clone D13.14.4E, 1:1000), ERK1/2 (Cell signalling, #4695, clone 137F5, 1:1000), p-JNK (Cell signalling, #4668, clone 81E11, 1:1000), JNK (Cell signalling, #9252, 1:1000), p-p38 (Cell signalling, #4511, clone D3F9, 1:1000), p38 (Cell signalling, #8690, clone D13E1, 1:1000), TLR4 (Santa cruz, sc-293072, clone 25, 1:1000), MyD88 (Santa cruz, sc-74532, clone E-11, 1:1000), TRIF (Santa cruz, sc-514384, clone E-7, 1:1000), TRAM (Proteintech, 12705-1-AP, 1:1000), TIRAP (Cell signalling, #13077, clone D6M9Z, 1:1000), p-IRAK1 (Abbkine, ABP54916, 1:1000), IRAK1 (Proteintech, 10478-2-AP, 1:1000), IRAK4 (Proteintech, 18221-1-AP, 1:1000), TRAF6 (Santa cruz, sc-8409, clone D-10, 1:3000), p-IRF-3 (Biorbyt, orb571465, 1:1000), IRF-3 (Proteintech, 11312-1-AP, 1:1000), CaM (Santa cruz, sc-137079, clone G-3, 1:1000), NFAT3 (Cell signalling, #2183, clone 23E6, 1:1000), FLAG tag (Proteintech, 20543-1-AP, 1:3000), Myc tag (Proteintech, 60003-2-Ig, clone 1A5A2, 1:3000), IP3R1 (Abcam, ab264281, 1:2000), IP3R2 (Santa cruz, sc-398434, clone A-5, 1:1000), IP3R3 (Cohesion, CQA4813, 1:1000), RYR2 (SAB, #48554, 1:500), GAPDH (Proteintech, 60004-1-Ig, clone 1E6D9, 1:5000), histone (Proteintech, 17168-1-AP, 1:1000), and α-Tubulin (Proteintech, 66031-1-Ig, clone 1E4C11, 1:1000). After incubated with horseradish peroxidase (HRP)-conjugated goat anti-mouse secondary Ab (Abbkine, A21010, 1:10000) or HRP-conjugated goat anti-rabbit secondary Ab (Abbkine, A21020, 1:10000), the membranes were rinsed in PBS-T, detected with ECL detection kit, and exposed in a Tanon-5200 Imaging System (Tanon Science and Technology Co., Ltd., Shanghai, China). Quantitative image analysis was performed with ImageJ 1.49v software.

Calcineurin activity analysis. Calcineurin activity was analyzed using Calcineurin Assay Kit according to the manufacturer's instructions. Briefly, ventricular tissue was homogenized in lysis buffer containing protease inhibitors on ice to extract soluble proteins. The protein samples were desalted using Bio-Spin 6 columns to remove free phosphates. The assay buffer containing RII phosphopeptide as the substrate for calcineurin was added. The samples were incubated for 20 min at room temperature. The reactions were stopped by adding Biomol Green reagent and then incubated at room temperature for 30 min. The absorbance was determined at 620 nm. Ethylene glycol bis (2-aminoethyl ether)-*N*,*N*,*N*',*N*'-tetraacetic acid (EGTA) was used as the calcineurin inhibitor. Protein contents were determined using BCA assay kit.

RNA-seq and analysis. The gene expression differences in the ventricles of WT, *Trpc1*$^{-/-}$, and *Trpc6*$^{-/-}$ mice were determined by RNA-seq[1]. DNA library preparation and sequencing were conducted by KangChen Bio-tech (Shanghai, China).

DNA library preparation and sequencing

Total RNA was isolated using the TRIzol reagent (Invitrogen) according to the manufacturer's protocol. Total RNA-Seq libraries were generated from 2 μg of total RNA using KAPA Stranded Total RNA LT Sample Prep Kit with Ribo-Zero Gold (Illumina, San Diego, CA) following the manufacturer's directions. Briefly, cytoplasmic and mitochondrial ribosomal RNA (rRNA) was removed using biotinylated, target-specific oligos combined with Ribo-Zero rRNA removal beads. Following purification, the depleted RNA was fragmented into small pieces using divalent cations at 94°C for 2 min. Cleaved RNA fragments were then copied into first strand cDNA using reverse transcriptase and random primers followed by second strand cDNA synthesis using DNA Polymerase I and RNase H. Strand specificity was achieved by replacing dTTP with dUTP during second strand synthesis. The double stranded cDNA fragments were blunted using T4 DNA polymerase, Klenow DNA polymerase and T4 polynucleotide kinase. A single 'A' nucleotide was added to the 3' ends of the blunt DNA fragments using a Klenow fragment (3' to 5' exo minus) enzyme. The cDNA fragments were ligated to double stranded adapters using T4 DNA ligase. The ligated products were enriched by PCR amplification. The final cDNA libraries were checked for quality and quantified using capillary electrophoresis. Sequencing was performed on an Illumina HiSeq 4000 in a 2×150 bp format.

Data analysis

Image analysis and base calling were performed using Solexa pipeline V1.8 (Off-Line Base Caller software, version 1.8). Trimmed reads (pass FastQC 0.11.5 filter) were aligned to the mouse reference genome (GenCode mm10) and the mouse transcriptome (GenCode mm10) using Hisat2 software (version 2.0.5). Transcriptional abundance estimation was completed via StingTie software (version 1.3.1c) according to the official database annotation information. The gene & transcript expression levels (FPKM value) and significant changes in gene & transcript expression were then calculated using Ballgown (version 2.8.4). Gene Ontology (GO) analysis was applied in the standard enrichment computation method to determine the roles of these differentially expressed genes played in these GO terms or biological pathways. Three biologic replicates were independently performed for both treatment and control groups. KEGG pathway enrichment analyses were conducted by gseKEGG function in clusterProfiler package. The adjusted P-value < 0.05 was set as the cut-off criteria.

Expression and purification of recombinant protein. Expression and purification of the C-terminal domain (aa644-793) of human TRPC1 fusion protein was accomplished by Zoonbio Biotechnology Co., Ltd (Nanjing, China). The recombinant expression plasmid pCZN1-TRPC1 was constructed and then transformed into *Escherichia coli* TOP10 cells. The protein expression was induced by isopropyl β-D-1-thiogalactopyranoside and the protein was purified by nickel ions chelating resin. Finally, the protein was separated by SDS-PAGE and the concentration was determined using a BCA protein assay kit.

Native gel electrophoresis Native gel electrophoresis was performed according to the standard protocol[2]. Briefly, recombinant human CALM2 protein (3.5 μM) and peptides (20 μM, dissolved in PBS) were mixed in a total volume of 20 μl and incubated at room temperature for 6 h. The loading dye (125 mM Tris-HCl, pH 6.8; 20% glycerol; 1% dithiothreitol) was added to the reaction solution and the samples were resolved on native polyacrylamide gels at 30 mA for 5.0-6.5 h at 4°C. The gels were stained with 0.25% (w/v) Coomassie brilliant blue R250 solution. The images were recorded using a Tanon-5200 Imaging System.

Peptide synthesis and purification. Four peptides were synthesized by ChinaPeptides Co., Ltd (Shanghai,

China). The synthesis was carried out by the standard fluorenylmethyloxycarbonyl (Fmoc) solid-phase peptide synthesis (SPPS) method[3]. Fmoc-Val-Wang resin was loaded into a fritted column, equipped with a plastic cap, and washed with dimethylformamide (DMF). The deprotection under condition of 20% piperidine in DMF continued for 15 min, and then the resin was washed with DMF. In a small vial, 3 equivalents of Fmoc-protected amino acid was pre-activated by combining it with 3 equivalents of HBTU, 6 equivalents of N,N'-diisopropylethylamine in DMF. Then, the coupling solution was added to the resin and agitated for 20 min. To obtain the peptide in the free acid form, the ester linkage was cleaved using a solution of trifluoroacetic acid and water (95:5) for 25 min. The filtrate was collected and purified by high performance liquid chromatography. Electrospray ionization mass spectrometry was used to identify peptides.

Circular dichroism (CD) spectroscopy. CD spectra of the peptides TLIQ1 - TLIQ4 were recorded on a Chirascan spectropolarimeter (Applied Photophysics, Surrey, UK). The peptide concentration was 0.2 mg/ml in Tris-HCl buffer containing 20 mM Tris-HCl (pH 7.5), 250 mM NaCl, and 1 mM EDTA. The CD spectra were measured from 190 nm to 270 nm at a scanning speed of 100 nm/min in a quartz optical cell with a path length of 0.1 cm. All spectra were recorded in 0.5 nm wavelength increments with a 1.0 nm bandwidth. The final spectra were an average of 3 scans recorded at room temperature.

ELISA assay. The blood samples were collected and centrifuged for 10 min at 3,000 rpm to obtain serum. The supernatants of cultured neonatal mice cardiomyocytes were collected and stored at -80°C until analysis. The TNF-α, IFN-β, IL-1β, IL-6, Troponin-I, and Troponin-T levels in these samples were measured using commercially available kits according to the manufacturer's instructions. Optical density values were collected on a Synergy HTX multi-mode microplate reader (BioTek, Winooski, USA). The OriginPro 2018C software (OriginLab Corp.) was used for data analysis.

siRNA interference. Mouse *Itpr1*, *Itpr2*, *Ryr2* siRNA and control siRNA were designed and synthesized by HanBio Biotechnology (Shanghai, China) for knockdown experiments. Sequences of siRNA oligonucleotides were given in Supplementary table 7. Neonatal mice cardiomyocytes and bone-marrow-derived macrophages were transfected using RNAFit transfection reagent (HanBio

Biotechnology) for 72 h. Protein knockdown efficacy was confirmed by western blotting.

Isolation of mouse cardiac-resident macrophages (cMacs). cMacs were obtained from WT, *Trpc1*$^{-/-}$, and *Trpc6*$^{-/-}$ male mice (2 months old)[4]. Hearts were swiftly excised and gently flushed with 20 mL cold PBS. Thereafter, tissue was minced into small pieces and subjected to enzymatic digestion with 450 U/mL collagenase I, 125 U/mL collagenase XI, 60 U/mL DNase I, and 60 U/mL hyaluronidase (Sigma-Aldrich) in 1 mL PBS for 35 min at 37°C under gentle agitation. Tissue fragments were then triturated, filtered through a 40 mm nylon mesh, and pelleted by centrifugation (400×g for 5 min at 4°C). Cells were re-suspended in fluorescence-activated cell sorting (FACS) buffer (PBS with 1% foetal calf serum + 0.1% bovine serum albumin). cMacs were FACS-purified using an Aria III cell sorter (BD Biosciences, Franklin Lakes, NJ, USA). Unstained and single stained controls were used to set up the compensation for the FACS sorter. The cellular identity of cMacs was detected as CD45^{+} CD11b^{+} F4/80^{+} using immunofluorescence staining with following antibodies: CD45.2-APC (eBioscience, 17-0454-81, clone 104, 0.5 µg Ab per 10^{7} cells), F4/80-PE (eBioscience, 12-4801-80, clone BM8, 0.25 µg Ab per 10^{7} cells), and CD11b-PerCP.Cy5.5 (eBioscience, 45-0112-80, clone M1/70, 0.25 µg Ab per 10^{7} cells). FlowJo software (V10.0.7) was used to analyze FACS data.

Supplementary figures

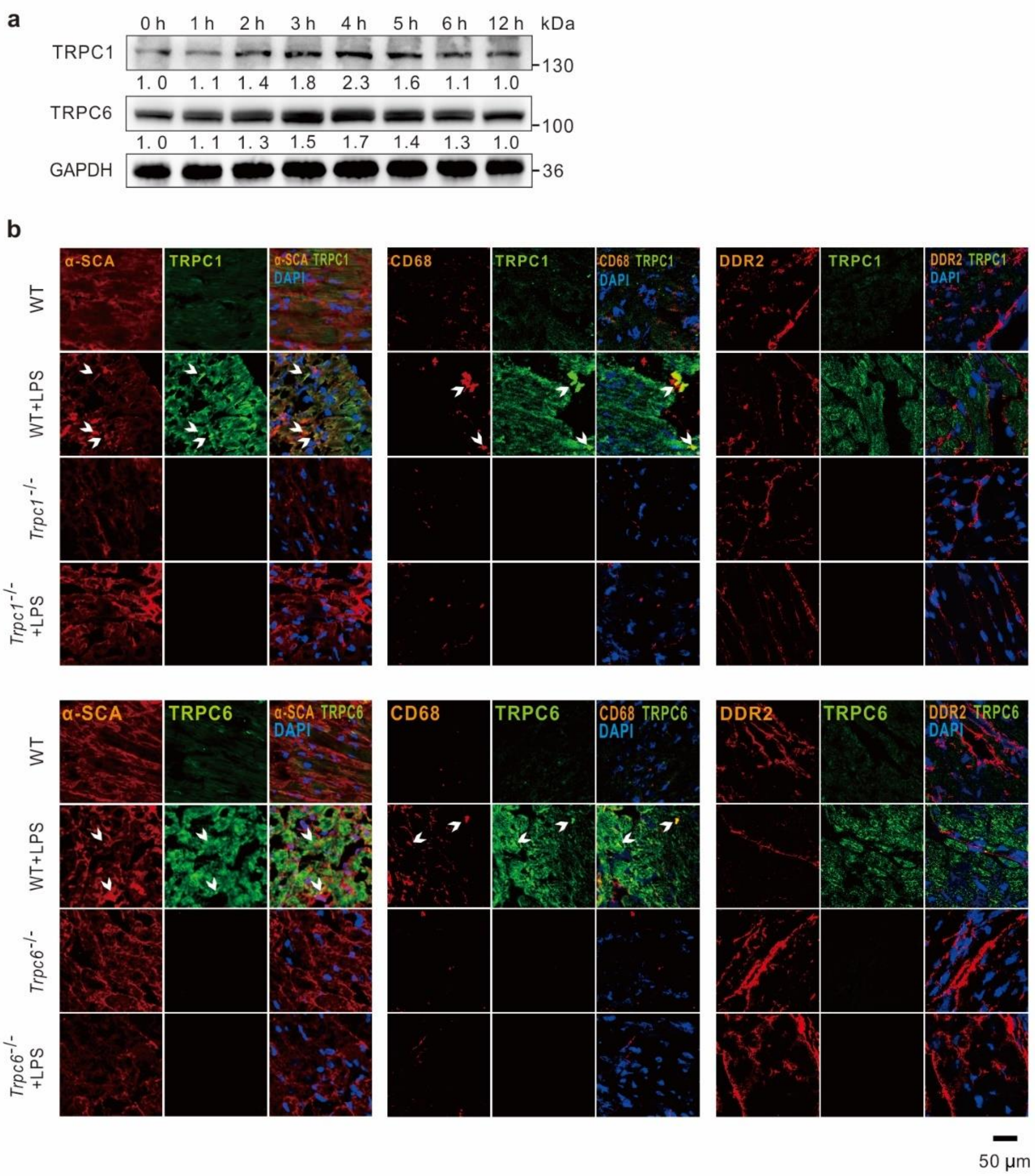

Supplementary Fig. 1 TRPC1 and TRPC6 are up-regulated by LPS challenge in hearts and boost endotoxemic cardiac dysfunction. a The time-dependent changes of TRPC1 and TRPC6 protein expressions in the hearts of C57BL/6 mice challenged with vehicle (saline) or LPS (i.p., 50 mg/kg body weight) during 12 h (pooled tissues from 3 male mice per sample, *n* = 3 biological independent experiments). **b** Representative immunofluorescent photomicrographs of TRPC1 or TRPC6 (green) localization. Cardiomyocytes labeled with α-sarcomeric actin (α-SCA) (red), macrophages labeled with CD68 (red), or fibrocytes labeled with DDR2 (red) in ventricles of WT, *Trpc1*$^{-/-}$, and *Trpc6*$^{-/-}$ mice 6 h after LPS challenge. Blue indicates cellular nuclei stained with DAPI. The colocalization is shown in yellow (white arrows) (*n* = 6 images from 3 male mice per group). Source data are provided as a Source Data file.

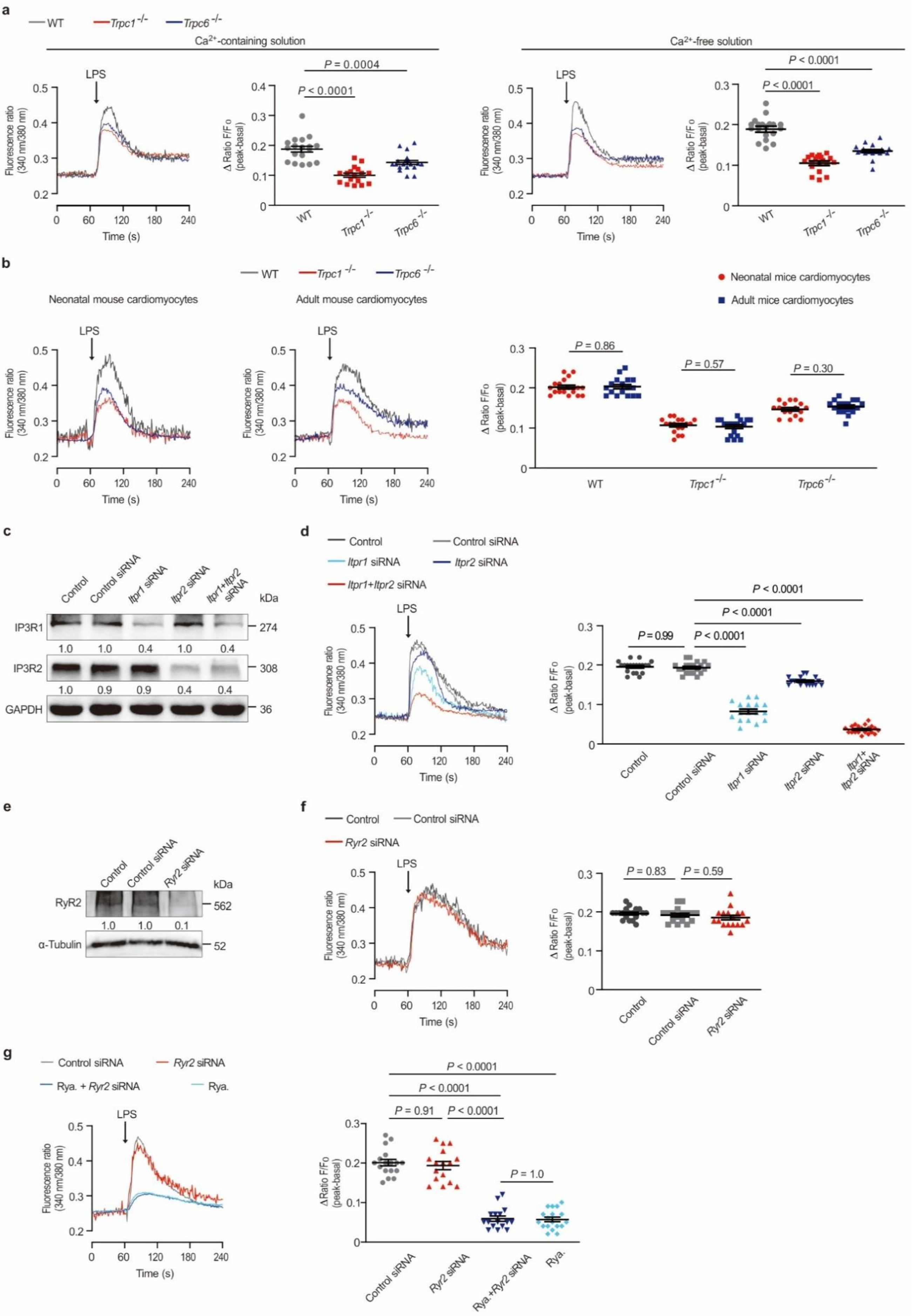

Supplementary Fig. 2 The effects of *Trpc1* or *Trpc6* knockout and IP3R inhibition on the intracellular

Ca^{2+} release in mice cardiomyocytes. a *Trpc1* or *Trpc6* knockout inhibits LPS-triggered intracellular Ca^{2+} release in adult mice cardiomyocytes in Ca^{2+}-containing and Ca^{2+}-free extracellular solution, respectively. Typical trace recordings (left panel) and the statistical analysis (right panel) are shown (mean ± SEM, *n* = 15-20 cells from 3 male mice per group). Statistical significance was determined using the one-way ANOVA with Tukey's multiple comparisons test. In Ca^{2+}-containing solution, exact *P* value = 5.9×10^{-9} (WT vs *Trpc1*$^{-/-}$). In Ca^{2+}-free extracellular solution, exact *P* value =5.1×10^{-9} (WT vs *Trpc1*$^{-/-}$) and 1.2×10^{-8} (WT vs *Trpc6*$^{-/-}$). **b** *Trpc1* or *Trpc6* knockout inhibits LPS-triggered intracellular Ca^{2+} release in both neonatal and adult mice cardiomyocytes in Ca^{2+}-free extracellular solution. Typical trace recordings (left panel) and the statistical analysis (right panel) are shown (mean ± SEM, *n* = 15-20 cells from 3 mice per group). Statistical significance was determined using the two-tailed Student's *t*-test. **c** IP3R1 and IP3R2 protein expressions in neonatal mice cardiomyocytes transfected with *Itpr1* and *Itpr2* siRNA (*n* = 3 biological independent experiments). **d** *Itpr1* and *Itpr2* knockdown inhibit LPS-triggered intracellular Ca^{2+} release in neonatal mice cardiomyocytes in Ca^{2+}-free extracellular solution. Typical trace recordings (left panel) and the statistical analysis (right panel) are shown (mean ± SEM, *n* = 15-20 cells from 3 mice per group). Statistical significance was determined using the one-way ANOVA with Games Howell's multiple comparisons test. Exact *P* value = 9.6×10^{-13} (Control siRNA vs *Itpr1* siRNA), 1.5×10^{-8} (Control siRNA vs *Itpr2* siRNA), and 7.1×10^{-13} (Control siRNA vs *Itpr1*+ *Itpr2* siRNA). **e** RyR2 protein expression in neonatal mice cardiomyocytes transfected with *Ryr2* siRNA (*n* = 3 biological independent experiments). **f** The effect of *Ryr2* knockdown on LPS-triggered intracellular Ca^{2+} release in neonatal mice cardiomyocytes in Ca^{2+}-free extracellular solution. Typical trace recordings of Ca^{2+} response (left panel) and the statistical analysis (right panel) are shown (mean ± SEM, *n* = 15-20 cells from 3 mice per group). Statistical significance was determined using the one-way ANOVA with Tukey's multiple comparisons test. **g** The effects of ryanodine (Rya.) on LPS-induced Ca^{2+} release in control or *Ryr2* siRNA-transfected neonatal mice cardiomyocytes in Ca^{2+}-free extracellular solution. Typical trace recordings (left panel) and the statistical analysis (right panel) are shown (mean ± SEM, n = 15-20 cells from 3 mice per group). Statistical significance was determined using the one-way ANOVA with Tukey's multiple comparisons test. Exact *P* value = 5.9×10^{-13} (Control siRNA vs *Ryr2* siRNA+Rya.), 5.9×10^{-13} (Control siRNA vs Rya.), and 5.9×10^{-13} (*Ryr2* siRNA+Rya. vs Rya.). Source data are provided as a Source Data file.

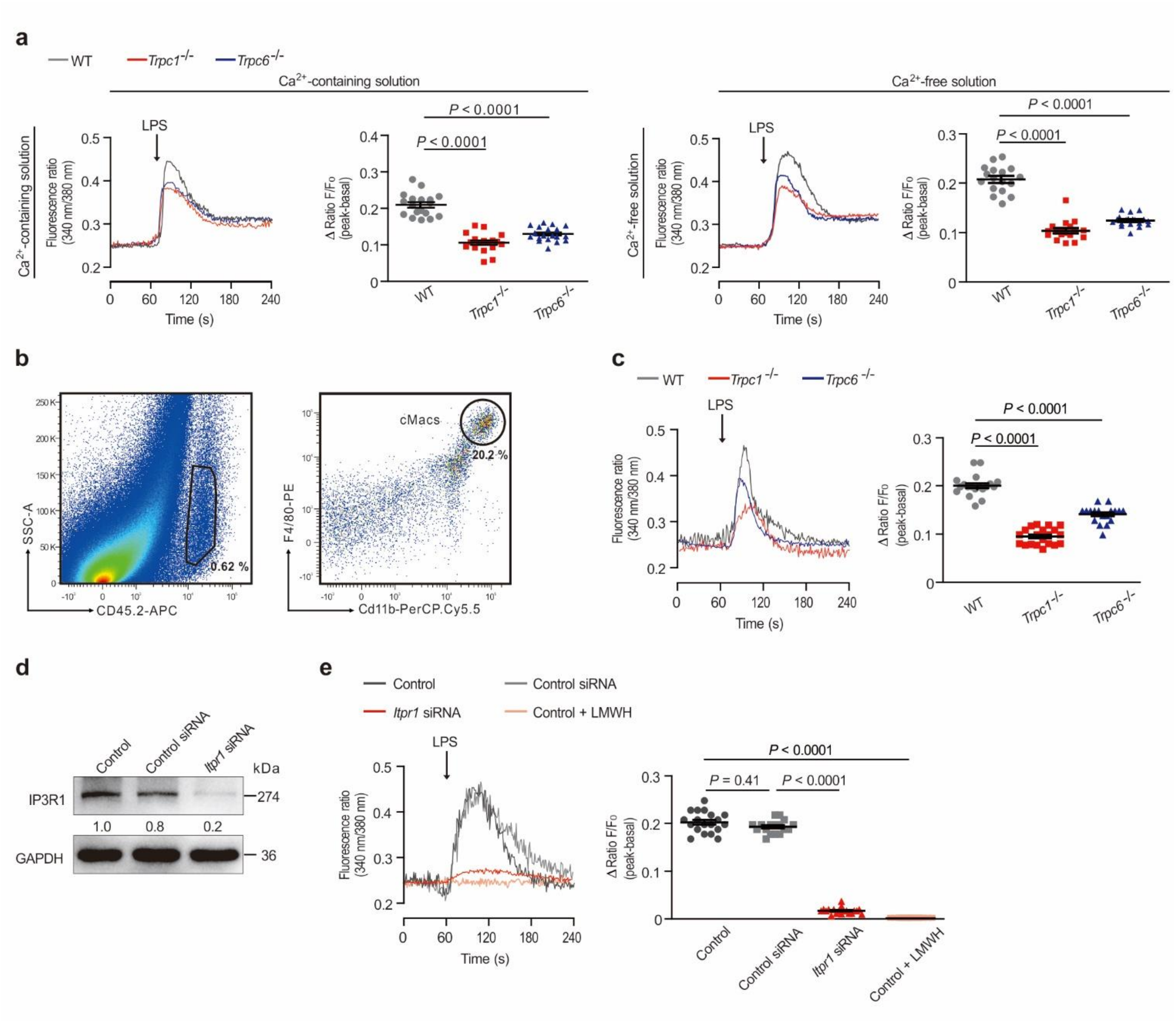

Supplementary Fig. 3 The effects of *Trpc1* or *Trpc6* knockout and IP3R inhibition on intracellular Ca^{2+} release in LPS-stimulated macrophages. a *Trpc1* or *Trpc6* knockout inhibits LPS-triggered intracellular Ca^{2+} release in bone-marrow-derived macrophages (BMMs) in Ca^{2+}-containing and Ca^{2+}-free extracellular solution, respectively. Typical trace recordings (left panel) and the statistical analysis (right panel) are shown (mean ± SEM, *n* = 15-20 cells from 3 male mice per group). Statistical significance was determined using the one-way ANOVA with Tukey's multiple comparisons test. In Ca^{2+}-containing solution, exact *P* value = 5.1× 10^{-9} (WT vs *Trpc1*$^{-/-}$) and 5.1× 10^{-9} (WT vs *Trpc6*$^{-/-}$); In Ca^{2+}-free extracellular solution, exact *P* value = 5.1× 10^{-9} (WT vs *Trpc1*$^{-/-}$) and 5.1× 10^{-9} (WT vs *Trpc6*$^{-/-}$). **b** The gating strategy used to prepare cardiac-resident macrophages (cMacs), detected as $CD45^{+}$ $CD11b^{+}$ $F4/80^{+}$, by flow cytometry and cell sorting. **c** *Trpc1* or *Trpc6* knockout inhibits LPS-triggered intracellular Ca^{2+} release in cMacs in Ca^{2+}-free extracellular solution. Typical trace recordings (left panel) and statistical analysis (right panel) are shown

(mean ± SEM, *n* = 15-20 cells from 3 male mice per group). Statistical significance was determined using the one-way ANOVA with Tukey's multiple comparisons test. Exact *P* value = 5.1×10^{-9} (WT vs *Trpc1*$^{-/-}$) and 5.1×10^{-9} (WT vs *Trpc6*$^{-/-}$). **d** IP3R1 protein expression in BMMs transfected with *Itpr1* siRNA (*n* = 3 biological independent experiments). **e** *Itpr1* knockdown and low molecular weight heparin (LMWH) inhibits LPS-triggered intracellular Ca^{2+} release in BMMs in Ca^{2+}-free extracellular solution. Typical trace recordings (left panel) and the statistical analysis (right panel) are shown (mean ± SEM, *n* = 15-20 cells from 3 male mice per group). Statistical significance was determined using the one-way ANOVA with Games Howell's multiple comparisons test. Exact *P* value = 1.0×10^{-12} (Control vs Control+LMWH) and 8.6×10^{-13} (Control siRNA vs *Itpr1* siRNA). Source data are provided as a Source Data file.

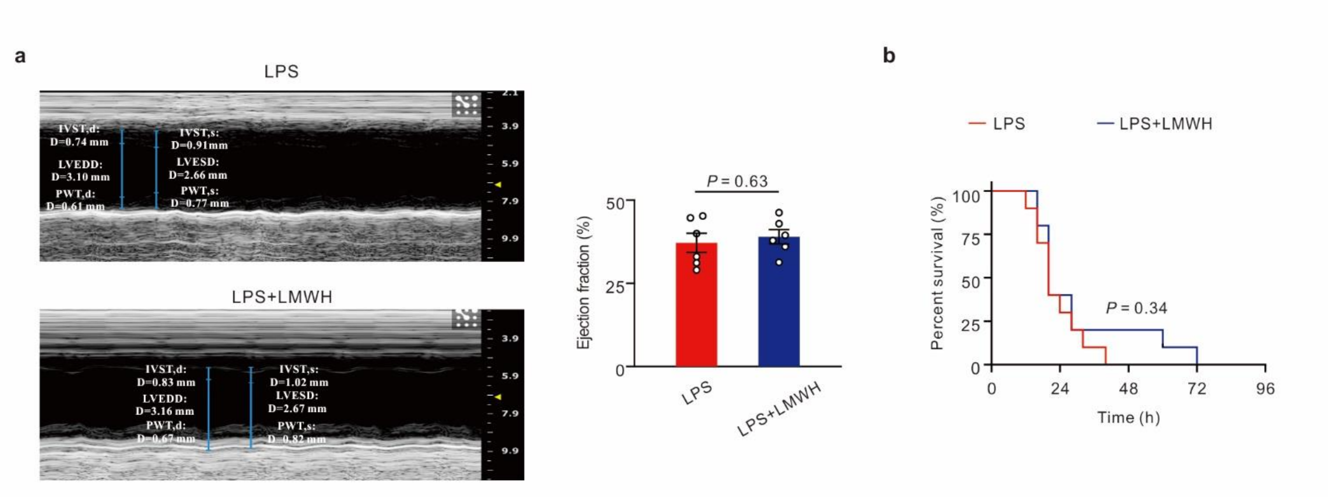

Supplementary Fig. 4 The effects of LMWH treatment on the cardiac function and survivability of LPS-challenged mice. a The effects of LMWH treatment on the cardiac function of LPS-challenged mice. Typical heart M-mode echocardiography still and ejection fraction are shown (mean ± SEM, *n* = 6 male mice per group). Statistical significance was determined using the one-way ANOVA with Tukey's multiple comparisons test. **b** Kaplan-Meier survival curves of LMWH-treatment on mice subjected to 50 mg/kg LPS (*n* = 10 male mice per group). Statistical significance was determined using the log-rank test. Source data are provided as a Source Data file.

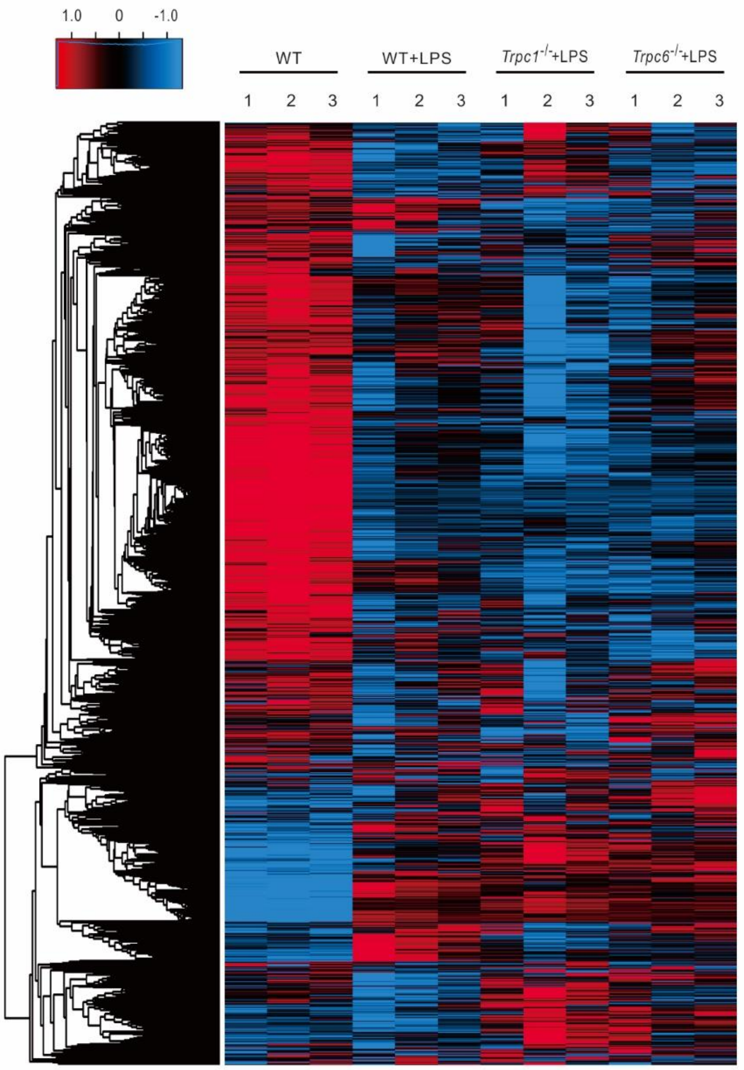

Supplementary Fig. 5 Cluster heatmap of differential gene expression in the heart tissues using RNA-seq (*n* = 3 male mice per group).

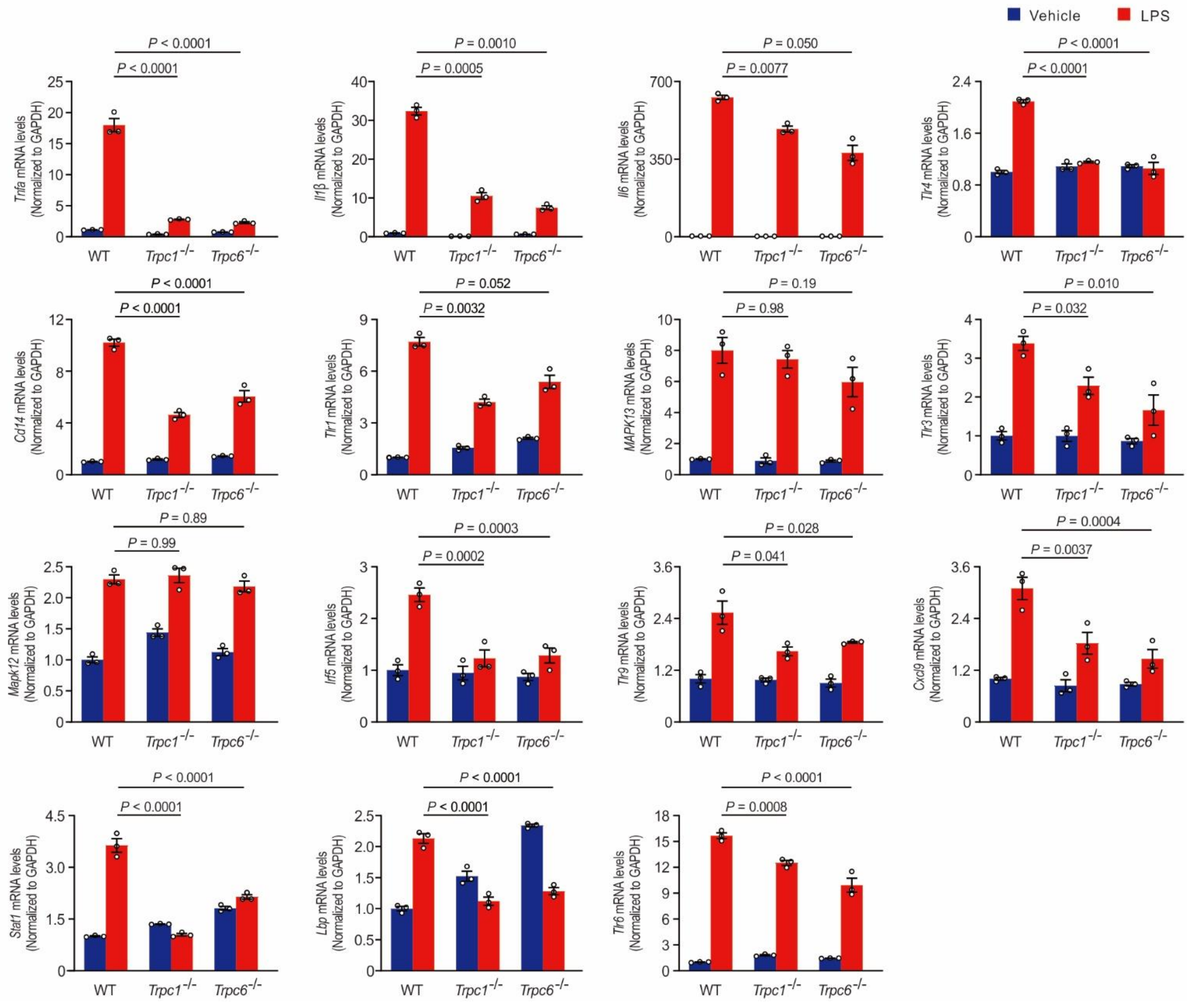

Supplementary Fig. 6 The mRNA expressions of partial TLR signaling pathway-related genes in the heart tissues (*n* = 3 male mice with triplicate measurements taken, mean ± SEM). Statistical significance was determined using one-way ANOVA with Game Howell's multiple comparisons test for *IL1β, IL6*, and *Tlr1*, and Tukey's multiple comparisons test for other genes. *Tnfa*, exact *P* value = 1.5×10^{-10} (WT+LPS vs *Trpc1*$^{-/-}$+LPS) and 1.0×10^{-10} (WT+LPS vs *Trpc6*$^{-/-}$+LPS); *Tlr4*, exact *P* value = 6.9×10^{-8} (WT+LPS vs *Trpc1*$^{-/-}$+LPS) and 2.3×10^{-8} (WT+LPS vs *Trpc6*$^{-/-}$+LPS); *CD14*, exact *P* value = 9.0×10^{-9} (WT+LPS vs *Trpc1*$^{-/-}$+LPS) and 2.6×10^{-7} (WT+LPS vs *Trpc6*$^{-/-}$+LPS); *Stat1*, exact *P* value = 1.2×10^{-9} (WT+LPS vs *Trpc1*$^{-/-}$+LPS) and 6.4×10^{-7} (WT+LPS vs *Trpc6*$^{-/-}$+LPS); *Lbp*, exact *P* value = 6.4×10^{-7} (WT+LPS vs *Trpc1*$^{-/-}$+LPS) and 4.0×10^{-6} (WT+LPS vs *Trpc6*$^{-/-}$+LPS); *Tlr6*, exact *P* value = 2.0×10^{-6} (WT+LPS vs *Trpc6*$^{-/-}$+LPS). Source data are provided as a Source Data file.

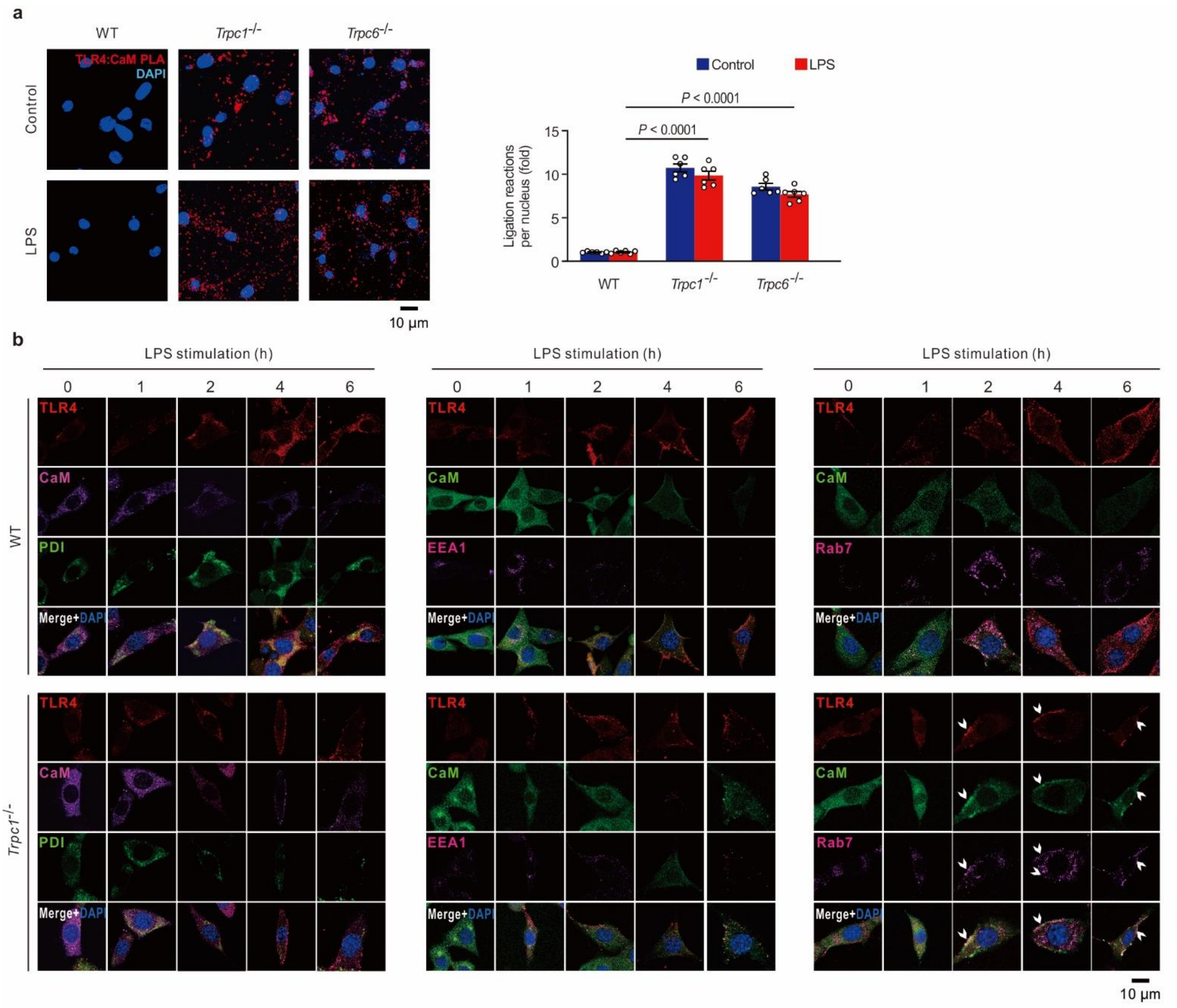

Supplementary Fig. 7 The intracellular interaction and location of CaM and TLR4 in cardiomyocytes. **a** Representative proximity ligation assay (PLA) photomicrographs (left panel) and their statistical analysis (right panel; mean ± SEM, n = 6 images from 3 biological independent experiments). Statistical significance was determined using the one-way ANOVA with Games Howell's multiple comparisons test. Exact *P* value = 6.7× 10^{-5} (WT+LPS vs *Trpc1*$^{-/-}$+LPS) and 2.6× 10^{-5} (WT+LPS vs *Trpc6*$^{-/-}$+LPS). **b** Representative immunofluorescent photomicrographs of TLR4 and CaM localization in the LPS-stimulated neonatal mice cardiomyocytes labeled with PDI (endoplasmic reticulum marker), EEA1 (early endosome marker), and Rab7 (late endosome marker). Blue indicates cellular nuclei stained with DAPI. Representative colocalization is indicated by white arrows (*n* = 6 images from 3 biological independent experiments). Source data are provided as a Source Data file.

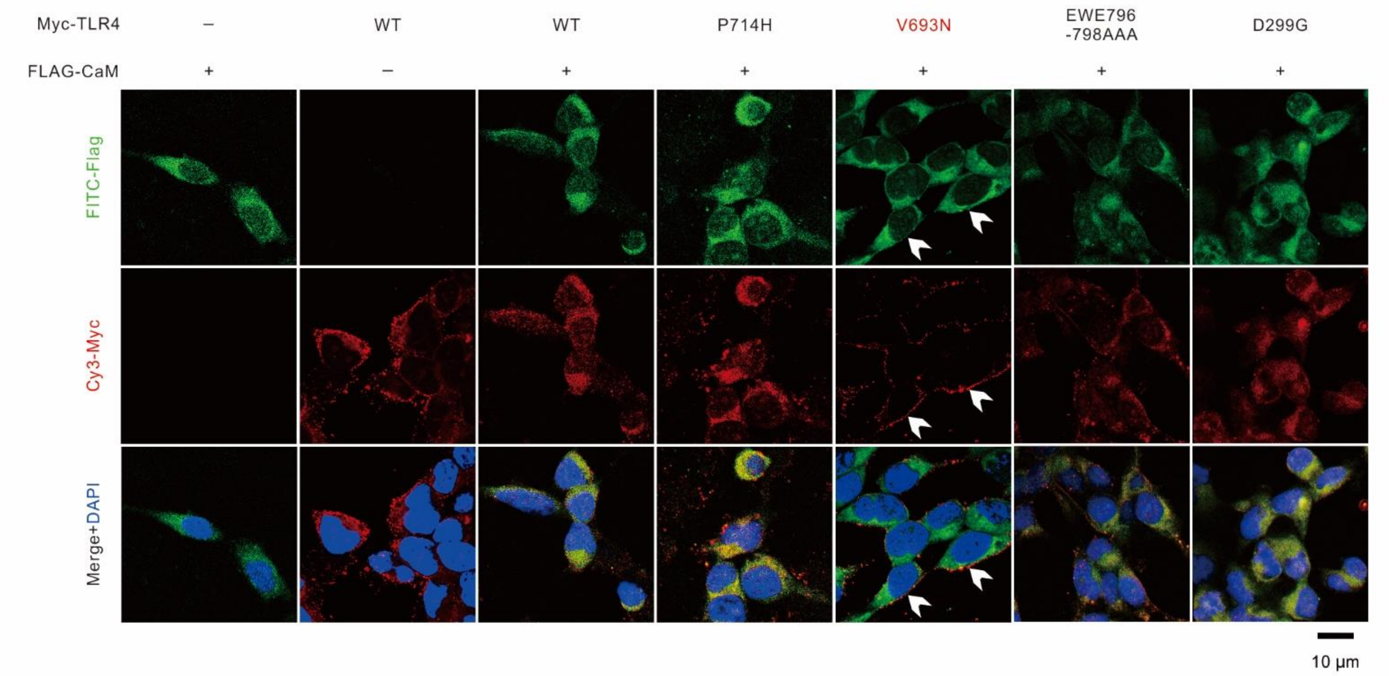

Supplementary Fig. 8 The CaM interaction with the TIR domain of TLR4. Immunofluorescence microscopy analysis of the HEK293T cells co-transfected with FLAG-tagged CaM and/or Myc-tagged TLR4 or its mutants. Representative TLR4 highly expressions in the cell surface are indicated by white arrows (n = 6 images from 3 biological independent experiments).

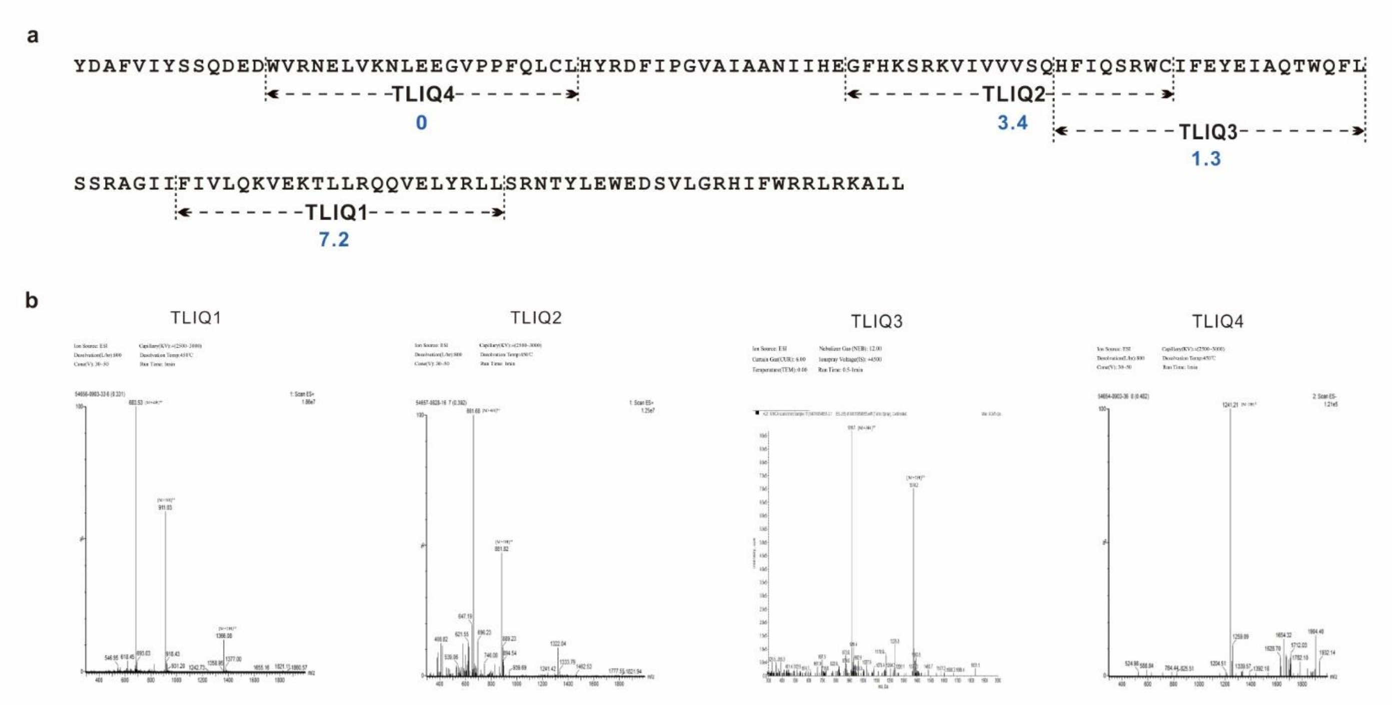

Supplementary Fig. 9 The synthesized peptides containing nonclassical IQ-like motifs within TIR domain of TLR4. a Four synthesized peptides (TLIQ1-4) encompassing the predicted structures of IQ motifs within the TIR domain of TLR4 using the CaM target database (http://calcium.uhnres.utoronto.ca/ctdb/ctdb/). The affinity-based docking scores for these motifs are shown in blue. **b** The mass spectrums of the synthesized peptides.

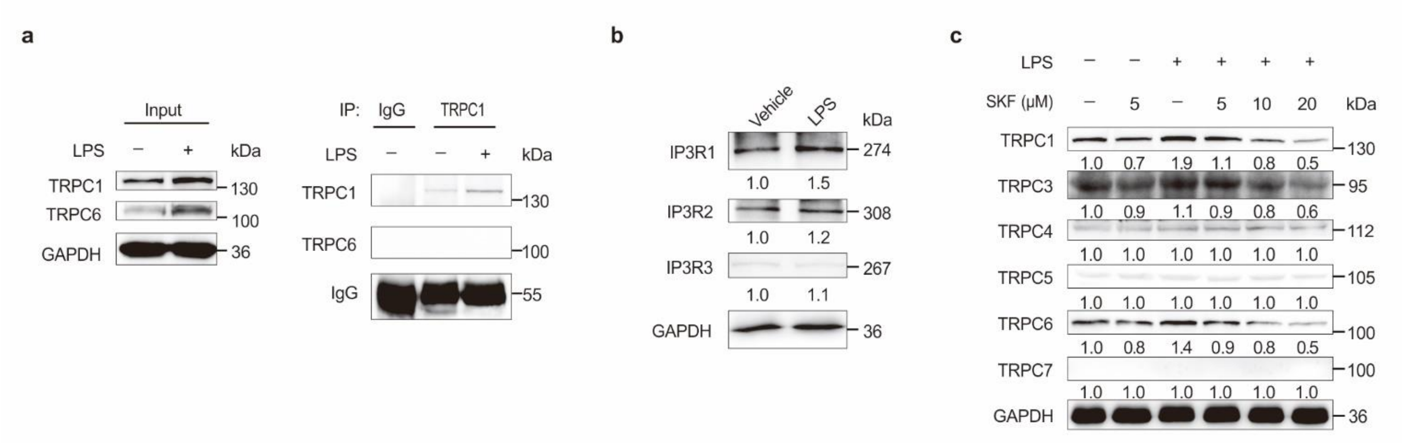

Supplementary Fig. 10 The pleiotropic roles of TRPC in regulating IP3R1 and the effects of SKF96365 (SKF) on the protein expressions of TRPCs. a Co-immunoprecipitation (Co-IP) analysis of TRPC1 binding with TRPC6 in neonatal mice cardiomyocytes 4 h after LPS challenge (*n* = 2 biological independent experiments). **b** The expressions of three IP3R subtypes in the hearts of mice challenged with LPS for 4 h (*n* = 3 biological independent experiments). **c** The effects of SKF on the protein expressions of TRPCs in LPS-challenged neonatal mice cardiomyocytes (*n* = 3 biological independent experiments). Statistical significance was determined using the one-way ANOVA with Tukey's multiple comparisons test. Source data are provided as a Source Data file.

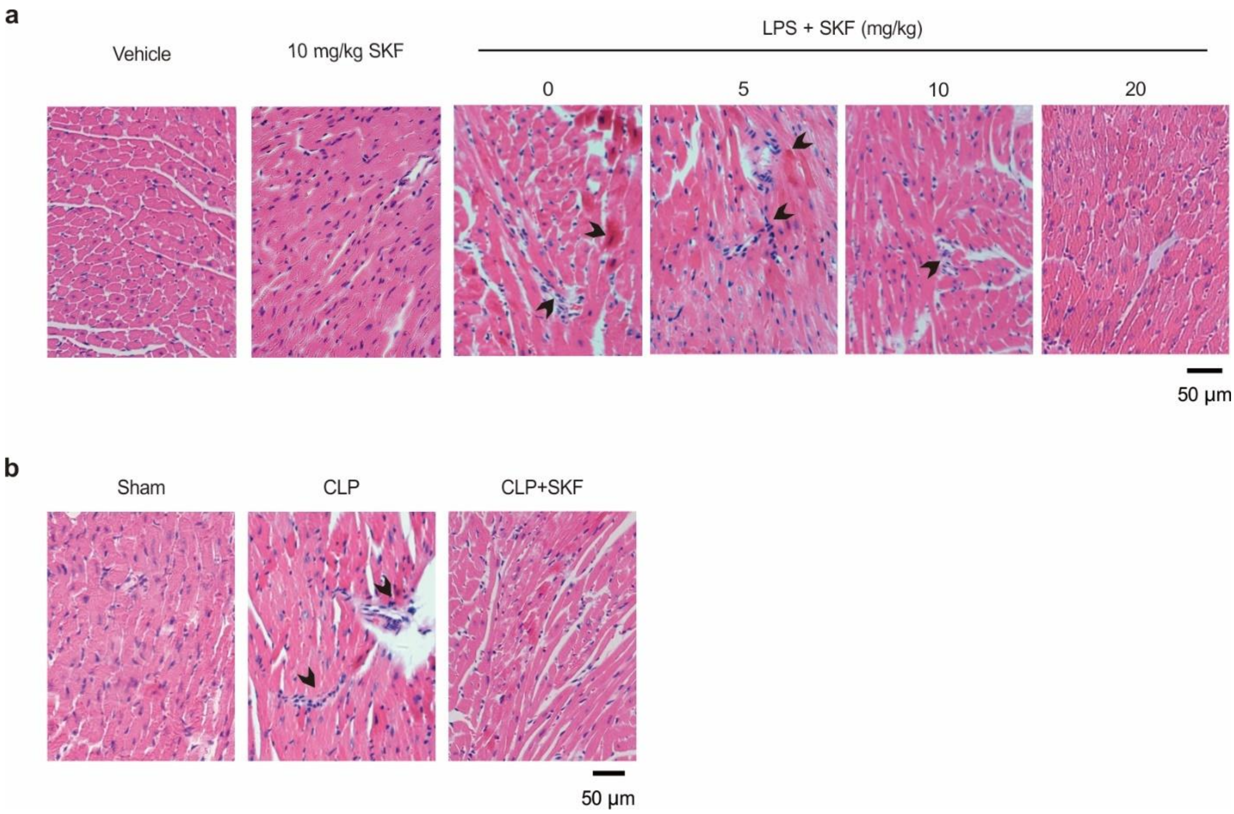

Supplementary Fig. 11 TRPC blocker SKF cures septic cardiac dysfunction. a Representative photomicrographs of ventricular tissues stained with hematoxylin and eosin (H&E) in the SKF-treated mice challenged with LPS for 6 h (n = 6 images from 3 male mice per group). **b** Representative photomicrographs of ventricular tissues stained with H&E in the SKF-treated mice performed cecal ligation and puncture (CLP) surgery for 6 h (n = 6 images from 3 male mice per group).

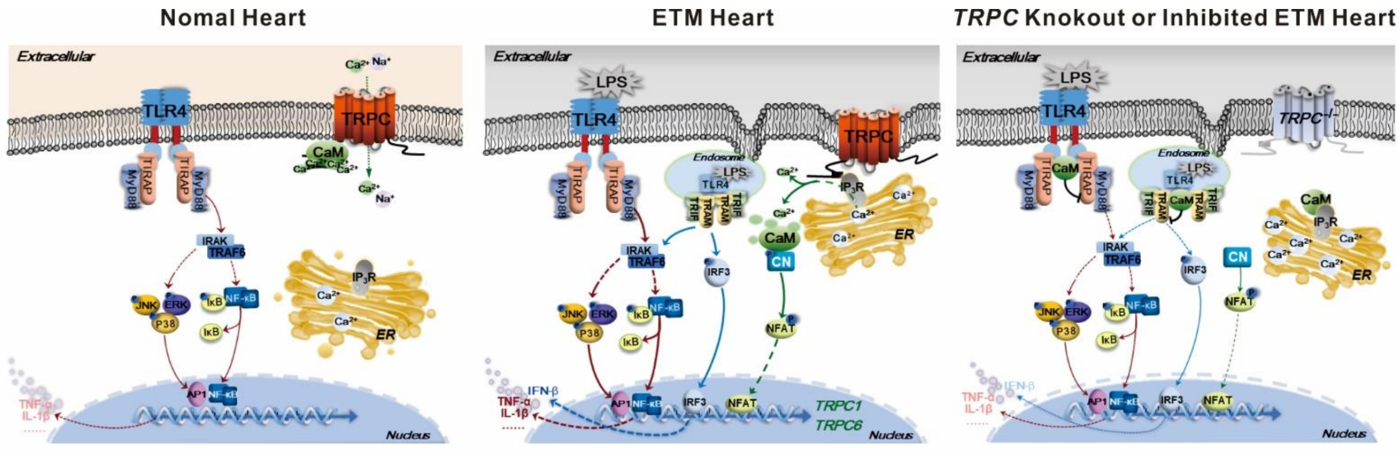

Supplementary Fig. 12 Schematic representation of the proposed mechanisms. In normal heart, LPS challenge stimulates the TLR4-mediated MAPK, NF-κB, and IRF3 activation to aggravate inflammation and simultaneously triggers elevated $[Ca^{2+}]_i$ via IP3R regulated by TRPCs, which in turn promotes NFAT to the nucleus to augment the TRPC and inflammatory genes expressions. Upon the TRPC knockout, Ca^{2+} leakage from ER is inhibited. In a parallel pathway, uncoupled CaM binds to TLR4 in the endosome and cytomembrane to interdict TIR domain, resulting in the inhibition of the MyD88 and TRIF-mediated inflammatory cascades.

Supplementary tables

Supplementary table 1

Echocardiographic assessment about left ventricular (LV) function of LPS-challenged *Trpc1*$^{-/-}$ or *Trpc6*$^{-/-}$ mice

Index	WT	WT+ LPS	*Trpc1*$^{-/-}$	*Trpc1*$^{-/-}$+LPS	*Trpc6*$^{-/-}$	*Trpc6*$^{-/-}$+LPS
BW (g)	23.54±0.55	23.74±0.46	23.37±0.83	24.33±0.73	22.87±0.41	22.96±0.47
IVST, d (mm)	0.75±0.04	0.66±0.04	0.70±0.07	0.75±0.08	0.82±0.13	0.75±0.07
IVST, s (mm)	1.13±0.11	0.84±0.05	1.07±0.11	0.92±0.09	1.17±0.14	0.97±0.10
LVEDD (mm)	3.24±0.17	3.93±0.30	3.24±0.16	3.37±0.11	3.28±0.29	3.55±0.15
LVESD (mm)	1.97±0.08*	3.43±0.27	2.16±0.10*	2.73±0.09	2.36±0.21	2.84±0.13
PWT, d (mm)	0.70±0.10	0.73±0.10	0.72±0.07	0.73±0.06	0.78±0.12	0.65±0.07
PWT, s (mm)	1.27±0.10	0.92±0.09	1.07±0.11	0.98±0.07	0.91±0.06	0.87±0.05
LV_{mass} (mg)	61.00±7.61	82.67±18.75	58.79±7.33	64.82±6.19	71.12±13.81	65.12±4.01
Mass/BW (mg/g)	2.60±0.32	3.49±0.78	2.50±0.28	2.66±0.24	3.10±0.60	2.85±0.20

BW, body weight; IVST, interventricular septal thickness; d, diastolic; s, systolic; LVEDD, left ventricular end-diastolic dimension; LVESD, end-systolic dimension; PWT, posterior wall thickness. n = 6 male mice per group. *P< 0.05 *vs*. LPS-challenged mice. Statistical significance was determined using one-way ANOVA with Game Howell's multiple comparisons test for IVST (d), LVESD, and LV_{mass}, and Tukey's multiple comparisons test for others. LVESD exact P value = 0.016 (WT vs WT+LPS) and 0.030 (WT+LPS vs *Trpc1*$^{-/-}$). Source data are provided as a Source Data file.

Supplementary table 2

The effects of LWMH on the LV function of LPS-challenged mice

Index	WT+ LPS	LMWH+ LPS
BW (g)	22.63±0.36	23.39±0.38
IVST, d (mm)	0.74±0.07	0.83±0.07
IVST, s (mm)	0.91±0.07	1.02±0.10
LVEDD (mm)	3.10±0.13	3.16±0.11
LVESD (mm)	2.65±0.13	2.67±0.12
PWT, d (mm)	0.61±0.09	0.67±0.05
PWT, s (mm)	0.77±0.06	0.82±0.07
LV_{mass} (mg)	49.38±5.58	59.31±6.30
Mass/BW (mg/g)	2.18±0.23	2.55±0.29

n = 6 male mice per group. Statistical significance was determined using the two-tailed Student's *t*-test. Source data are provided as a Source Data file.

Supplementary table 3

The effects of SKF96365 on the LV function of LPS-challenged mice

Index	Vehicle control	SKF (10 mg/kg)	LPS (50 mg/kg)	LPS+SKF (5 mg/kg)	LPS+SKF (10 mg/kg)	LPS+SKF (20 mg/kg)
BW (g)	22.07±0.60	22.25±0.19	22.02±0.44	22.47±0.51	21.70±0.65	22.23±0.50
IVST, d (mm)	0.70±0.06	0.75±0.12	0.86±0.18	0.58±0.04	0.59±0.04	0.84±0.11
IVST, s (mm)	0.99±0.04	0.97±0.14	0.90±0.14	0.68±0.09	0.86±0.07	1.01±0.13
LVEDD (mm)	2.92±0.12	3.18±0.25	3.36±0.23	3.40±0.19	3.24±0.15	2.96±0.16
LVESD (mm)	1.77±0.08*	2.11±0.16*	2.91±0.20	2.85±0.17	2.57±0.12	2.25±0.12*
PWT, d (mm)	0.78±0.12	0.82±0.04	0.88±0.21	0.68±0.03	0.68±0.05	0.77±0.12
PWT, s (mm)	1.22±0.19	1.19±0.10	1.04±0.28	0.97±0.06	0.89±0.06	0.96±0.12
LV_{mass} (mg)	53.12±9.58	67.59±12.66	88.79±29.25	53.49±7.42	48.76±3.85	62.25±12.99
Mass/BW (mg/g)	2.43±0.48	3.04±0.58	3.93±1.21	2.38±0.33	2.26±0.20	2.78±0.57

n = 6 male mice per group. **P*< 0.05 *vs*. LPS-challenged mice. Statistical significance was determined using one-way ANOVA with Game Howell's multiple comparisons test for IVST and PWT (d), and Tukey's multiple comparisons test for others. LVESD exact *P* value = 8.6×10^{-5} (vehicle control vs LPS), 0.0074 (WT+LPS vs SKF), and 0.037 (LPS vs LPS+ 20 mg/kg SKF). Source data are provided as a Source Data file.

Supplementary table 4

The effects of SKF96365 on the LV function of CLP mice

Index	Sham	CLP	CLP+SKF (10 mg/kg)
BW (g)	23.01±0.40	22.86±0.24	23.08±0.32
IVST, d (mm)	0.81±0.09	0.92±0.08	0.88±0.06
IVST, s (mm)	1.17±0.07	1.01±0.05	1.26±0.06*
LVEDD (mm)	3.16±0.14	2.94±0.27	2.83±0.19
LVESD (mm)	1.92±0.09	2.58±0.26	1.77±0.12
PWT, d (mm)	0.66±0.08	0.83±0.07	0.82±0.16
PWT, s (mm)	1.20±0.13	0.90±0.07	1.11±0.17
LV Mass (mg)	58.11±8.44	64.24±3.78	61.13±10.94
Mass/BW (mg/g)	2.51±0.34	2.69±0.50	2.79±0.19

n = 6 male mice per group. *P< 0.05 *vs*. CLP mice. Statistical significance was determined using one-way ANOVA with Game Howell's multiple comparisons test for LVESD, and Tukey's multiple comparisons test for others. IVST (s) exact P value = 0.032 (CLP vs CLP+SKF). Source data are provided as a Source Data file.

Supplementary Table 5 Virtual screening for the small-molecule antagonists of TRPC3 and TRPC6

		TRPC3				TRPC6			
Name	Structure	Total score	Crash	Polar	Schematic diagrams	Total score	Crash	Polar	Schematic diagrams
SKF-96365	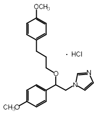	7.63	-2.06	2.41	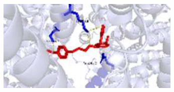	7.91	-0.69	2.15	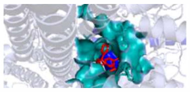
SH045	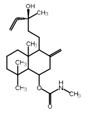	7.59	-1.04	3.05	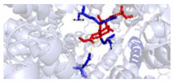	6.56	-1.05	2.11	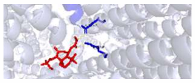
Larixyl acetate	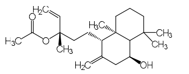	5.92	-1.64	2.41	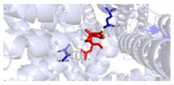	5.44	-1.14	2.03	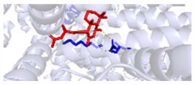
Pyr10	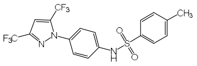	5.82	-1.09	1.13	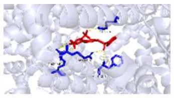	5.45	-0.89	2.38	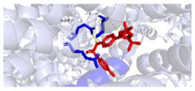
2-aminoeth-yester	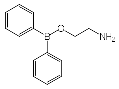	5.58	-1.26	1.03	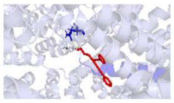	5.87	-1.25	2.22	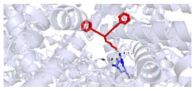
SAR7334	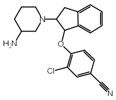	5.49	-1.46	3.04	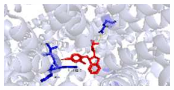	4.88	-2.20	1.37	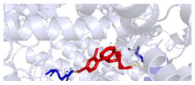
BI749327	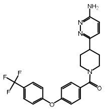	5.21	-1.08	2.42	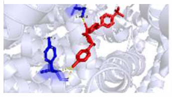	5.12	-2.31	2.52	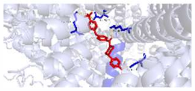
BTDM	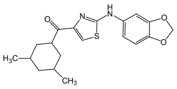	5.05	-1.63	2.92	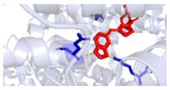	4.77	-1.33	0.78	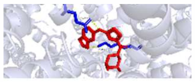
1,2,3-Thiadiazole-5-carboxa-mide	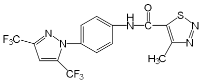	4.72	-0.67	2.69	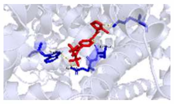	3.97	-0.81	0.47	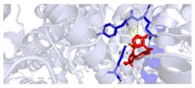
Anilino-thia-zoles	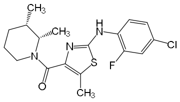	3.81	-1.51	2.06	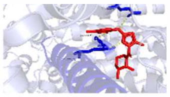	3.75	-1.36	1.88	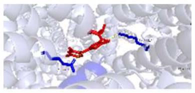
GSK2332255 B	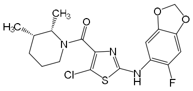	3.61	-2.53	2.07	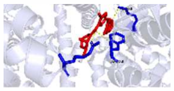	4.39	-2.03	1.82	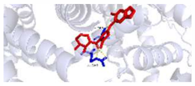
GSK2833503 A	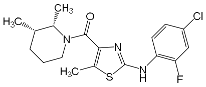	3.50	-1.70	2.61	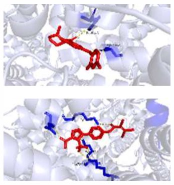	4.00	-0.70	3.01	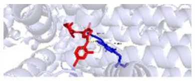
Pyr3	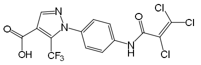	2.89	-2.07	1.52					
Pyrazolopy-rimidines	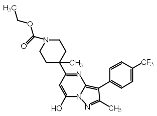					4.43	-1.01	1.04	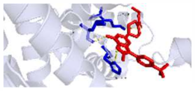

Supplementary table 6

Primer sequences for qPCR

Gene	Forward primer (5'-3')	Reverse primer (3'-5')
Tnfa	GAGTCCGGGCAGGTCTACTTT	CAGGTCACTGTCCCAGCATCT
Il1b	ACTGTTTCTAATGCCTTCCC	ATGGTTTCTTGTGACCCTGA
Il6	ACCACGGCCTTCCCTACTTC	CTCATTTCCACGATTTCCCAG
Tlr1	TTTGTCCCACAATGAGCTAAAGG	TTCTTTGCATATAGGCAGGGC
Tlr3	GAAGCAGGCGTCCTTGGACTT	TGTGCTGAATTCCGAGATCCA
Tlr4	GATCATGGCACTGTTCTTCTC	GGAATGTCATCAGGGACTTTG
Tlr6	AGCCAAGACAGAAAACCCATC	GGGGTCATGCTTCCGACTAT
Tlr9	ATGGTTCTCCGTCGAAGGACT	GAGGCTTCAGCTCACAGGG
Cxcl9	CTGTTCTTTTCCTCTTGGGCA	GGCAGGTTTGATCTCCGTTC
CD14	ACATCTTGAACCTCCGCAAC	AGGGTTCCTATCCAGCCTGT
Lbp	GGCTCTGCAGAGAGAGCTGTACAA	TAGTTAAGGAATGCCTGGAACAGG
Stat1	TCACAGTGGTTCGAGCTTCAG	CGAGACATCATAGGCAGCGTG
Irf5	AGAGACAGGGAAGTACACTGAAG	TGGAAGTCACGGCTTTTGTTAAG
Mapk13	ATGAGCCTCACTCGGAAAAGG	GCATGTGCTTCAAGAGCAGAA
Mapk12	CGCCGTGTACCAAGACCTG	GAGGCGCAACTCTCTGTAGG
Calm1	TAGCCACAACTCCTGAAACCA	CAGCACAGCCAAGACATCCT
Gapdh	CACTGAGCAAGAGAGGCCCTAT	GCAGCGAACTTTATTGATGGTATT

Supplementary table 7

siRNA oligonucleotides sequences

	**Sense**	**Anti-sense**
Itpr1-1	GGACGAGGCUGGAAAUGAA	UUCAUUUCCAGCCUCGUCC
Itpr1-2	GAGAUGAACUGGCAGAAGA	UCUUCUGCCAGUUCAUCUC
Itpr1-3	GUACAUGAGUUCUUCUAUA	UAUAGAAGAACUCAUGUAC
Itpr2-1	GUUCCCACUAUGACCUUAACU	AGUUAAGGUCAUAGUGGGAAC
Itpr2-2	CAUUCAGGAUGGUUACCUU	UAACAGCAUUCACCUCUUUGC
Itpr2-3	GUGAAAUACAGCAACGUUAUA	UAUAACGUUGCUGUAUUUCAC
Ryr2-1	GGAUCAUUGCGGUUCACUAUG	CAUAGUGAACCGCAAUGAUCC
Ryr2-2	CAGUUGUUGUUUAGAUCUAA	CUUAGAUCUAAACAACAACU
Ryr2-3	CGACGAAUUUCUCAGACAAGC	GCUUGUCUGAGAAAUUCGUCG
siRNA NC	UUCUCCGAACGUGUCACGU	ACGUGACACGUUCGGAGAA

Supplementary references

1. Mortazavi, A., et al. Mapping and quantifying mammalian transcriptomes by RNA-Seq. *Nat. Methods* **5**, 621-628 (2008).
2. Pathmanathan, S., et al. IQ motif selectivity in human IQGAP1: binding of myosin essential light chain and S100B. *Mol. Cell Biochem.* **318**, 43-51 (2008).
3. Kühl, T., et al. Analysis of Fe(III) heme binding to cysteine-containing heme-regulatory motifs in proteins. *ACS Chem. Biol.* **8**, 1785-1793 (2013).
4. Simon-Chica A, et al. Novel insights into the electrophysiology of murine cardiac macrophages: relevance of voltage-gated potassium channels. *Cardiovascular research* **118**, 798-813 (2022).
