# Supplementary material for: TRPC channels blockade abolishes endotoxemic cardiac dysfunction by hampering intracellular inflammation and Ca2+ leakage
Source: Nat Commun. 2022 Dec 2;13:7455. doi: 10.1038/s41467-022-35242-0 (PMC9718841; doi:10.1038/s41467-022-35242-0)
Supplement: Supplementary file 2 — Reporting Summary [file 41467_2022_35242_MOESM2_ESM.pdf]

nature portfolio

Corresponding author(s): Wei Cao, Xiao-Qiang Li

Last updated by author(s): Nov 6, 2022

Reporting Summary

Nature Portfolio wishes to improve the reproducibility of the work that we publish. This form provides structure for consistency and transparency in reporting. For further information on Nature Portfolio policies, see our Editorial Policies and the Editorial Policy Checklist.

Statistics

For all statistical analyses, confirm that the following items are present in the figure legend, table legend, main text, or Methods section.

n/a	Confirmed	
☐	☒	The exact sample size (n) for each experimental group/condition, given as a discrete number and unit of measurement
☐	☒	A statement on whether measurements were taken from distinct samples or whether the same sample was measured repeatedly
☐	☒	The statistical test(s) used AND whether they are one- or two-sided<br>*Only common tests should be described solely by name; describe more complex techniques in the Methods section.*
☒	☐	A description of all covariates tested
☐	☒	A description of any assumptions or corrections, such as tests of normality and adjustment for multiple comparisons
☐	☒	A full description of the statistical parameters including central tendency (e.g. means) or other basic estimates (e.g. regression coefficient) AND variation (e.g. standard deviation) or associated estimates of uncertainty (e.g. confidence intervals)
☐	☒	For null hypothesis testing, the test statistic (e.g. F, t, r) with confidence intervals, effect sizes, degrees of freedom and P value noted<br>*Give P values as exact values whenever suitable.*
☒	☐	For Bayesian analysis, information on the choice of priors and Markov chain Monte Carlo settings
☒	☐	For hierarchical and complex designs, identification of the appropriate level for tests and full reporting of outcomes
☒	☐	Estimates of effect sizes (e.g. Cohen's d, Pearson's r), indicating how they were calculated

Our web collection on statistics for biologists contains articles on many of the points above.

Software and code

Policy information about availability of computer code

Data collection	Echocardiography assessment of cardiac function was performed using a Vevo-2100 high-resolution imaging unit (Visual Sonics, Toronto, Ontario, Canada). Mean arterial blood pressure data were collected by a precalibrated PowerLab/4SP recording system (AD Instruments Pty Ltd., Bella Vista, New South Walles, Australia). Immunohistochemistry of ventricular tissues was examined and photographed with a Nikon Eclipse 80i microscope (magnification ×400). Immunofluorescence images of ventricular tissues, cells and proximity ligation assay images were obtained using a confocal microscopy (FV3000, Olympus, Tokyo, Japan). Exposure of immunoblotting was performed in a Tanon-5200 imaging system (Tanon Science and Technology Co., Ltd., Shanghai, China). Intracellular calcium concentration was measured using a digital wide-field fluorescence imaging system (TILL Photonics GmbH, Gräfelfing, Germany). High throughput sequencing was performed on an Illumina instrument (Illumina HiSeq™ 4000, San Francisco, USA) in 150 nt paired-end configuration. Real-time PCR was performed on ViiA 7 Real-time PCR system (Applied Biosystems, Foster city, CA, USA). Microscale thermophoresis was measured by a Monolith NT.115 instrument (NanoTemper Technologies, Germany). Circular dichroism spectra were recorded on a Chirascan spectropolarimeter (Applied Photophysics, Surrey, UK). Cardiac-resident macrophages were FACS-purified using an Aria III cell sorter (BD Biosciences, Franklin Lakes, NJ, USA). Optical density values were collected on a Synergy HTX multi-mode microplate reader (BioTek, Winooski, USA).

Data analysis	Quantifications of immunoblotting, immunofluorescence staining, and proximity ligation assay spots were analyzed by Image J (version 1.49). In RNA-seq, image analysis and base calling were performed using Solexa pipeline V1.8 (Off-Line Base Caller software, version 1.8); Trimmed reads (pass FastQC 0.11.5 filter) were aligned to the mouse reference genome (GenCode mm10) and the mouse transcriptome (GenCode mm10) using Hisat2 software (version 2.0.5); Transcriptional abundance estimation was completed via StingTie software (version 1.3.1c) according to the official database annotation information; The gene & transcript expression levels (FPKM value) and significant changes in gene & transcript expression were calculated using Ballgown (version 2.8.4). Statistical analysis was performed with the SPSS Statistics v.23.0 (IBM Corp.). Virtual screening was performed using the Surflex-Dock of SYBYL-X 2.0 (Tripos). Protein-protein docking was studied by Z dock protocol in Discovery Studio 4.0 (Accelrys). KD values of microscale thermophoresis were analyzed and fitted by the NT analysis software v.2.2.4 (NanoTemper Technologies). FlowJo software (V10.0.7) was used to analyze FACS data. The OriginPro 2018C software (OriginLab Corp.) was used for ELISA data analysis.

For manuscripts utilizing custom algorithms or software that are central to the research but not yet described in published literature, software must be made available to editors and reviewers. We strongly encourage code deposition in a community repository (e.g. GitHub). See the Nature Portfolio guidelines for submitting code & software for further information.

Data

Policy information about availability of data

All manuscripts must include a data availability statement. This statement should provide the following information, where applicable:

- Accession codes, unique identifiers, or web links for publicly available datasets
- A description of any restrictions on data availability
- For clinical datasets or third party data, please ensure that the statement adheres to our policy

The RNA-seq data generated in this study have been deposited in the GEO database (https://www.ncbi.nlm.nih.gov/geo/) under accession code GSE217156. Information of the structure of proteins TLR2 (PDB code 1FYX), CaM (PDB code 1QX5), TRPC6 (PDB code 5YX9), TIRAP (PDB code 4FZ5), and TRPC3 (PDB code 5ZBG) was obtained from the PDB database (/https://www.rcsb.org/). The amino acid sequence of cytoplasmic domain of human TLR4 (Uniport accession code O00206) was obtained from the Uniport database (https://www.uniprot.org/). The authors declare that all other data generated in this study are provided in the supplementary information and source data file.

Field-specific reporting

Please select the one below that is the best fit for your research. If you are not sure, read the appropriate sections before making your selection.

☒ Life sciences ☐ Behavioural & social sciences ☐ Ecological, evolutionary & environmental sciences

For a reference copy of the document with all sections, see nature.com/documents/nr-reporting-summary-flat.pdf

Life sciences study design

All studies must disclose on these points even when the disclosure is negative.

Sample size	No statistical method was used to predetermine samples size. Sample size was large enough to determine statistically significant effects.
Data exclusions	No data was excluded from analysis.
Replication	All experiments were performed with at least three independent biological samples. All attempts at replication were successful.
Randomization	Mice were matched by genotype and age, and then randomly assigned to specific treatment groups.
Blinding	Blinding is not relevant because all experiment groups were conducted with different treatments.

Reporting for specific materials, systems and methods

We require information from authors about some types of materials, experimental systems and methods used in many studies. Here, indicate whether each material, system or method listed is relevant to your study. If you are not sure if a list item applies to your research, read the appropriate section before selecting a response.

Materials & experimental systems

n/a	Involved in the study
	x Antibodies
	x Eukaryotic cell lines
x	Palaeontology and archaeology
	x Animals and other organisms
x	Human research participants
x	Clinical data
x	Dual use research of concern

Methods

n/a	Involved in the study
x	ChIP-seq
	x Flow cytometry
x	MRI-based neuroimaging

Antibodies

Antibodies used

TRPC1 (Alomone, ACC-010, 1:200 for WB, 1:100 for immunofluorescence, 4 μg Ab per 1000 μg protein for immunoprecipitation); TRPC3 (Alomone, ACC-016, 1:200 for WB); TRPC4 (Alomone, ACC-018, 1:200 for WB); TRPC5 (Alomone, ACC-020, 1:200 for WB); TRPC6 (Alomone, ACC-017, 1:200 for WB, 1:100 for immunofluorescence, 4 μg Ab per 1000 μg protein for immunoprecipitation); TRPC7 (Alomone, ACC-066, 1:200 for WB); GAPDH (Proteintech, 60004-1-Ig, clone 1E6D9, 1:5000 for WB); CCL3/MIP-1α (R&D systems, AF-450-SP, 1:50 for immunohistochemistry); NF-κB p65 (Proteintech, 10745-1-AP, 1:1000 for WB); Histone (Proteintech, 17168-1-AP, 1:1000 for WB); α-Tubulin (Proteintech, 66031-1-Ig, clone 1E4C11, 1:1000 for WB); p-ERK1/2 (Cell signalling, #4370, clone D13.14.4E, 1:1000 for WB); ERK1/2 (Cell signalling, #4695, clone 137F5, 1:1000 for WB); p-JNK (Cell signalling, #4668, clone 81E11, 1:1000 for WB); JNK (Cell signalling, #9252, 1:1000 for WB); p-p38 (Cell signalling, #4511, clone D3F9, 1:1000 for WB); p38 (Cell signalling, #8690, clone D13E1, 1:1000 for WB); TLR4 (Santa cruz, sc-293072, clone 25, 1:1000 for WB; 1:300 for immunofluorescence, 4 μg Ab per 1000 μg protein for immunoprecipitation); MyD88 (Santa cruz, sc-74532, clone E-11, 1:1000 for WB); TRIF (Santa cruz, sc-514384, clone E-7, 1:1000 for WB); TRAM (Proteintech, 12705-1-AP, 1:1000 for WB); TIRAP (Cell signalling, #13077, clone D6M9Z, 1:1000 for WB); p-IRAK1 (Abbkine, ABP54916, 1:1000 for WB); IRAK1 (Proteintech, 10478-2-AP, 1:1000 for WB); IRAK4 (Proteintech, 18221-1-AP, 1:1000 for WB); TRAF6 (Santa cruz, sc-8409, clone D-10, 1:3000 for WB); p-IRF-3 (Biorbyt, orb571465, 1:1000 for WB); IRF-3 (Proteintech, 11312-1-AP, 1:1000 for WB); CaM (Santa cruz, sc-137079, clone G-3, 1:1000 for WB, 1:300 for immunofluorescence); NFAT3 (Cell signalling, #2183, clone 23E6, 1:1000 for WB); FLAG tag (Proteintech, 20543-1-AP, 1:3000 for WB, 1:100 for immunofluorescence); Myc tag (Proteintech, 60003-2-Ig, clone 1A5A2, 1:3000 for WB, 4 μg Ab per 1000 μg protein for immunoprecipitation, 1:500 for immunofluorescence); IP3R1 (Abcam, ab264281, 1:2000 for WB, 1:300 for immunofluorescence); IP3R2 (Santa cruz, sc-398434, clone A-5, 1:1000 for WB); IP3R3 (Cohesion, CQA4813, 1:1000 for WB); RYR2 (SAB, #48554, 1:500 for WB); PDI (Cell signalling, #3501, clone C81H6, 1:100 for immunofluorescence); Rab7 (Santa cruz, sc-376362, clone B-3, 1:300 for immunofluorescence); EEA1 (Santa cruz, sc-365652, clone E-8, 1:300 for immunofluorescence); TRPC1 (Proteintech, 19482-1-AP, 1:100 for immunofluorescence); TRPC1 (Santa cruz, sc-133076, clone E-6, 1:400 for immunofluorescence); TRPC6 (Proteintech, 18236-1-AP, 1:100 for immunofluorescence); TRPC6 (Santa cruz, sc-515837, clone B-10, 1:400 for immunofluorescence); CaM (Abcam, ab45689, clone EP799Y, 1:2000 for WB); α-SCA (Sigma, SAB4200689, clone 5C5, 1:500 for immunofluorescence); CD68 (Abcam, ab955, clone KP1, 1:50 for immunofluorescence); DDR2 (Santa cruz, sc-81707, clone 3B11E4, 1:200 for immunofluorescence); FITC-labeled goat anti-rabbit IgG (Jackson Immunoresearch Laboratories, 111-095-144; 1:100 for immunofluorescence); FITC-labeled goat anti-mouse IgG (Jackson Immunoresearch Laboratories, 115-095-062; 1:100 for immunofluorescence); Cy3-labeled goat anti-rabbit IgG (Jackson Immunoresearch Laboratories, 111-165-144 1:400 for immunofluorescence); Cy3-labeled goat anti-mouse IgG (Jackson Immunoresearch Laboratories, 115-165-003 1:400 for immunofluorescence); Alexa fluor 350-labeled goat Anti-rabbit IgG (Invitrogen, 11046, 1:2000 for immunofluorescence); Alexa fluor 647-labeled goat anti-mouse IgG (Jackson Immunoresearch Laboratories, 115-605-003, 1:400 for immunofluorescence); anti-mouse IgG Ab (Beyotime, A7028, 4 μg Ab per 1000 μg protein for immunoprecipitation); anti-rabbit IgG Ab (Beyotime, A7016, 4 μg Ab per 1000 μg protein for immunoprecipitation); mouse anti-rabbit IgG light chain Ab (Abbkine, A25022, 1:2000 for WB); horseradish peroxidase (HRP)-conjugated goat anti mouse secondary Ab (Abbkine, A21010, 1:10000 for WB); HRP-conjugated goat anti rabbit secondary Ab (Abbkine, A21020, 1:10000 for WB); CD45.2-APC (eBioscience, 17-0454-81, 0.5 μg Ab per 10^7 cells for fluorescence-activated cell sorting, FACS); F4/80-PE (eBioscience, 12-4801-80, 0.25 μg Ab per 10^7 cells for FACS); CD11b-PerCP.Cy5.5 (eBioscience, 45-0112-80, 0.25 μg Ab per 10^7 cells for FACS).

Validation

Antibody validation information can be found on manufacturers' website:
TRPC1 rabbit Ab: https://www.alomone.com/p/anti-trpc1/ACC-010
TRPC3 rabbit Ab: https://www.alomone.com/p/anti-trpc3-2/ACC-016
TRPC4 rabbit Ab: https://www.alomone.com/p/anti-trpc4/ACC-018
TRPC5 rabbit Ab: https://www.alomone.com/p/anti-trpc5/ACC-020
TRPC6 rabbit Ab: https://www.alomone.com/p/anti-trpc6/ACC-017
TRPC7 rabbit Ab: https://www.alomone.com/p/anti-trpc7-extracellular/ACC-066
GAPDH mouse Ab: https://www.ptgcn.com/products/GAPDH-Antibody-60004-1-Ig.htm
CCL3/MIP-1α mouse Ab: https://www.rndsystems.com/cn/products/mouse-ccl3-mip-1alpha-antibody_af-450-na
NF-κBp65 rabbit Ab: https://www.ptgcn.com/products/p65-Antibody-10745-1-AP.htm
Histone rabbit Ab: https://www.ptgcn.com/products/Histone-H3-Antibody-17168-1-AP.htm
α-Tubulin mouse Ab: https://www.ptgcn.com/products/tubulin-Alpha-Antibody-66031-1-Ig.htm
p-ERK1/2 rabbit Ab: https://www.cellsignal.cn/products/primary-antibodies/phospho-p44-42-mapk-erk1-2-thr202-tyr204-d13-14-4e-xp-rabbit-mab/4370
ERK1/2 rabbit Ab: https://www.cellsignal.cn/products/primary-antibodies/p44-42-mapk-erk1-2-137f5-rabbit-mab/4695
p-JNK rabbit Ab: https://www.cellsignal.cn/products/primary-antibodies/phospho-sapk-jnk-thr183-tyr185-81e11-rabbit-mab/4668
JNK rabbit Ab: https://www.cellsignal.cn/products/primary-antibodies/sapk-jnk-antibody/9252
p-p38 rabbit Ab: https://www.cellsignal.cn/products/primary-antibodies/phospho-p38-mapk-thr180-tyr182-d3f9-xp-rabbit-mab/4511

p38 MAPK rabbit Ab: https://www.cellsignal.cn/products/primary-antibodies/p38-mapk-d13e1-xp-rabbit-mab/8690
TLR4 mouse Ab: https://www.scbt.com/zh/p/tlr4-antibody-25?requestFrom=search
MyD88 mouse Ab: https://www.scbt.com/p/myd88-antibody-e-11?requestFrom=search
IRAK4 rabbit Ab: https://www.ptgcn.com/products/IRAK4-Antibody-18221-1-AP.htm
TRAF6 mouse Ab: https://www.scbt.com/zh/p/traf6-antibody-d-10?requestFrom=search
p-IRAK1 rabbit Ab: https://www.amyjet.com/products/ABP54916.shtml
IRAK1 rabbit Ab: https://www.ptgcn.com/products/IRAK1-Antibody-10478-2-AP.htm
p-IRF3 rabbit Ab: https://biorbyt.com.cn/irf3-phospho-s396-antibody-orb571465.html
IRF3 rabbit Ab: https://www.ptgcn.com/products/IRF3-Antibody-11312-1-AP.htm
CaM mouse Ab: https://www.scbt.com/p/cam-antibody-g-3?requestFrom=search
NFAT3 rabbit Ab: https://www.cellsignal.cn/products/primary-antibodies/nfat3-23e6-rabbit-mab/2183?site-search-type=Products&N=4294956287&Ntt=nfat3+%28%232183%29&fromPage=plp&_requestid=7749898
TIRAP rabbit Ab: https://www.cellsignal.cn/products/primary-antibodies/tirap-d6m9z-rabbit-mab-mouse-specific/13077?site-search-type=Products&N=4294956287&Ntt=13077&fromPage=plp&_requestid=7751246
TRAM rabbit Ab: https://www.ptgcn.com/products/TRAM1-Antibody-12705-1-AP.htm
TRIF mouse Ab: https://www.scbt.com/p/ticam-1-antibody-e-7?requestFrom=search
Cam rabbit Ab: https://www.abcam.cn/calmodulin-123-antibody-ep799y-c-terminal-ab45689.html
TRPC1 mouse Ab: https://www.scbt.com/p/trpc1-antibody-e-6?requestFrom=search
TRPC6 mouse Ab: https://www.scbt.com/zh/p/trpc6-antibody-b-10?requestFrom=search
TRPC1 rabbit Ab: https://www.ptgcn.com/products/TRPC1-Antibody-19482-1-AP.htm
TRPC6 rabbit Ab: https://www.ptgcn.com/products/TRPC6-Antibody-18236-1-AP.htm
PDI rabbit Ab: https://www.cellsignal.cn/products/primary-antibodies/pdi-c81h6-rabbit-mab/3501?site-search-type=Products&N=4294956287&Ntt=%233501&fromPage=plp&_requestid=7756149
Rab7 mouse Ab: https://www.scbt.com/p/rab-7-antibody-b-3?requestFrom=search
EEA1 mouse Ab: https://www.scbt.com/p/eea1-antibody-e-8?requestFrom=search
α-SCA mouse Ab: https://www.sigmaaldrich.cn/CN/en/product/sigma/sab4200689
CD68 mouse Ab: https://www.abcam.cn/cd68-antibody-kp1-ab955.html
DDR2 mouse Ab: https://www.scbt.com/p/ddr2-antibody-3b11e4?requestFrom=search
FLAG tag rabbit Ab: https://www.ptgcn.com/products/Flag-Tag-Antibody-20543-1-AP.htm
Myc tag rabbit Ab: https://www.ptgcn.com/products/MYC-Antibody-60003-2-Ig.htm
IP3R1 rabbit Ab: https://www.abcam.cn/ip3r1-antibody-ab264281.html#lb
IP3R2 mouse Ab: https://www.scbt.com/p/ip3r-ii-antibody-a-5?requestFrom=search
IP3R3 rabbit Ab: http://www.cohesionbio.com/Primary-Antibody/72276.html
RYR2 rabbit Ab: https://www.sabbiotech.com.cn/g-151462-RYR2-Antibody-48554.html
CD45.2-APC: https://www.thermofisher.cn/cn/zh/antibody/product/CD45-2-Antibody-clone-104-Monoclonal/17-0454-81
F4/80-PE: https://www.thermofisher.cn/cn/zh/antibody/product/F4-80-Antibody-clone-BM8-Monoclonal/12-4801-80
CD11b-PerCP.Cy5.5: https://www.thermofisher.cn/cn/zh/antibody/product/CD11b-Antibody-clone-M1-70-Monoclonal/45-0112-80

Eukaryotic cell lines

Policy information about cell lines

Cell line source(s)	293T cells (ATCC CRL-3216)
Authentication	293T cell line was authenticated using Short Tandem Repeat analysis as described in 2012 in ANSI Standard (ASN-0002) by the ATCC Standards Development Organization.
Mycoplasma contamination	All cells were tested for mycoplasma contamination periodically. The results were always negative for mycoplasma contamination.
Commonly misidentified lines (See ICLAC register)	None.

Animals and other organisms

Policy information about studies involving animals; ARRIVE guidelines recommended for reporting animal research

Laboratory animals	Trpc1 knockout and Trpc6 knockout mice purchased from the Jackson Laboratory (Bar Harbor, ME, USA) had been backcrossed > 10 generations into the C57BL/6 strain and maintained at the Fourth Military Medical University (FMMU). WT C57BL/6 mice were purchased from the Laboratory Animal Center of FMMU. 2 months old male WT, Trpc1 knockout, and Trpc6 knockout mice, and 2- to 3-d-old WT, Trpc1 knockout, and Trpc6 knockout neonatal mice were used in the experiments.
Wild animals	This study did not involve wild animals.
Field-collected samples	No sample was collected from the field.
Ethics oversight	All of the animal care and handling procedures were carried out in accordance with the recommendations in the Guide for the Care and Use of Laboratory Animals of the National Institutes of Health. The protocol was approved by the Committee on the Ethics of

Animal Experiments of FMMU (permit number, XJYYLL-2014484). The mice used in the study had the following housing conditions: humidity, 50-60%; temperature, 22–24°C; dark/light cycle, 12h dark/12h light.

Note that full information on the approval of the study protocol must also be provided in the manuscript.

Flow Cytometry

Plots

Confirm that:

- [x] The axis labels state the marker and fluorochrome used (e.g. CD4-FITC).
- [x] The axis scales are clearly visible. Include numbers along axes only for bottom left plot of group (a 'group' is an analysis of identical markers).
- [x] All plots are contour plots with outliers or pseudocolor plots.
- [x] A numerical value for number of cells or percentage (with statistics) is provided.

Methodology

Sample preparation	Mouse cardiac-resident macrophages (cMacs) were obtained from WT, Trpc1 knockout, and Trpc6 knockout male mice (2 months old). Hearts were swiftly excised and gently flushed with 20 mL cold PBS. Thereafter, tissue was minced into small pieces and subjected to enzymatic digestion with 450 U/mL collagenase I, 125 U/mL collagenase XI, 60 U/mL DNase I, and 60 U/mL hyaluronidase in 1 mL PBS for 35 min at 37°C under gentle agitation. Tissue fragments were then triturated, filtered through a 40 mm nylon mesh, and pelleted by centrifugation (400×g for 5 min at 4°C). Cells were re-suspended in fluorescence-activated cell sorting (FACS) buffer (PBS with 1% foetal calf serum + 0.1% bovine serum albumin).
Instrument	Aria III cell sorter (BD Biosciences, Franklin Lakes, NJ, USA)
Software	FlowJo VX10.0.7 software (TreeStar, Ashland, OR, USA)
Cell population abundance	cMacs were sorted based on CD45+ F4/80+ CD11b+ and the cell population was about 0.1%.
Gating strategy	Cells were gated based on SSC-A and CD45+ to identify the leukocytes, and then viable cMacs were selected from leukocytes by gating for F4/80+ CD11b+ cells.

- [x] Tick this box to confirm that a figure exemplifying the gating strategy is provided in the Supplementary Information.
